# A novel nutritional supplement to reduce plasma homocysteine in nonpregnant women: A randomised controlled trial in The Gambia

**DOI:** 10.1371/journal.pmed.1002870

**Published:** 2019-08-13

**Authors:** Philip T. James, Ousubie Jawla, Nuredin I. Mohammed, Kabiru Ceesay, Fatai M. Akemokwe, Bakary Sonko, Ebrima A. Sise, Andrew M. Prentice, Matt J. Silver

**Affiliations:** Medical Research Council Unit The Gambia at the London School of Hygiene and Tropical Medicine, London, United Kingdom; Peking University First Hospital, CHINA

## Abstract

**Background:**

Infant DNA methylation profiles are associated with their mother’s periconceptional nutritional status. DNA methylation relies on nutritional inputs for one-carbon metabolic pathways, including the efficient recycling of homocysteine. This randomised controlled trial in nonpregnant women in rural Gambia tests the efficacy of a novel nutritional supplement designed to improve one-carbon-related nutrient status by reducing plasma homocysteine, and assesses its potential future use in preconception trials.

**Methods and findings:**

We designed a novel drink powder based on determinants of plasma homocysteine in the target population and tested it in a three-arm, randomised, controlled trial. Nonpregnant women aged between 18 and 45 from the West Kiang region of The Gambia were randomised in a 1:1:1 allocation to 12 weeks daily supplementation of either (a) a novel drink powder (4 g betaine, 800 μg folic acid, 5.2 μg vitamin B12, and 2.8 mg vitamin B2), (b) a widely used multiple micronutrient tablet (United Nations Multiple Micronutrient Preparation [UNIMMAP]) containing 15 micronutrients, or (c) no intervention. The trial was conducted between March and July 2018. Supplementation was observed daily. Fasted venepuncture samples were collected at baseline, midline (week 5), and endline (week 12) to measure plasma homocysteine. We used linear regression models to determine the difference in homocysteine between pairs of trial arms at midline and endline, adjusted for baseline homocysteine, age, and body mass index (BMI). Blood pressure and pulse were measured as secondary outcomes. Two hundred and ninety-eight eligible women were enrolled and randomised. Compliance was >97.8% for both interventions. At endline (our primary endpoint), the drink powder and UNIMMAP reduced mean plasma homocysteine by 23.6% (−29.5 to −17.1) and 15.5% (−21.2 to −9.4), respectively (both *p* < 0.001), compared with the controls. Compared with UNIMMAP, the drink powder reduced mean homocysteine by 8.8% (−15.8 to −1.2; *p* = 0.025). The effects were stronger at midline. There was no effect of either intervention on blood pressure or pulse compared with the control at endline. Self-reported adverse events (AEs) were similar in both intervention arms. There were two serious AEs reported over the trial duration, both in the drink powder arm, but judged to be unrelated to the intervention. Limitations of the study include the use of a single targeted metabolic outcome, homocysteine.

**Conclusions:**

The trial confirms that dietary supplements can influence metabolic pathways that we have shown in previous studies to predict offspring DNA methylation. Both supplements reduced homocysteine effectively and remain potential candidates for future epigenetic trials in pregnancy in rural Gambia.

**Trial registration:**

Clinicaltrials.gov Reference NCT03431597.

## Abstract 2

Philip Thomas James and colleagues reveal a potential, unique link between mother’ nutritional input and an offspring’s methylation status.

## Author summary

**Why was this study done?:**

- At conception, the methylation marks on DNA from sperm and egg are erased and then re-established in the very early embryo.
- This DNA methylation relies on nutritional inputs for one-carbon metabolic pathways, including the efficient recycling of homocysteine, an absence of which might interfere with normal fetal development.
- We sought to test nutritional supplements that could reduce homocysteine and thereby optimise methylation pathways in women at the time of conception.

**What did the researchers do and find?:**

- We designed a novel drink powder containing betaine, folic acid, and vitamins B12 and B2 and tested its efficacy in reducing plasma homocysteine.
- Efficacy was tested in nonpregnant rural Gambian women in a randomised controlled trial, in which we also tested the UNIMMAP supplement that contains 15 micronutrients but no betaine.
- Both our novel supplement and UNIMMAP reduced homocysteine after 5 and 12 weeks of supplementation, with the novel drink powder showing a significantly enhanced efficacy.

**What do these findings mean?:**

- This trial demonstrates that it is feasible to lower homocysteine levels even in women whose homocysteine levels are only marginally elevated.
- Our novel supplement would be a potential candidate for future trials of preconceptional supplements aimed at optimising offspring DNA methylation.

## Introduction

Parental nutritional status at the time of conception can influence the lifelong health and disease risk of the developing child [1,2]. Epigenetic modifications provide a plausible mechanism to explain some of these associations [3]. Multiple maternal factors may affect the developing fetal epigenome in early gestation, with nutritional one-carbon-related metabolites being of particular interest [2,4,5]. One-carbon metabolism encompasses metabolic pathways crucial for the provision of methyl groups required for DNA methylation. These pathways involve folate, methionine, serine, glycine, choline, and betaine for methyl group donation, and vitamins B2, B12, and B6 as essential cofactors.

This raises an important public health question: can improving periconceptional nutritional status by providing the nutrients known to support epigenetic processes lead to improved offspring health outcomes? To answer this, a crucial first step is to understand what such a preconception nutritional supplement might look like. This provides the overall rationale for the study we present here.

The intervention we test here targets homocysteine reduction. Homocysteine concentrations amongst women of reproductive age are higher in the Gambian dry season than the rainy season, corresponding with lower concentrations of key one-carbon metabolites such as folate and betaine [6]. In turn, homocysteine is strongly inversely associated with the ratio of S-adenosyl methionine (SAM) to S-adenosyl homocysteine (SAH) [6], a measure of methylation potential [7]. Homocysteine can be hydrolysed to SAH, which acts as an allosteric inhibitor of the SAM to SAH (transmethylation) reaction [8,9]. In our previous research, periconceptional plasma homocysteine concentrations have been strongly inversely associated with infant DNA methylation at several putative metastable epialleles [10–12], genomic loci where methylation is established in the very early embryo [10,13,14]. There is preliminary evidence, for example, that compared with children conceived in the rainy season, those conceived in the dry season demonstrate methylation patterns suggestive of a loss of regular imprinting at the noncoding Vault RNA 2–1 (*VTRNA2-1*) [12], a locus implicated in immune function and tumour suppression [15,16]. This suggests that infant DNA methylation could be optimised by improving maternal methylation potential in the Gambian dry season through the reduction of homocysteine concentrations.

The effect of a homocysteine-lowering nutritional intervention on infant DNA methylation would need to be tested in a Phase III preconception trial. However, an important first step is to identify the most effective candidate nutritional supplement. We therefore conducted a randomised controlled trial in nonpregnant women, with homocysteine reduction as the primary outcome. We designed a novel drink powder containing betaine and B vitamins tailored specifically to the target population. Recognising that there are also existing, well-accepted multiple micronutrient supplements containing similar ingredients, we tested the drink powder against the United Nations Multiple Micronutrient Preparation (UNIMMAP). Although there are many nutritional intervention studies investigating homocysteine reduction, our study rationale comprised several context-specific questions. First, could a nutritional supplement lower homocysteine in a population where hyperhomocysteinemia is not a known problem? Second, could we specifically reduce Gambian dry season homocysteine concentrations to those found in the rainy season? Third, would a bespoke nutritional supplement have any advantage over a supplement that is already available? By answering these questions, we aimed to identify the most effective candidate nutritional supplement to carry forwards to preconception trials assessing the impact of maternal nutritional status on offspring DNA methylation.

## Methods

### Trial outline

We conducted a three-arm, parallel, unblinded, randomised, controlled trial with 298 women. Nonpregnant, non-lactating, healthy women of reproductive age from the West Kiang region of The Gambia were randomised in a 1:1:1 allocation ratio to 12 weeks of daily supplementation of either (a) a novel betaine and B vitamins drink powder supplement (‘drink powder’), (b) an existing available micronutrient tablet (UNIMMAP), or (c) no intervention (control). All participants were invited for midline (week 5) and endline (week 12) visits for anthropometric measurements and to provide a venepuncture sample. The trial was registered at Clinicaltrials.gov (NCT03431597). The full trial protocol is available in S1 Appendix. The supplementation phase of the trial took place from March to June 2018, in the Gambian dry season.

Our primary objective was to assess the effect of the drink powder on plasma homocysteine after 12 weeks of daily supplementation versus the control group. Our secondary objectives were to look at this comparison at midline, to compare the efficacy of the drink powder versus the UNIMMAP tablet at midline and endline, and to assess any impact of the interventions on blood pressure and pulse versus control at both time points.

### Participants

Study participants came from 21 villages of the West Kiang region. A list of potentially eligible women within West Kiang was generated using the Medical Research Council The Gambia at the London School of Hygiene and Tropical Medicine (MRCG) Keneba Health and Demographic Surveillance System database [17]. At the screening stage a team of 10 fieldworkers visited women at their homes to explain the study and eligibility criteria, and to invite them to join the study by providing informed consent.

Women were eligible at the screening stage if they were premenopausal, aged 18–45 years, nonpregnant to their knowledge, non-lactating (at least 9 months postpartum), had no plan to conceive in the ensuing 3 months, had no plans to travel, and with no current illness or chronic health problems (cardiovascular disease, renal disease, thyroid disease, or cancer). Women were excluded if they were currently taking B vitamin or multivitamin supplements or taking medication for the prevention of seizures (e.g., carbamazepine). Women with high blood pressure were excluded only when there was an additional history of stroke or heart attack.

Women of reproductive age in West Kiang region are deficient in several micronutrients central to one-carbon metabolism, and these have been described in detail in previous studies [6,10,18]. For example, almost all women are deficient in B2, over 40% have low vitamin B6 status, approximately 30% have a low concentration of betaine, and 13% are folate deficient [18].

### Sample size

Unpublished year-round homocysteine data from a recent trial in the same region [19] indicated that women have a mean (standard deviation [SD]) plasma homocysteine concentration of 8.06 μmol/L (±2.5) in the peak dry season of February to April. We wanted to detect a minimum change in plasma homocysteine of 1 μmol/L, which would reduce it to below the mean rainy season concentration (7.31 μmol/L). This required 99 women per arm to give 80% power (at 5% significance level using a two-sided *t* test), which we increased to 125 to allow for 20% attrition.

### Study design

Women who provided informed consent at the screening stage were invited to come for the baseline visit at MRCG Keneba field station. They provided a urine sample for the study nurses to conduct a rapid pregnancy test and, if negative, were formally enrolled onto the trial.

#### Randomisation

Study participants were randomised to the drink powder, UNIMMAP tablet, or control arm according to a computer-generated randomisation scheme. A list of study IDs was generated in advance by the Keneba database manager. Prior to the baseline visit, the statistician assigned each participant’s study ID to one of the three trial arms (‘drink powder’, ‘tablet’, ‘control’) in a 1:1:1 ratio using block randomisation (with a block size of 15). The sub-investigator printed the study ID and group allocation onto a piece of paper and sealed it in an envelope. The envelopes were arranged in consecutive order of participants 1 to 375, and kept for the baseline visit. The sealed envelopes were not an attempt to keep the group allocation blind, but helped ensure that only one person received a study ID and corresponding group allocation at a time upon enrolment, overseen by the field coordinator.

#### Study visits

At the baseline visit, all enrolled women had their weight (Seca scale model 803, ±0.1 kg), height (Seca stadiometer model 217 ± 0.1 cm), and blood pressure (Omron M2 automatic monitor) measured. Each measurement was taken in triplicate and the average used. The study nurses collected a fasted venepuncture sample from the participants into 9-mL EDTA monovettes, which were kept on ice. The samples were processed by the laboratory in Keneba within one hour of collection. Samples were spun using an Eppendorf 5810R centrifuge at 1,800 rpm for 10 minutes at 4 °C to separate the plasma. Plasma aliquots were then immediately stored at −70 °C until analysis.

Participants were invited back for a midline visit at week 5 and an endline visit at week 12. The midline visit was programmed at week 5 rather than week 6 to ensure that samples were collected before the start of Ramadan. The exact same procedures were repeated as for baseline. If women were pregnant at midline, they were given the choice of whether to continue in the study or not. Although supplements were safe for pregnancy, the information was recorded to be able to account for any effects of hemodilution at the analysis stage. There were no changes to the originally planned methods after trial commencement.

#### Design of the novel betaine and B vitamins drink powder

To tailor the supplement to our target population, we analysed three separate data sets containing plasma nutrient concentrations from women of reproductive age in West Kiang. The plasma nutrients were comprised of metabolites having the potential to reduce homocysteine through one-carbon metabolic pathways, many of which having successfully reduced homocysteine in previous intervention studies [20–22]. We ran multivariable linear regression models to assess which nutrients independently predicted plasma homocysteine. We selected those that consistently demonstrated an inverse relationship with homocysteine (folate, B12, B2, betaine) as candidates for the supplement. Full details of these analyses, together with a more detailed discussion of homocysteine metabolism, can be found in S2 Appendix. The chosen supplement ingredients were then carefully assessed against existing dosage safety information, doses given in previous trials, and current plasma nutrient levels to establish final supplement doses well below upper safety limits. Full details of this process are provided in S3 Appendix.

#### Supplement composition and supplementation schedule

Table 1 details the composition of the two intervention products. The drink powder contained 4 g of anhydrous betaine with two times the dietary reference intake (DRI) of folic acid, vitamin B12, and vitamin B2 (manufactured by Kendy Ltd., Bankya, Bulgaria, sourcing betaine from Danisco, Naantali, Finland). The UNIMMAP tablet contained 15 micronutrients at the DRI (manufactured by DSM Nutritional Products, Isando, South Africa).

**Table 1:** Composition of interventional products, per daily dose.

Novel drink powder		UNIMMAP tablet	
Ingredient	Amount in supplement	Ingredient	Amount in supplement
Folate as folic acid	800 μg	Folic acid	400 μg
Vitamin B12 as cyanocobalamin	5.2 μg	Vitamin B12	2.6 μg
Riboflavin (vitamin B2) as Riboflavin-5′-phosphate	2.8 mg	Riboflavin	1.4 mg
Betaine as anhydrous betaine	4 g	-	-
-	-	Vitamin A	800 μg RE
-	-	Vitamin D	5 μg
-	-	Vitamin E	10 mg
-	-	Thiamine	1.4 mg
-	-	Niacin	18 mg
-	-	Vitamin B6	1.9 mg
-	-	Vitamin C	70 mg
-	-	Zinc	15 mg
-	-	Iron	30 mg
-	-	Iodine	150 μg
-	-	Selenium	65 μg
-	-	Copper	2 mg

Abbreviations: RE, retinol equivalent; UNIMMAP, Daily United Nations Multiple Micronutrient Preparation.

Those participants who were randomised to the intervention arms at baseline started their supplementation the next day in their home villages. The supplements were supplied to participants on a daily basis by village assistants (VAs). VAs were employees of MRCG living in the villages they were supervising. The participants visited their VA on a daily basis. The VAs dissolved the drink powder into 200 mL of water, and participants were given the option to add sugar to taste in case it was too bitter. UNIMMAP was provided in capsule form, to be taken with water. The VAs observed consumption of the supplements. The intensive, daily monitored supplementation schedule meant we termed this trial an efficacy trial.

Once a week, field assistants delivered weekly packs of supplements to the VAs to administer to the participants in their village. The weekly pack included a tally sheet for the VA to record any adverse events (AEs) experienced by the participants daily. They also included seven small coloured cards that were given to the participants, who were asked to give in a card every time they visited the VA in order to measure compliance. Each week the field assistants would compile a report on the AEs from the tally sheets and record compliance, measured by counting the number of cards a participant had returned that week and cross-checking with the number of remaining supplements in the pack. After the 12 weeks of supplementation, the participants continued to be monitored for a further 3 weeks to capture any delayed AEs.

### Laboratory methods

We analysed the concentration of plasma homocysteine in samples from baseline, midline, and endline visits at the MRCG Keneba laboratory using the COBAS INTEGRA^®^ 400 Plus analyzer. All samples were analysed in one batch on completion of the endline visit. All samples experienced one cycle of freeze-thaw. Inter-assay coefficients of variation (CVs) were <4.0%, and intra-assay CVs were <5.1%.

### Blinding

Due to the different characteristics of the drink powder and the tablet, the participants, field assistants, and VAs were not blinded to group allocation. The laboratory technician analysing the plasma homocysteine was blinded to group allocation.

### Ethical considerations

The trial received ethics approval from The Gambia Government/MRC Joint Ethics Committee (reference SCC 1575v1.2). All participants provided informed consent by signature, if literate, and otherwise by thumb print in the presence of an impartial witness, if illiterate. The MRCG Clinical Trials Support Office conducted four monitoring trips to ensure the trial was implemented according to the international standards of Good Clinical Practice. All procedures were in accordance with the Helsinki Declaration of 1975 as revised in 1983.

### Statistical analysis

Compliance was calculated as a percentage of the total number of cards received for each participant over the trial duration. We summarised AEs by calculating the proportion of participants that had self-reported any AE at any point over the trial duration. We compared the baseline distribution of continuous variables between the three trial arms using the Kruskal-Wallis test. We used the Wilcoxon rank-sum test to compare medians of percentage compliance between the two intervention arms, and the chi-squared/Fisher exact test for proportions of participants reporting morbidities.

We used linear regression models to assess the efficacy of the supplements at lowering homocysteine at midline and endline. We first assessed the homocysteine variables for normality using the Shapiro-Wilk test. Variables were right skewed but satisfied normality assumptions after log transformation. We then fitted linear regression models comparing the mean difference in log homocysteine between pairs of trial arms at midline or endline, adjusted for baseline homocysteine, age, and body mass index (BMI). The β coefficients from the regression models provided an estimate of the log ratio of geometric mean homocysteine concentrations, which we also chose to present as percentage change in the geometric mean using the formula (e^β^-1)*100. The primary analyses were based on a complete case analysis. We also performed a sensitivity analysis with data restricted to nonpregnant, fasted, compliant (>80% compliance) individuals. All analyses were performed using Stata 15.1 (StataCorp 2017, TX).

## Results

**Fig 1:**
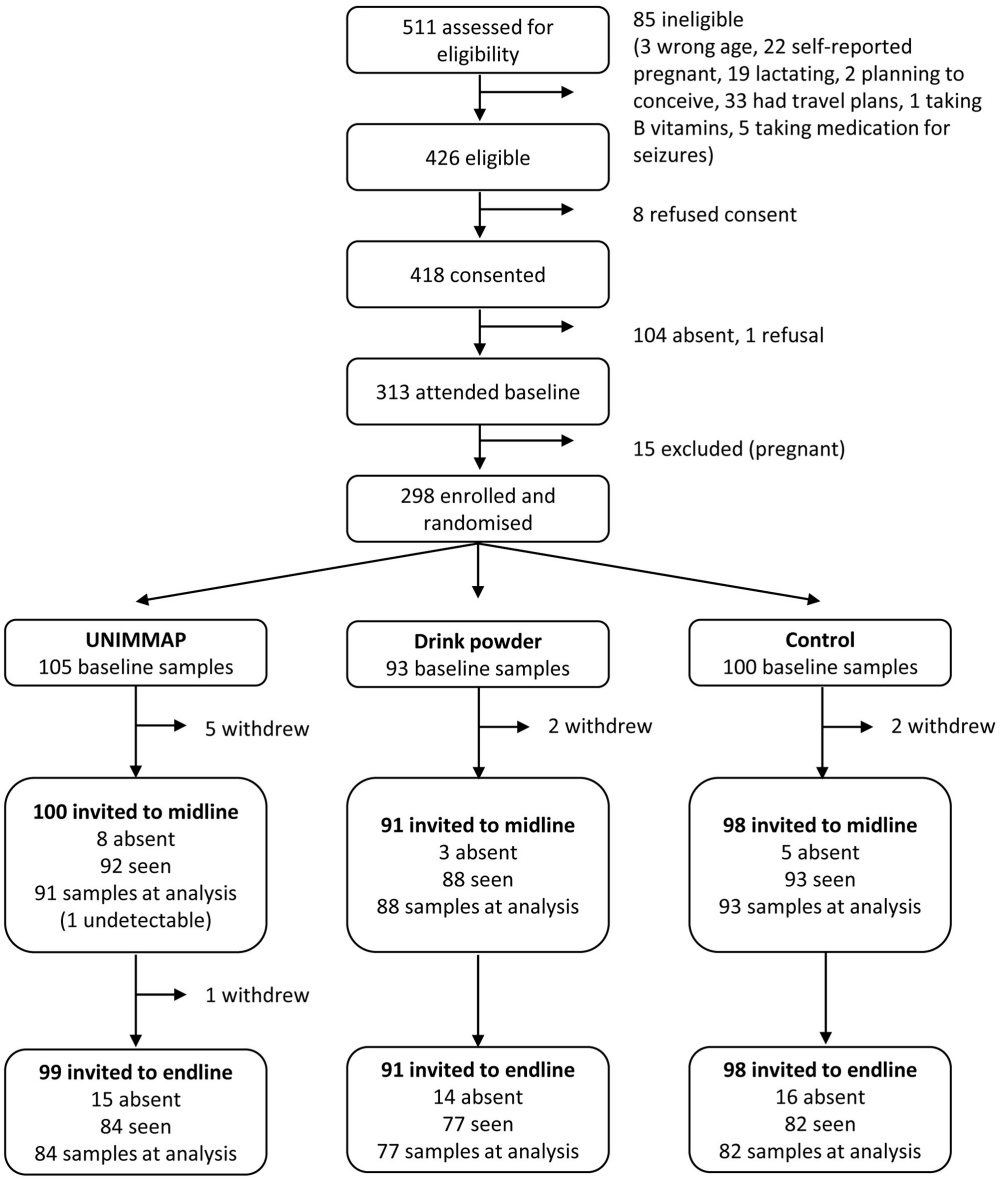
Flow diagram of trial participants. UNIMMAP, Daily United Nations Multiple Micronutrient Preparation.

The participant flow diagram is shown in Fig 1. Of the 511 women initially approached for the trial, 100 were ineligible, 9 refused to start the study, and 104 did not attend the baseline visit. A total of 298 eligible women were therefore enrolled and randomised to the drink powder, UNIMMAP, and control arms. Although only 10 women officially withdrew over the study time frame (9 before midline and 1 before endline), on the days of the study visits, there were many absentees. At midline there were 16 absentees and 45 at endline, despite those in the supplement arms continuing with the intervention in their villages. There were 298, 273, and 243 plasma samples available for homocysteine analysis at baseline, midline, and endline, respectively. Of these, one (in the UNIMMAP arm at midline) had a homocysteine reading below the detection limit.

Baseline characteristics for the enrolled participants are provided in Table 2. There was no difference in distribution of age, BMI, homocysteine concentration, blood pressure, and pulse of participants between trial arms. There was no difference in any of these baseline parameters between those present and absent at midline and endline follow-up.

**Table 2:** Baseline characteristics.

Variable	Control (*N* = 100); median (IQR)	Drink powder (*N* = 93); median (IQR)	UNIMMAP (*N* = 105); median (IQR)	*p*-Value*
Age (years)	28.5 (22.1–38.7)	30.7 (21.7–39.7)	26 (20.2–38.8)	0.57
BMI (kg/m^2^)	21.3 (18.6–25.3)	21.9 (19.6–26.3)	21.0 (19.0–23.5)	0.81
Homocysteine (μmol/L)	9.7 (7.9–12.3)	10.4 (7.6–14.1)	10.2 (7.2–12.7)	0.27
Systolic BP (mmHg)	114 (105–124)	114 (107–122)	114 (105–122)	0.30
Diastolic BP (mmHg)	70 (63–77)	70 (65–77)	69 (62–75)	0.30
Pulse (beats per minute)	72 (65–79)	71 (63–81)	72 (66–77)	0.96

*Kruskal-Wallis test.

Abbreviations: BMI, body mass index; BP, blood pressure; IQR, interquartile range; UNIMMAP, Daily United Nations Multiple Micronutrient Preparation.

**Fig 2:**
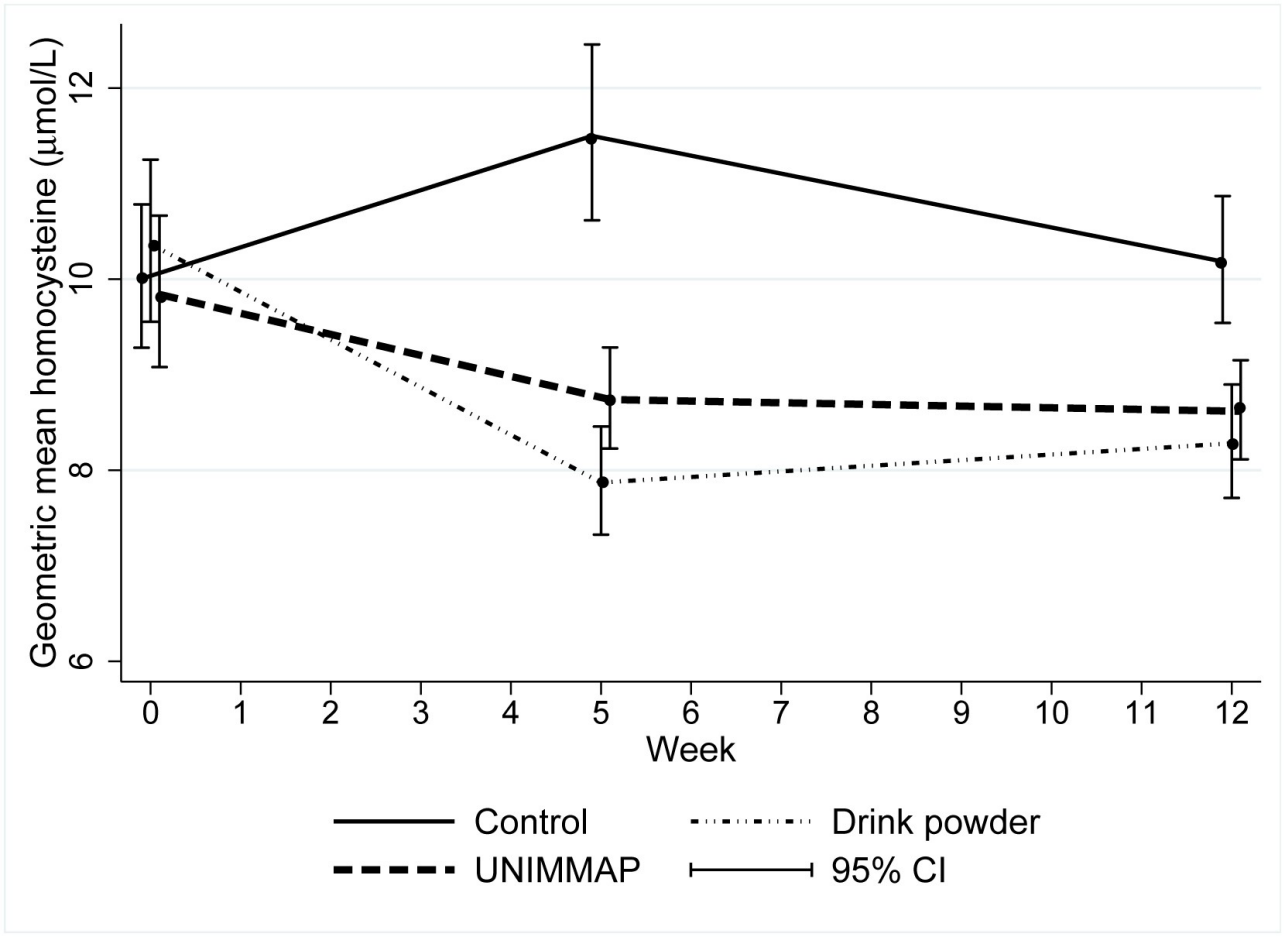
Geometric mean homocysteine over time by trial arm. Error bars = 95% CIs. CI, confidence interval; UNIMMAP, Daily United Nations Multiple Micronutrient Preparation.

The unadjusted homocysteine concentrations at each time point are shown in Table 3 and Fig 2. In the drink powder arm, the geometric mean homocysteine concentration decreased by 2.67 μmol/L and 2.32 μmol/L compared with the baseline at midline and endline, respectively. A similar, though attenuated, pattern was observed in the UNIMMAP arm, in which midline and endline mean homocysteine concentrations were 1.28 μmol/L and 1.22 μmol/L lower than baseline, respectively.

**Table 3:** Unadjusted plasma homocysteine (μmol/L) by time point.

Study arm	Baseline		Midline		Endline	
	*N*	Geometric mean (95% CI)	*N*	Geometric mean (95% CI)	*N*	Geometric mean (95% CI)
Control	100	10.00 (9.28–10.78)	93	11.50 (10.62–12.46)	82	10.18 (9.54–10.87)
UNIMMAP	105	9.83 (9.10–10.62)	91	8.55 (8.02–9.12)	84	8.61 (8.14–9.12)
Drink powder	93	10.59 (9.67–11.60)	88	7.90 (7.33–8.51)	77	8.27 (7.66–8.92)

Abbreviations: CI, confidence interval; UNIMMAP, Daily United Nations Multiple Micronutrient Preparation.

Table 4 provides the results of the pairwise comparisons between trial arms at endline and midline, adjusted for age, BMI, and baseline homocysteine. Compared with the controls, the drink powder and UNIMMAP reduced mean plasma homocysteine by 23.6% (−29.5 to −17.1) and 15.5% (−21.2 to −9.4), respectively (both *p* < 0.001). Compared with UNIMMAP, the drink powder reduced the mean homocysteine by 8.8% (−15.8 to −1.2; *p* = 0.025). Effects at midline were more pronounced, in which the drink powder reduced mean plasma homocysteine by 35.1% (−39.7 to −30.2) compared with the controls, and UNIMMAP reduced homocysteine by 23.1% (−28.1 to −17.8). The drink powder reduced mean homocysteine 14.3% (−20.0 to −8.2) more than UNIMMAP (all *p*-values <0.001).

**Table 4:** Multivariable regression results showing percentage difference in geometric mean plasma homocysteine between trial arms.

Trial arms	Time point	*N*	β (95% CI)*	Percent difference in geometric means**	*p*-Value
Drink powder versus control^Ɨ^	12 weeks	159	−0.27 (−0.35 to −0.19)	−23.6 (−29.5 to −17.1)	<0.001
UNIMMAP versus control	12 weeks	166	−0.17 (−0.24 to −0.10)	−15.5 (−21.2 to −9.4)	<0.001
Drink powder versus UNIMMAP	12 weeks	161	−0.09 (−0.17 to −0.01)	−8.8 (−15.8 to −1.2)	0.025
Drink powder versus control	5 weeks	181	−0.43 (−0.51 to −0.36)	−35.1 (−39.7 to −30.2)	<0.001
UNIMMAP versus control	5 weeks	184	−0.26 (−0.33 to −0.20)	−23.1 (−28.1 to −17.8)	<0.001
Drink powder versus UNIMMAP	5 weeks	179	−0.15 (−0.22 to −0.09)	−14.3 (−20.0 to −8.2)	<0.001

*β represents a difference in log homocysteine at a time point between trial arms, adjusted for baseline homocysteine, age, and BMI at the time point.

**Calculated from (e^β^-1)*100.

^Ɨ^Primary outcome.

Abbreviations: BMI, body mass index; CI, confidence interval; UNIMMAP, Daily United Nations Multiple Micronutrient Preparation.

We performed a sensitivity analysis excluding any participants who became pregnant during the study period, who gave a non-fasted blood sample, or had compliance <80%. At midline, 8 participants were found to be pregnant and decided to continue with the study (2/93 [2.15%] in the control arm, 0 in the drink powder arm, and 6/92 [6.52%] in the UNIMMAP arm). At endline, a total of 19 women were pregnant (4/82 [4.88%] in the control arm, 7/77 [9.09%] in the drink powder arm, and 8/84 [9.53%] in the UNIMMAP arm). At midline, one person reported they had not fasted before venepuncture (in the UNIMMAP arm). At endline, 16 participants had not fasted (1 in control arm, 6 in drink powder arm, 9 in UNIMMAP arm). The trial results excluding pregnant, non-fasted, and noncompliant individuals are provided in S1 Table. Effect sizes and *p*-values are similar to those described in the complete case analysis above.

Blood pressure and pulse results are provided in Table 5 and S2 Table. Overall effect sizes were very small, and there was no evidence to suggest that either intervention decreased blood pressure or pulse compared with the controls at endline.

**Table 5:** Multivariable linear regression results showing difference in blood pressure and pulse between trial arms.

**Systolic blood pressure**				
**Trial arms**	**Time point**	***N***	β (95% CI)*	***p*-Value**
Drink powder versus control	12 weeks	159	−0.01 (−0.04 to 0.01)	0.233
UNIMMAP versus control	12 weeks	165	0.01 (−0.01 to 0.04)	0.213
Drink powder versus UNIMMAP	12 weeks	160	−0.03 (−0.05 to −0.01)	0.008
Drink powder versus control	5 weeks	181	0.01 (−0.01 to 0.03)	0.511
UNIMMAP versus control	5 weeks	184	0.02 (0.00 to 0.04)	0.024
Drink powder versus UNIMMAP	5 weeks	179	−0.02 (−0.04 to 0.00)	0.104
**Diastolic blood pressure**				
**Trial arms**	**Time point**	***N***	β (95% CI)*	***p*-Value**
Drink powder versus control	12 weeks	159	−0.01 (−0.04 to 0.01)	0.329
UNIMMAP versus control	12 weeks	165	0.00 (−0.02 to 0.03)	0.746
Drink powder versus UNIMMAP	12 weeks	160	−0.02 (−0.04 to 0.01)	0.129
Drink powder versus control	5 weeks	181	−0.01 (−0.03 to 0.01)	0.318
UNIMMAP versus control	5 weeks	184	0.00 (−0.02 to 0.02)	0.967
Drink powder versus UNIMMAP	5 weeks	179	−0.01 (−0.03 to 0.01)	0.290
**Pulse**				
**Trial arms**	**Time point**	***N***	β (95% CI)*	***p*-Value**
Drink powder versus control	12 weeks	159	−0.02 (−0.05 to 0.01)	0.218
UNIMMAP versus control	12 weeks	165	0.00 (−0.04 to 0.03)	0.780
Drink powder versus UNIMMAP	12 weeks	160	−0.02 (−0.05 to 0.02)	0.368
Drink powder versus control	5 weeks	181	0.00 (−0.04 to 0.03)	0.695
UNIMMAP versus control	5 weeks	184	−0.02 (−0.05 to 0.01)	0.222
Drink powder versus UNIMMAP	5 weeks	179	0.01 (−0.02 to 0.04)	0.510

*β represents the difference in the log-transformed dependent variable between trial arms at each time point, adjusted for the baseline value of the dependent variable, age, and BMI at the time point.

Abbreviations: BMI, body mass index; CI, confidence interval; UNIMMAP, United Nations Multiple Micronutrient Preparation.

In this trial, supplementation was observed daily, and compliance was extremely high in both intervention arms, at 97.8% for UNIMMAP and 98.8% for the drink powder (Table 6). There was no difference in self-reported AEs between the intervention arms. Table 5 describes the proportion of participants who reported an AE to their VA on at least one day over the 12-week period. The most common AEs described for the two interventions were abdominal pain, nausea, and dizziness, followed by urine discolouration and fever. The frequency of these AEs, however, was low. Mean AE duration across the trial was less than half a day per person, and the maximum number of days an individual reported a given AE was 19 days, for abdominal pain (additional details in S3 Table). There were two serious AEs reported over the trial duration (one miscarriage in week 9 and one stroke in week 10), both in the drink powder arm. The Trial Safety Monitor and Medical Expert judged both events as highly unlikely to have been caused by the supplement, and both participants resumed participation in the trial after the events.

**Table 6:** Compliance and self-reported morbidity (condition ever experienced over trial).

Compliance and morbidity	UNIMMAP	Drink powder	*p*-Value*
**Percent compliance** (Median IQR)	97.8 (91.0–100.0)	98.8 (91.6–100.0)	0.23
**Nausea** (*N*%)	17/104 (16.4)	8/93 (8.6)	0.10
**Dizziness** (*N*%)	14/104 (13.5)	12/93 (12.9)	0.91
**Urine discolouration** (*N*%)	4/104 (3.9)	8/93 (8.6)	0.23
**Abdominal pain** (*N*%)	19/104 (18.3)	13/93 (14.0)	0.41
**Fever** (*N*%)	4/104 (3.9)	6/93 (6.5)	0.52

*Wilcoxon rank-sum test of medians for percentage compliance, chi-squared test/Fisher exact test for morbidity.

Abbreviations: IQR, interquartile range; UNIMMAP, Daily United Nations Multiple Micronutrient Preparation supplement.

## Discussion

In this trial, both supplements were highly efficacious in reducing homocysteine after 12 weeks of daily supplementation, with the greatest effect sizes apparent after 5 weeks. There was evidence to suggest the drink powder was more efficacious than UNIMMAP at midline and that this advantage was maintained, although diminished, at endline. Trial compliance was high and self-reported AEs were low throughout the trial, and neither varied by intervention arm. The trial confirms an important proof of principle that dietary supplements can influence metabolic pathways that we have shown in previous observational studies to predict offspring methylation levels [10–12].

Nutritional supplements comprising various combinations of folic acid, B vitamins, and methyl donors such as betaine have been successful in lowering plasma homocysteine in multiple trials [20,23–26]. However, despite a call over 10 years ago for more supplementation studies looking at the combined effect of B vitamins with betaine [27], there still remain very few. A meta-analysis of 12 folic acid supplementation trials (with or without additional B vitamins) showed that folic acid supplementation between 1 and 3 months of supplementation reduced homocysteine by 25% (similar across all doses from 0.5 to 5 mg) [20]. The UNIMMAP multivitamin tablet, which contains one DRI of folic acid, B12, B2, and B6, amongst other ingredients, demonstrated similar efficacy in our trial.

The drink powder contained double the amount of folic acid, B12, and B2 compared with UNIMMAP, alongside the addition of betaine, components that may explain its comparatively greater efficacy. Doubling the B vitamin dosage may not greatly contribute towards enhanced homocysteine-reduction properties: previous studies suggest that the dose of folic acid does not greatly matter [20], that additional B12 also has a disputed effect [20,28], and that B2 supplementation is only effective amongst those with the rare methylenetetrahydrofolate reductase (*MTHFR*) 677 TT genotype [29,30]. In contrast, in a meta-analysis, 4–6 g of daily betaine supplementation for 6–12 weeks decreased plasma homocysteine by 1.23 μmol/L, corresponding to a 11.8% reduction of mean baseline values [22]. We can therefore speculate that the betaine in the drink powder has an additive effect supplementary to the action of folic acid and B vitamins in UNIMMAP. The results also suggest that the vitamin B6 in UNIMMAP is not required to reduce homocysteine in this population, supporting findings from previous trials, including those involving participants with low B6 status [20,31,32]. It appears that tailoring the drink powder design to the nutritional profile of the local population using plasma determinants of homocysteine worked well, and could be a viable approach for future supplement design.

The performance of both interventions surpassed our expectations. Given that many of the previous folic acid supplementation trials have been performed in patients with hyperhomocysteinemia, those with increased risk of cardiovascular events, and amongst more elderly participants [20], we had envisaged a more modest reduction of around 1 μmol/L in our healthy, relatively young population. Furthermore, because we could have expected plasma homocysteine to decrease in the controls by endline in mid-June (expected seasonal trend shown in Fig 1 in S1 Appendix), we did not anticipate such a strong effect beyond the seasonal trend. Interestingly, plasma homocysteine concentrations in the controls did not decrease over the trial period. Indeed, the heightened efficacy of the interventions at midline may be driven by higher plasma homocysteine at this time in the controls. A previous longitudinal study of one-carbon biomarkers in nonpregnant women in The Gambia also showed that maximum plasma homocysteine concentration and minimum folate concentration occurred in late March–early April [6], the timing of our midline.

Although the drink powder reduced homocysteine more quickly than UNIMMAP at midline, by endline the differences were less pronounced. We have a particular interest in the potential for a micronutrient supplement to influence the establishment of DNA methylation marks in the very early embryo [10,14]. The sensitive periconceptional period may extend back beyond the three months required for the most active phase of oocyte maturation [2], and there is increasing awareness that interventions should cover longer periods than this to account for unknown timing of conception [33]. Therefore, factors such as cost-effectiveness and long-term acceptability become relevant considerations for candidate supplements alongside speed of action. The high compliance rates in both arms suggest that both interventions were acceptable in the village community settings. The low reported morbidity also reinforces the low-risk nature of these supplements. UNIMMAP has been provided in preconception and pregnancy trials in numerous settings [34–42]. Of the trials that reported morbidity, the majority found there to be no difference between UNIMMAP and control arms. Given that the drink powder arm in our trial reported similar levels of AEs to the UNIMMAP arm, we have assurance that both are well tolerated, further supported by the fact that no participant withdrew from the trial on account of AEs.

Lowering homocysteine and providing one-carbon-related nutrients are essential for a range of biological processes related to fertility and embryo development, and epigenetic mechanisms are just one of several aspects to consider at periconception [2,43,44]. Future supplement designs may therefore want to consider adding other nutrients essential for optimal fetal development that were beyond the scope of this trial. Choline, for example, not only acts as a metabolic precursor to betaine but as an essential component of lipids, lipoproteins, and neurotransmitters [45,46]. However, the interrelated nature of metabolic pathways may mean that the provision of supplemental folic acid and betaine is sufficient to reduce the demand for choline for transmethylation and therefore spare it for other important functions [47–49]. Further research is required to determine the extent of such compensatory mechanisms and the implication for supplement design.

Our trial had some limitations. We used homocysteine as an integrated indicator of B vitamin status and methylation potential, but, had resources allowed, we would have liked to quantify the effects of the two supplements on micronutrient status. The study visits had a number of no-shows. However, absentees at midline and endline were not different in baseline characteristics from those who attended. Unfortunately, we were unable to extend the midline visit due to the need to avoid the month of Ramadan, when venepuncture sample collection was not viable. Whilst this meant we did not achieve our intended sample size of 99 women per arm, we were adequately powered to detect the larger reduction in homocysteine observed in our sample. Resource limitations restricted the number of time points we could analyse, but future trials may benefit from considering additional time points and exploring potential nonlinear patterns of homocysteine lowering over time. Conducting this trial earlier in the dry season, for example January to March, when plasma homocysteine concentrations would be expected to be higher than our implementation period of March to June, may have enabled us to see an even stronger impact of the interventions. Practical limitations also meant we did not visit the control group on a daily basis as we did for the intervention groups. Therefore, we did not collect morbidity information from controls, which would have helped us assess the AEs from the interventions relative to the general population. Finally, we did not collect information on genetic polymorphisms that may have affected homocysteine metabolism [50].

In conclusion, both supplements worked to reduce homocysteine concentrations from those seen in the dry season to below those in the rainy season, and therefore met the trial objective. It is premature to speculate whether the improvements on homocysteine reduction offered by the drink powder would lead to significant changes in offspring DNA methylation in future trials. As such, they both remain potential candidates for future epigenetic trials in pregnancy in the rural Gambian setting. This is contingent on future research confirming causal links between maternal nutrient exposure, offspring methylation, and phenotypic effects, which is an area of continued investigation.

## Supporting information

S1 Appendix Clinical trial protocol. (PDF)

S2 Appendix Determinants of plasma homocysteine as ingredients for the novel drink powder supplement. (PDF)

S3 Appendix Safety and dosage considerations for the drink powder supplement. (PDF)

S1 Table Multivariable regression results showing percentage difference in geometric mean plasma homocysteine between trial arms, restricted to fasted, nonpregnant, compliant participants. (PDF)

S2 Table Unadjusted blood pressure and pulse results by time point and trial arm. (PDF)

S3 Table Additional details on morbidity. (PDF)

S1 Data Data underlying the findings. (XLS)

S1 CONSORT Checklist CONSORT checklist. (PDF)

## Abbreviations

AE: adverse event
BMI: body mass index
CI: confidence interval
CV: coefficient of variation
DRI: dietary reference intake
IQR: interquartile range
MRCG: Medical Research Council The Gambia at the London School of Hygiene and Tropical Medicine
MTHFR: methylenetetrahydrofolate reductase
RE: retinol equivalent
SAH: S-adenosyl homocysteine
SAM: S-adenosyl methionine
SD: standard deviation
UNIMMAP: United Nations Multiple Micronutrient Preparation
VA: village assistant
VTRNA2-1: Vault RNA 2–1
