# Supplementary material for: A novel nutritional supplement to reduce plasma homocysteine in nonpregnant women: A randomised controlled trial in The Gambia
Source: PLoS Med. 2019 Aug 13;16(8):e1002870. doi: 10.1371/journal.pmed.1002870 (PMC6691988; doi:10.1371/journal.pmed.1002870)
Supplement: S1 Appendix — (PDF) [file pmed.1002870.s001.pdf]

CLINICAL TRIAL PROTOCOL

Effectiveness of a micronutrient supplement to lower plasma homocysteine in non-pregnant women of reproductive age (Methyl Donors & Epigenetics 2 [MDEG2] Pilot Supplementation Trial)

**SCC No:**	1575
**Alias**	MDEG2 Pilot Supplementation Trial
**Other Number(s)**	
**Protocol Version – Date**	Version 4.1 May 14th 2018
**Sponsor**	Patricia Henley<br>Quality & Governance Manager<br>London School of Hygiene & Tropical Medicine<br>Keppel St, London, WC1E 7HT<br>UK
**Principal Investigator**	Prof Andrew M Prentice

Signature page

The clinical trial will be carried out in accordance with the protocol, the ICH Harmonised Tripartite Guideline for Good Clinical Practice, <<*insert other regulations if applicable*>>, and in accordance to local legal and regulatory requirements.

Principal Investigator:	Signature:	Date:
Prof Andrew Prentice	A M Prentice	14.05.2018
Name		

Sponsor's representative:	Signature:	Date:
JONAS LEXOW	[illegible]	12 JULY 2018
Name		

Table of contents

Page

Signature page 2
Key roles 5
List of abbreviations 7
Protocol summary 8
1 Background information and rationale 10
1.1 Background information 10
1.2 Rationale 11
1.3 Potential risks and benefits 13
2 Study objectives 13
2.1 Study endpoints 13
3 Study design 14
3.1 Type of study and design 14
3.2 Randomisation and blinding procedures 15
3.2.1 Randomisation 15
3.2.2 Blinding 16
3.3 Sub-studies 16
3.4 Investigational products 16
3.4.1 Description of products 16
3.4.2 Formulation, packaging and labelling 16
3.4.3 Product storage and stability 18
3.4.4 Dosage, preparation and administration of investigational products 18
3.4.5 Concomitant medications/treatments 18
4 Selection and withdrawal of participants 18
4.1 Selection of participants 18
4.2 Eligibility of participants 18
4.2.1 Inclusion criteria 19
4.2.2 Exclusion criteria 19
4.3 Withdrawal of participants 19
5 Study procedures and evaluations 19
5.1 Study schedule 20
5.1.1 Screening 20
5.1.2 Enrollment (Baseline) 20
5.1.3 Follow-up 20
5.1.4 Final study visit 21
5.1.5 Early termination visit 21
5.2 Study evaluations 21
5.2.1 Clinical evaluations 21
5.2.2 Laboratory evaluations 21
6 Safety considerations 21
6.1 Methods and timing for assessing, recording, and analysing safety parameters 21
6.1.1 Adverse events 21

6.1.2 Reactogenicity 22
6.1.3 Serious adverse events (SAEs) 22
6.2 Reporting procedures 22
6.3 Safety oversight 23
7 Discontinuation criteria 23
7.1 Participant’s premature termination 23
7.2 Study discontinuation 24
8 Statistical considerations 24
9 Data handling and record keeping 26
9.1 Data management and processing 26
9.2 Source documents and access to source data 26
9.3 Protocol deviations 27
10 Quality control and quality assurance 27
10.1 Study monitoring 28
11 Ethical considerations 28
11.1 General considerations on human subject protection 28
11.1.1 Rationale for participant selection 30
11.1.2 Evaluation of risks and benefits 30
11.2 Informed consent 30
11.3 Participant confidentiality 31
11.4 Future use of stored specimen 31
12 Financing and insurance 31
13 Publication policy 31
14 References 31
Supplements, appendices and other documents 34

Key roles

For questions regarding this protocol, contact Philip James at MRC, International Nutritional Group at the London School of Hygiene & Tropical Medicine, Keppel Street, London, WC1E 7HT, UK. Philip.James@lshtm.ac.uk, +44 7971 263 776.

**Author(s):**	Mr Philip James, Dr Matt J Silver and Prof Andrew M Prentice
**Sponsor's representative:**	Patricia Henley<br>Quality & Governance Manager<br>London School of Hygiene & Tropical Medicine<br>Keppel St, London, WC1E 7HT<br>UK<br>Email: RGIO@lshtm.ac.uk
**Chief Investigator:**	Prof Andrew M Prentice<br>Nutrition Theme Leader<br>MRC Unit The Gambia<br>PO Box 273, Banjul<br>The Gambia<br>E-mail: Andrew.Prentice@lshtm.ac.uk
**Principal Investigator(s):**	Dr Matt J Silver<br>Senior Investigator Scientist<br>MRC Unit The Gambia<br>PO Box 273, Banjul<br>The Gambia<br>E-mail: Matt.Silver@lshtm.ac.uk
**Sub-Investigator(s):**	Philip James<br>Research Assistant / PhD Candidate<br>MRC Unit The Gambia &<br>London School of Hygiene & Tropical Medicine<br>E-mail: Philip.James@lshtm.ac.uk
**MRCG's Medical Expert:**	Dr Fatai Akemokwe<br>Head of Clinical Services<br>MRC Keneba Field Station<br>PO Box 273, Banjul<br>The Gambia<br>Email: fakemokwe@mrc.gm
**Trial monitor(s):**	Clinical Trials Support Office<br>MRC Unit The Gambia<br>PO Box 273, Banjul<br>The Gambia

**Local Safety Monitor:**	Dr Karen Forrest (training up Dr Patrick Nshe for the role)<br>Head of Clinical Services<br>MRC Unit The Gambia<br>PO Box 273, Banjul<br>The Gambia<br>E-mail: k.forrest@mrc.gm
**Chair of DMC/DMSB:**	Overseen by Local Safety Monitor
**Statistician:**	Dr Nuredin Ibrahim Mohammed<br>MRC Unit The Gambia<br>PO Box 273, Banjul<br>The Gambia<br>E-mail: nimohammed@mrc.gm
**External Adviser:**	Dr Sophie Moore, Kings College London<br>Prof Patrick Stover, Cornell University
**Clinical Laboratory/ies:**	MRC Keneba field station laboratory
**Analytical Project Manager**	Ebrima Sise<br>MRC Unit The Gambia<br>PO Box 273, Banjul<br>The Gambia<br>E-mail: ebsise@mrc.gm
**Other institutions/ Collaborators:**	N/A
**Local Ethics Committee**	Gambia Government/MRC Joint Ethics Committee,<br>c/o MRC Unit, The Gambia,<br>PO Box 273, Banjul, The Gambia, West Africa

List of abbreviations

AE	Adverse Event
CpG	Cytosine-phosphate-Guanine
CRF	Case Report Form
DMC	Data Monitoring Committee
GCP	Good Clinical Practice
GRAS	Generally Recognised As Safe
Hcy	Homocysteine
ICH	International Conference on Harmonization
IEC	Independent Ethics Committee
ME	Metastable Epiallele
MRCG	Medical Research Council; represents Medical Research Council Unit The Gambia
PI	Principal Investigator
PLP	Pyridoxal 5’-phosphate
RDA	Recommended Daily Allowance
SAH	S-adenosyl homocysteine
SAM	S-adenosyl methionine
THF	Tetrahydrofolate
UNIMMAP	United Nations Multiple Micronutrient Preparation
VA	Village Assistant

Protocol summary

**Title:**	Effectiveness of a micronutrient supplement to lower plasma homocysteine in non-pregnant women of reproductive age
**Alias :**	MDEG2 Pilot Supplementation Trial
**Phase**:	2
**Population:**	Non-pregnant, non-lactating healthy women of reproductive age in West Kiang
**Number of participants:**	375
**Number of Sites:**	1
**Location of Sites (including satellite sites):**<br>**Trial Duration:**<br>**- Clinical Phase:**<br>**- Whole trial:**	MRCG, Keneba Field Station, The Gambia<br><br>12 weeks clinical phase<br>5 months
**Duration for Participants:**	12 weeks
**Description of Investigational Products:**	a) **Daily novel micronutrient supplement:** 800 µg folic acid, 5.2 µg cyanocobalamin (B12), 2.8 mg Riboflavin-5'-phosphate (B2), 4g trimethylglycine (betaine) in drink powder form<br>**b) Daily United Nations Multiple Micronutrient Preparation (UNIMMAP) supplement:** This includes 15 micronutrients (vitamins A, D, E, B1, B2, B6, B12, C, Niacin, Folic Acid, Fe, Zn, Cu, I, Se) at the Recommended Daily Allowance level, except for folic acid, which is included at a level of 400 µg. Taken as a tablet.<br>c) **Control:** observation only (no placebo)
**Objectives:**	**Primary objective:** To compare the effect of a novel micronutrient supplement taken daily for 12 weeks in the dry season on lowering plasma homocysteine (Hcy) versus the control group.<br><br>**Secondary objectives:**<br><br>• To compare the effect of a novel micronutrient supplement taken daily for 12 weeks versus an existing micronutrient supplement (UNIMMAP) on lowering plasma Hcy.<br>• To compare the effect of a novel micronutrient supplement taken daily for 5 weeks (mid-line) versus control on lowering plasma Hcy.<br>• To compare the effect of a novel micronutrient supplement taken daily for 5 weeks (mid-line) versus UNIMMAP on lowering plasma Hcy.

	• To compare the effect of a novel micronutrient supplement on lowering blood pressure of women at 12 weeks versus the control arm.
**Endpoints:**	**Primary endpoints:** Plasma concentrations of homocysteine after 12 weeks of supplementation.<br><br>**Secondary endpoints:**<br>• Plasma concentrations of homocysteine after 5 weeks of supplementation.<br>• Blood pressure after 12 weeks of supplementation.
**Description of Study Design:**	3-arm randomized controlled trial, unblinded, with 125 women per arm.<br><br>Non-pregnant, non-lactating healthy women of reproductive age in West Kiang will be randomized to 12 weeks of daily supplementation of either a) novel micronutrient supplement, b) existing available micronutrient supplement (UNIMMAP) or c) no intervention (control).<br><br>The supplements will be supplied to participants on a daily basis by Village Assistants(VAs). The VAs will observe consumption of the supplement. The novel micronutrient supplement will be provided in powder form with instructions to dissolve one sachet in a cup of 200ml water. Sugar will be provided to taste. UNIMMAP will be provided in tablet form to be taken with water.<br><br>Women will provide one 10ml fasted venous blood sample at baseline and another after 5 and 12 weeks of supplementation. At each time point they will also have their blood pressure and anthropometry (weight and height) assessed and provide a urine pregnancy test. Baseline socio-demographic information will be captured by questionnaires. Women will be brought to MRCG Keneba for the baseline, 5 week and 12 week data collection visits.<br><br>Throughout the study and for 3 weeks after the intervention adverse events (AEs) will be verbally reported to the VAs. VAs will be provided phones to refer any patients with severe AEs to the MRCG Keneba clinic. Field assistants will be responsible for recording AEs on their weekly supervision rounds.<br><br>Blood samples will be analysed for plasma homocysteine at MRCG Keneba.

1 Background information and rationale

1.1 Background information

Epigenetic processes describe changes to the genome that can alter gene expression without changing the underlying DNA sequence[1]. One such mechanism is DNA methylation of cytosine bases at CpG dinucleotide sites, and there is strong evidence that this can be influenced by a diverse array of intrinsic and environmental factors, including age, disease, stress, exposure to pollutants, and nutrition. Furthermore, epigenetic marks have been associated with a range of diseases affecting health throughout the life course, including cancers, and neurological and metabolic disorders[2]. The epigenome may therefore carry a "cellular memory" of environmental insults, with the potential for lasting effects on health and disease. Epigenetic changes at certain locations are also believed to be heritable, raising the possibility of inter-generational effects that cannot be explained by standard Mendelian genetics[3]. The Developmental Origins of Health and Disease (DOHaD) hypothesis describes the idea that environmental insults experienced early in life can increase the risk of adverse health outcomes throughout the lifecourse. Attention is now shifting to an investigation of the mechanisms that may mediate such observations. In this respect epigenetic modifications to the genome are emerging as a leading candidate that may, at least partially, explain some of the observations described in the DOHaD literature[4,5].

Times of increased cell turnover such as during fetal development and infancy may be particularly susceptible both to epigenetic errors and to environmental influences[6]. The *in utero* period, including periconception, is a period of exceptionally rapid cell differentiation and complex epigenetic remodelling[7]. As such it represents a window in which epigenetic errors could have significant consequences for the health of the child. Of the factors which may impact the infant epigenome *in utero* through maternal exposure, particular attention has been paid to the role of 1-carbon metabolites in the periconceptional period and during embryonic development[8]. 1-carbon metabolism describes the metabolic pathways crucial for the provision of methyl (CH_3) groups required for DNA methylation, and involves the interplay of the folate, methionine, transsulfuration and transmethylation pathways.

Our previous research in The Gambia has explored human diet-epigenome interactions by exploiting a natural experimental design in which fluctuations in energy balance and maternal nutritional exposures display a distinct bimodal seasonal pattern. Almost 20 years ago, we uncovered strong evidence that the season when a child is born has a profound effect on life-long health. Gambian children born during the rainy season are up to 10 times more likely to die prematurely in young adulthood[9]. To explore the extent to which one epigenetic mark, DNA methylation, may mediate some of these observations, Dominguez-Salas *et al*. (2014) compared women conceiving in the peak of the dry and the peak of the rainy season[10]. Maternal periconceptional plasma concentrations of folate, vitamin B2, methionine, betaine and the S-adenosyl Methionine (SAM): S-adenosyl

Homocysteine (SAH) and betaine:dimethyl glycine (DMG) ratios were higher in the rainy season, and concentrations of vitamin B12, homocysteine and SAH were lower. This suggested that the maternal metabolome during the rainy season contained higher concentrations of methyl donors and exhibited a higher methylation potential than the dry season metabolome. Indeed, offspring of these rainy season conceptions had higher levels of CpG methylation at six metastable epialleles (MEs)[1] in peripheral blood monocytes compared to the offspring of those conceived in the dry season, with similar patterns in hair follicle DNA[(10)]. Furthermore concentrations of several methyl donor metabolites in mothers predicted ME methylation in their offspring[(10)]. A subsequent study using the same dataset identified the non-coding RNA gene *VTRNA2-1* as an ME that is sensitive to the periconceptional environment[(11)]. *VTRNA2-1* is an imprinted gene that was found to exhibit decreased methylation, suggestive of a loss of regular imprinting, amongst Gambian offspring conceived in the dry season compared to those conceived in the rainy season. Most recently similar patterns have been found at the *POMC* gene[(12)]. Taken together, the results support the hypothesis that maternal nutrition during the periconceptional period can impact the infant epigenome in humans.

References of literature and data are listed in Section 14.

1.2 Rationale

In rural Gambia women experience considerable variation in their diet by season. The evidence described above suggests women conceiving in the dry season have particular micronutrient deficiencies that result in a lower maternal methylation potential, hindering proper regulation of the fetal epigenome at certain genomic loci. Our long-term goal is to design an intervention that shifts the maternal metabolome to optimise regulation of the infant epigenome by providing micronutrients in the ratio and quantity necessary for optimal 1-carbon metabolism all year round.

To achieve this we first of all require a proof-of-concept trial showing that a nutritional supplement can optimise the metabolome in non-pregnant women by correcting nutritional imbalances and increasing the methylation potential. The primary end point is to reduce plasma homocysteine, since maternal plasma homocysteine is strongly inversely associated with SAM:SAH[(13)], a measure of methylation potential[(14)], and inversely associated with infant methylation at ME loci[(10)]. The hypothesis here is that reducing homocysteine with help improve the methylation potential and enable one-carbon metabolic pathways to function unhindered. Furthermore, there is a wealth of existing evidence to show how nutritional interventions have been successful in reducing homocysteine[(15–17)]. This literature has helped inform the type of ingredients to consider in the supplement, although the final design has been tailored to the West Kiang population (detailed

[1] MEs are genetic loci demonstrating inter-individual differences yet intra-tissue correlation in methylation suggesting exposures impacted methylation prior to gastrulation in the first days after conception[(36)].

below and in Appendix 1). Current investigations into how an improved nutritional profile at conception can better regulate infant methylation patterns and improve infant health are underway in The Gambia (the EMPHASIS study) and elsewhere. There is preliminary evidence suggesting epigenetic dysregulation is involved in poor growth outcomes and cognitive development and possibly also in later-life cardio-metabolic risk factors[18]. Additional benefits of reducing homocysteine are detailed in section 11.1.2.

Our analysis from a sub-sample of women from West Kiang enrolled into the ENID trial (n=350, median 12.1 weeks gestation) in preparation for this trial suggests homocysteine levels (geometric mean) peak in January (9.39 µmol/L) and are at their lowest in June (6.53 µmol/L). The general seasonal trend is shown in Figure 1. Geometric mean (95% CI) homocysteine concentration is higher in the peak dry season (February – April) compared to the peak rainy season (July-September) [8.06 (7.65, 8.50) µmol/L vs. 7.31 (6.82, 7.84) µmol/L; p=0.023]. These results corroborate longitudinal data from previous studies demonstrating similar observations in seasonal homocysteine concentrations[10,13]. The rationale was therefore to design a supplement for this pilot study that could decrease dry season homocysteine plasma concentrations to at least those found in the rainy season, if not lower.

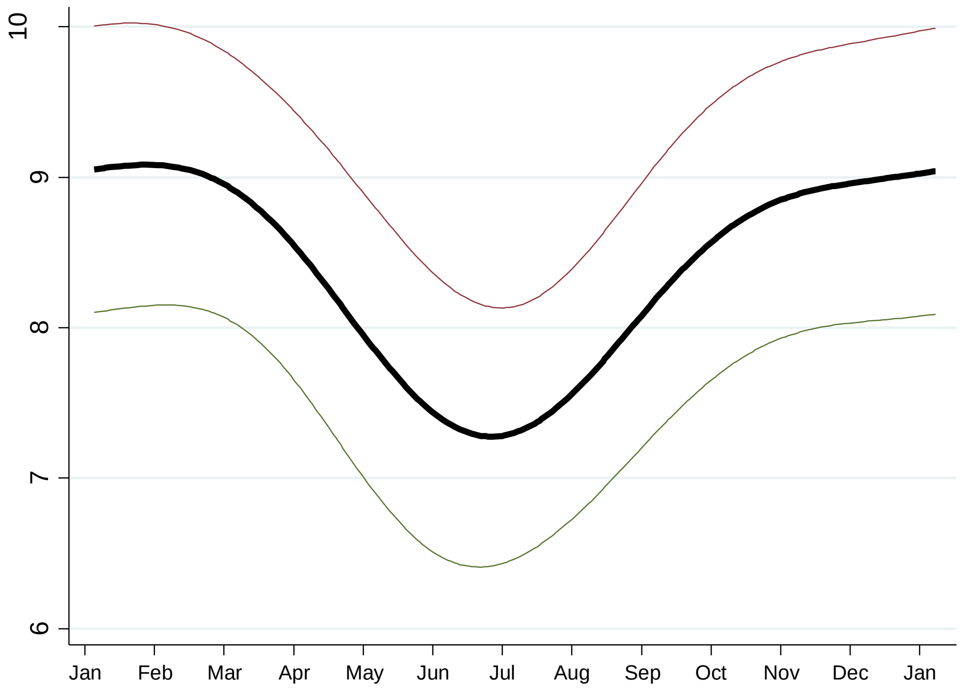

Fig. 1: Seasonal trend of plasma Hcy (with two pairs of Fourier Terms and 95% CIs), N=350

To design the supplement we analysed three separate datasets containing plasma nutrient concentration data from women in West Kiang. We ran multivariable linear regression models to assess which nutrients predicted plasma homocysteine. We selected those which consistently demonstrated an inverse relationship with homocysteine as candidates for the supplement. An overview of the datasets, methods and modelling results are provided in Appendix 1. The chosen supplement ingredients were then carefully assessed against existing dosage safety information, doses given in previous trials and current plasma nutrient levels to establish final supplement doses well below upper safety limits (detailed in 11.1). We also decided to test this novel supplement against a commonly used multiple micronutrient supplement, UNIMMAP, in a second trial arm. This is so that we can quantify the difference in effectiveness of both interventions and determine whether the especially-designed supplement is worth considering for future trials, or whether the existing UNIMAPP supplement is sufficient.

1.3 Potential risks and benefits

The potential risks to human subjects and known benefits, if any, are summarised in Section "Human Subject Protection".

2 Study objectives

Primary objective: To compare the effect of a novel micronutrient supplement taken daily for 12 weeks in the dry season on lowering plasma homocysteine (Hcy) versus the control group.

Secondary objectives:

- To compare the effect of a novel micronutrient supplement taken daily for 12 weeks versus an existing micronutrient supplement (UNIMMAP) on lowering plasma Hcy.
- To compare the effect of a novel micronutrient supplement taken daily for 5 weeks (mid-line) versus control on lowering plasma Hcy.
- To compare the effect of a novel micronutrient supplement taken daily for 5 weeks (mid-line) versus UNIMMAP on lowering plasma Hcy.
- To compare the effect of a novel micronutrient supplement on lowering blood pressure of women at 12 weeks versus the control arm.

2.1 Study endpoints

Primary endpoints: Plasma concentrations of homocysteine after 12 weeks of daily supplementation.

Secondary endpoints: Plasma concentrations of homocysteine after 5 weeks of daily supplementation. Blood pressure after 12 weeks of supplementation.

3 Study design

3.1 Type of study and design

This will be an unblinded 3-arm randomized controlled trial with 125 women per arm. Non-pregnant, non-lactating, healthy women of reproductive age in West Kiang will be randomized to 12 weeks of daily supplementation of either a) a novel micronutrient supplement, b) an existing available micronutrient supplement (UNIMMAP[2]) or c) no intervention (control) (Fig. 2).

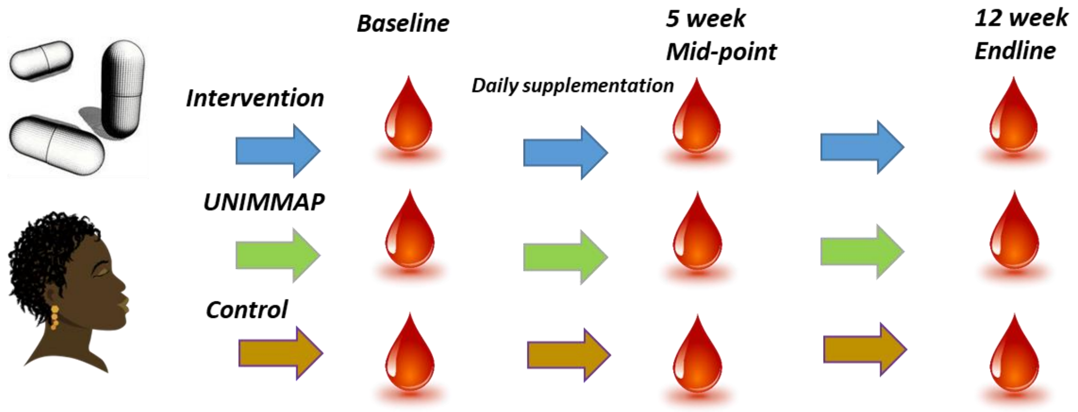

Fig 2. Trial overview

The supplements will be supplied to participants on a daily basis by Village Assistants (VAs). The VAs will observe consumption of the supplement. The novel micronutrient supplement will be provided in powder form with instructions to dissolve one sachet in 200ml of clean water. UNIMMAP will be provided in tablet form.

Women will provide one 10mL fasted venous blood sample at baseline and another after 5 and 12 weeks of supplementation. At each time point they will also have their blood pressure and anthropometry (weight and height) assessed and provide a urine pregnancy test. At baseline a questionnaire will capture socio-demographic information. Women will be brought to MRCG

[2] UNIMMAP is a widely used daily multiple micronutrient supplement given to pregnant women. It includes 15 micronutrients (vitamins A, D, E, B1, B2, B6, Bi 2, C, Niacin, Folic Acid, Fe, Zn, Cu, I, Se) at the Recommended Daily Allowance level.

Keneba for the baseline, 5week and 12 week data collection visits. Participants will be followed-up after the intervention period for a further 3 weeks to monitor any adverse effects.

Blood samples will be analysed for plasma homocysteine as per the primary and secondary objectives (figures 3 & 4, and section 2 above).

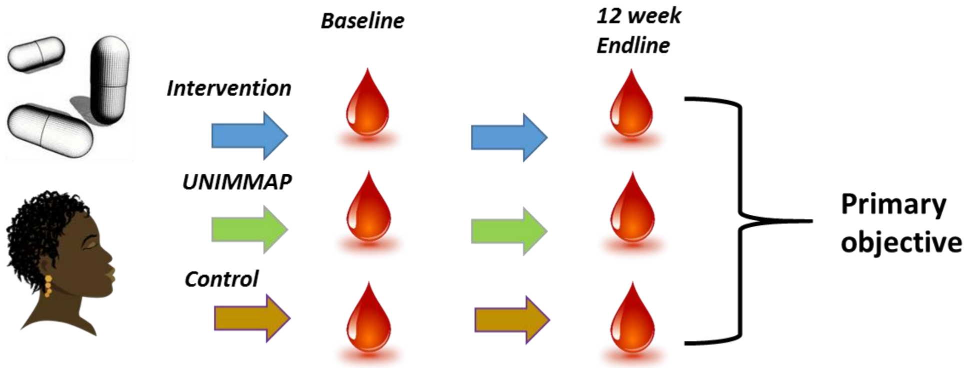

Fig. 3 Measurements performed to determine the primary objective

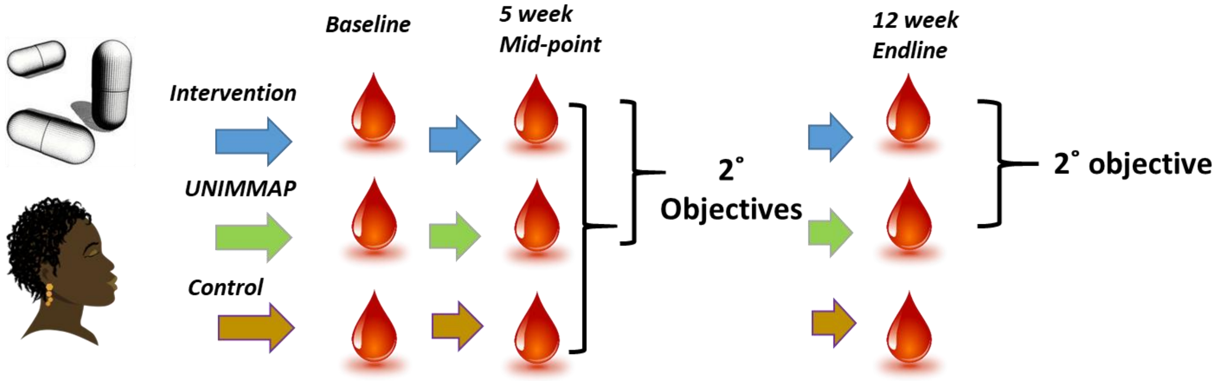

Fig. 4 Measurements performed to determine the secondary objectives

.

Please see Appendix 2 for the anticipated study flow diagram.

3.2 Randomisation and blinding procedures

3.2.1 Randomisation

Study participants will be randomized to the novel micronutrient supplement, UNIMMAP or control arm in a 1:1:1 ratio according to a computer generated randomization scheme. A list of IDs will be generated in advance by the Keneba database team with the oversight of the study statistician. This will comprise the 'study ID' and will include a two-letter study code, a 4-digit unique code and a check letter e.g. PT 1345 J. These IDs will be pre-assigned to one of three trial arms using block randomisation. After screening participants will be invited to MRC Keneba for their baseline visit. At

this point the study nurse will confirm eligibility and the participants will be enrolled onto the study and will be assigned their study ID. This will be done sequentially using the ID list in the order of enrolment. The field coordinator will be responsible to organise the field assistant team to ensure the implementation of the trial follows the treatment allocation assigned by the study IDs.

3.2.2 Blinding

Blinding will not be possible since the novel micronutrient supplement will be given as a drink powder, whereas UNIMMAP is supplied as a tablet. There will be no attempt to design a placebo drink or tablet, and the control arm will be used to identify the seasonal trend without any intervention. We expect plasma levels of homocysteine to fall by the end of the dry season and the control arm will capture the extent of this.

3.3 Sub-studies

N/A

3.4 Investigational products

3.4.1 Description of products

One study arm will consume the daily novel micronutrient supplement, to be taken dissolved in 200ml of water. Sugar will be provided to add to taste if participants find the taste too bitter. A second study arm will take the standard UNIMMAP formulation as a daily tablet.

3.4.2 Formulation, packaging and labelling

a) Novel micronutrient supplement formulation

Ingredient	Amount in supplement
Folate given as folic acid	800 µg per daily dose
Vitamin B12 given as cyanocobalamin	5.2 µg per daily dose
Riboflavin (vitamin B2) given as Riboflavin-5'-phosphate	2.8 mg per daily dose
Betaine given as anhydrous betaine	4g per daily dose

Packing: Individual pre-mixed sachets to be mixed with 200mL clean water

Labelling: TBC prior to Medical Control Agency registration.

b) **Daily United Nations Multiple Micronutrient Preparation (UNIMMAP) supplement:** This includes 15 micronutrients (vitamins A, D, E, B1, B2, B6, B12, C, Niacin, Folic Acid, Fe, Zn, Cu, I, Se) at the Recommended Daily Allowance level, except for folic acid, which is included at a level of 400 µg[(19)]. This comes in an existing commonly available tablet form.

Ingredient	Amount in supplement
Vitamin A	800 µg RE
Vitamin D	5 µg
Vitamin E	10 mg
Thiamine	1.4 mg
Riboflavin	1.4 mg
Niacin	18 mg
Folic Acid	400 µg
Vitamin B6	1.9 mg
Vitamin B12	2.6 µg
Vitamin C	70 mg
Zinc	15 mg
Iron	30 mg
Iodine	150 µg
Selenium	65 µg
Copper	2 mg

Packing: Jars of 60 tablets each.

Labelling:

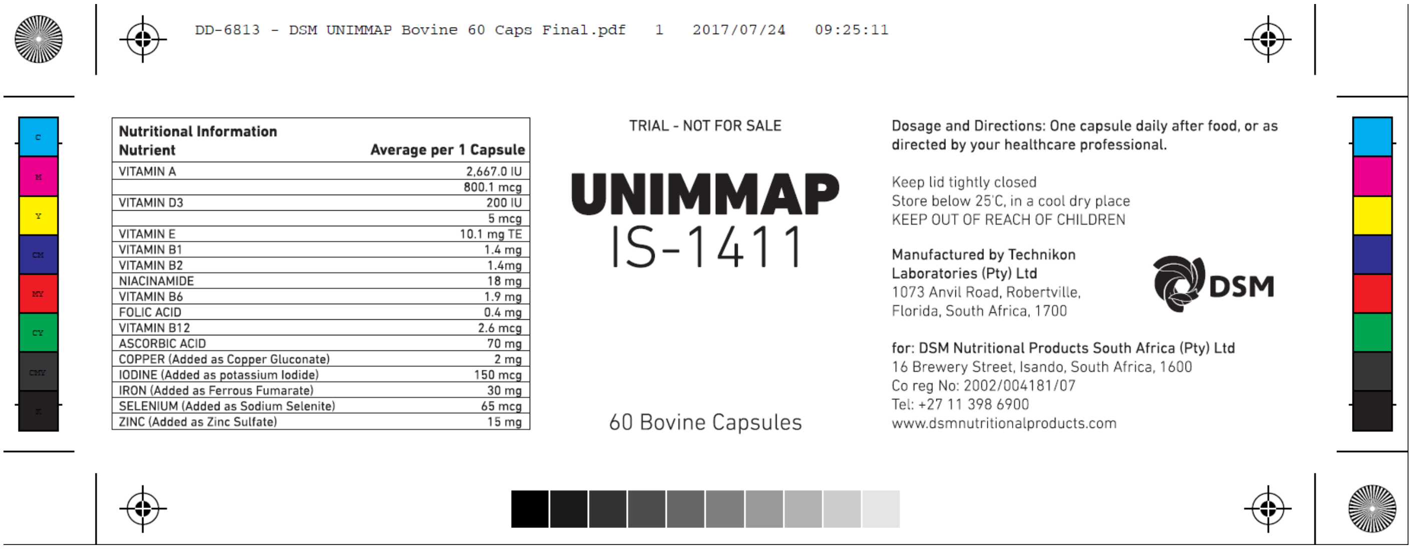

3.4.3 Product storage and stability

Storage of products will be at room temperature (20-25˚C), away from direct sunlight, at MRCG Keneba Field Station main store. The investigational products are stable when stored at this temperature, hence prior to study commencement the field assistants will ensure each VA has an adequate cool, dry place to store the products throughout the trial duration. The procured products have a two-year shelf-life after manufacture, and expiry dates will be carefully checked by the field supervisor and coordinator prior to study implementation.

3.4.4 Dosage, preparation and administration of investigational products

The products are designed to be given as a single daily dose, and composition details are given in section 3.4.2. The supplements will be supplied to the participants by the VAs for observed consumption. The VAs will be provided with cups marked to a 200mL line and spoons to prepare the micronutrient powder. Participants will be given 7 coloured cards at the start of each week by the VA. Every day they will give the VA one card and receive their supplement. Each week an MRC-employed field assistant will collect data on compliance by recording card collection in liaison with the VA.

3.4.5 Concomitant medications/treatments

None

4 Selection and withdrawal of participants

4.1 Selection of participants

Study participants will come from the 35 villages of West Kiang region. A list of eligible women within West Kiang will be generated using the MRCG Keneba Health and Demographic Surveillance System (HDSS) database. Women will be visited at their homes by field assistants to have the study and eligibility criteria explained to them and invited to join the study by providing consent.

4.2 Eligibility of participants

Participants must meet all of the inclusion criteria and none of the exclusion criteria to be eligible to participate in the trial. Inclusion and exclusion criteria will be assessed during recruitment by the field assistants. All potentially eligible women who provide consent will be invited to the baseline visit. At this visit a study nurse will assess pregnancy status using a rapid test kit. If the participant is confirmed as non-pregnant she will be formally enrolled onto the study, assigned a study ID and will continue will baseline measurements. A Study Specific Procedure will be developed to give more details on this process.

4.2.1 Inclusion criteria

- Pre-menopausal women aged 18-45 years
- Non-pregnant, confirmed by pregnancy urine test at the baseline visit
- Non-lactating (at least 9 months post-partum)
- No plan to conceive in the ensuing 3 months, asked verbally by field worker
- No plans to travel
- Healthy with no current illness and no chronic health problems, asked verbally by field worker

Note that although the supplements are considered safe for pregnant women (and plan to be trialled in pregnant women in the next phase), non-pregnant and non-lactating women are selected in an attempt to avoid difficulties in interpreting plasma biomarker concentrations, which can be altered during pregnancy and lactation.

4.2.2 Exclusion criteria

- Known history of chronic illness (particularly cardiovascular disease, renal disease, thyroid disease, cancer)
- Taking B vitamin or multivitamin supplements. Women who are taking B vitamins / multivitamins as part of a short course with other prescriptions can be assessed for eligibility again two weeks after completing their treatment.
- Taking medication for prevention of seizures (e.g. Carbamazepine).

A participant can still remain part of the study if she falls pregnant after the study has started (if she chooses to). The supplements pose no risk to her or her baby. However, to make the analysis more appropriate that subject will be excluded at the final analysis stage. Biomarker concentrations can change during pregnancy therefore we need to ensure the final analysis is restricted to those who are non-pregnant.

4.3 Withdrawal of participants

A study participant will be discontinued from participation in the study if:

- Any clinical significant adverse event (AE), laboratory abnormality, intercurrent illness, or other medical condition or situation occurs such that continued participation in the study would not be in the best interest of the participant as judged by the study clinician.
- Development of any exclusion criteria.

For further details on participant’s premature termination see corresponding section below.

Participants are free to withdraw from the study at any time without giving a reason.

5 Study procedures and evaluations

For an overview see “Schedule of Events”, Appendix 4.

5.1 Study schedule

The involvement of study participants will be restricted to 12 weeks and the novel micronutrient supplement or UNIMMAP tablet will be consumed daily with water for the entire duration. Participants will also be followed-up after the intervention period for a further 3 weeks to monitor any adverse events. At baseline, a pregnancy urine test will be used to confirm eligibility. If confirmed non-pregnant a questionnaire will be administered to collect basic information on socio-demographic information. Weight, height and blood pressure will be taken, followed by a fasted 10ml venepuncture. At 5 weeks and 12 weeks a further fasted 10mL venepuncture will be taken, as well as anthropometric and blood pressure readings and a pregnancy urine test. Baseline, 5 and 12 week data collection will take place at MRC Keneba, with transportation provided. A participant with abnormal blood pressure at any time-point will be referred to the clinic staff who will follow up the issue according to the usual standard of care. Spot checks will be carried out at the village level by field assistants and project management staff to ensure implementation is being carried out as per study protocol.

5.1.1 Screening

Potentially eligible participants (women age 18-45 years, non-pregnant, non-lactating) will be identified through the Keneba Demographic Surveillance System (DSS). Field assistants and a study nurse will visit the homes of potentially eligible participants, to provide full information about the purpose and methods of the study, potential risks and benefits, and participants' rights. Full inclusion and exclusion criteria will be assessed. This will take place in a private area or room to maintain privacy and confidentiality in the presence of an impartial witness. Participants will then be asked to provide a signed or thumb-printed informed consent and a copy of the signed consent document will be provided to her. All potentially eligible women providing consent will be invited to MRCG Keneba for a baseline assessment on a specific date.

5.1.2 Enrolment (Baseline)

Participants will be transported to MRCG Keneba for the baseline data collection. Pregnancy status will be confirmed from a urine sample by a study nurse using a rapid test kit. Women who are pregnant will be offered standard antenatal care. Those who are not pregnant can be formally enrolled onto the study. The baseline questionnaire asking demographic questions, blood pressure readings, anthropometric measurements and 10 mL venepuncture will be taken by trained data collectors and study nurses onsite. The participants will be assigned their study ID at this stage.

5.1.3 Follow-up

Novel micronutrient and UNIMMAP arms: participants will meet the VAs on a daily basis to take their supplement under observation. The participants will also have a fasted 10mL venepuncture, blood pressure measurements, anthropometric measurements and urine pregnancy test taken at week 5 at MRC Keneba.

Control arm: participants will visit MRC Keneba at week 5 for the 10mL venepuncture, blood pressure and anthropometric measurements.

5.1.4 Final study visit

The final study visit will be at week 12 and will be the same as the week 5 protocol, whereby a fasted 10mL venepuncture, pregnancy urine test, anthropometric measurements and blood pressure will be taken at MRC Keneba. Participants in the novel micronutrient and UNIMMAP arms will then be monitored by the VAs each week at their homes for three more weeks to check for any adverse events, detailed in section 6.1.1.

5.1.5 Early termination visit

An early termination visit may occur in this study because of a participant's voluntary withdrawal or at the discretion of the Safety Monitor according to the Study Specific Protocol, which will be developed separately. Apart from the safety evaluations, no other evaluations required for the final study visit will be done.

5.2 Study evaluations

5.2.1 Clinical evaluations

Clinical observations will include weight, height and blood pressure.

5.2.2 Laboratory evaluations

The fasted 10mL baseline, 5 week and 12 week blood samples will be taken by venepuncture into EDTA monovettes and kept on ice. Within one hour of collection the samples will be processed by the laboratory in Keneba to centrifuge the samples, separate the plasma and store plasma and red blood cell aliquots at -70°C. One plasma aliquot will be analysed in Keneba to measure homocysteine using the Cobas Integra 400 Plus analyser. One plasma aliquot and one red blood cell aliquot will be stored for future analyses on one-carbon biomarkers should additional funding for this be gained.

6 Safety considerations

6.1 Methods and timing for assessing, recording, and analysing safety parameters

This study will be conducted according to Good Clinical Practice (GCP) principles. The medical expert will be Dr Fatai Akemokwe, Head of Clinical Services at the MRCG field station in Keneba. The Local Safety Monitor will be Dr Karen Forrest. The trial will be reviewed by the Gambia Government/MRC Joint Ethics Committee.

6.1.1 Adverse events

An AE is defined as any untoward or unfavourable medical occurrence in a human subject, including signs and symptoms which are temporarily associated with the individual's participation in the

research, whether or not considered related to the individual's participation in the research. Participants will be asked verbally about AEs daily by the VAs during the study. Each week a field assistant will meet the VAs and summarise any AEs in a written report. There are no expected serious or moderate AEs associated with this study. However, a Study Specific Procedure will be drawn up to detail those symptoms that are more likely to be due to the intervention (e.g. urine more yellow in colour, unusual taste in mouth, nausea), and those that are likely unrelated (e.g. infection-related symptoms). This process will continue after the end of supplementation for a further 3 weeks. In the unlikely case of an AE the VAs will immediately call the field supervisor to liaise with the study nurse as to whether a clinic referral is required. VAs will be provided mobile phones for this purpose. All symptoms or signs reported or observed will be documented as an AE after evaluation by the study nurse or clinician. Any serious AE will be reported according to SOP-CTS-009 and followed up by study personnel until resolved or considered stable.

6.1.2 Reactogenicity

None

6.1.3 Serious adverse events (SAEs)

A SAE is any AE that is life-threatening or results in death or requires hospitalisation or prolongation of hospitalisation or is a persistent or significant disability/incapacity. Though none are expected in this study, all SAEs will be investigated by a clinician.

6.2 Reporting procedures

Dr Fatai Akemokwe will assess and document the severity or intensity of the AEs and laboratory changes as follows:

Grade	Description
Mild	Awareness of sign or symptom, but easily tolerated
Moderate	Enough discomfort to cause interference with usual activity
Severe	Incapacitating with inability to work or do usual activity
Life-threatening	This grade will be considered as SAE

The term "severe" is often used to describe the intensity (severity) of a specific event (as in mild, moderate, or severe myocardial infarction); the event itself, however, may be of relatively minor medical significance (such as severe headache). This is not the same as "serious", which is based on the outcome or criteria defined under the SAE definition. An event can be considered serious without being severe if it conforms to the seriousness criteria; similarly severe events that do not conform to the criteria are not necessarily serious. Seriousness (not severity) serves as a guide for defining regulatory reporting obligations. The PI/study coordinator shall report all SAEs without

filtration, whether or not related to the intervention, within 24 hours of becoming aware of the event to the Sponsor. If the SAE is related to the intervention, The Gambia/MRC Joint Ethics Committee will be notified within seven calendar days if fatal or life-threatening, and all others within 15 calendar days.

The minimum information required for this initial SAE report is:

- Trial number and (short) title
- Participant's ID
- Nature of the event
- Reporter's name

The PI/study coordinator will not wait for additional information to fully document the event before notifying. This initial report must be followed by a completed SAE Report within 2 working days, detailing relevant aspects of the SAE in question. All actions taken by the PI/study coordinator and the outcome of the event must also be reported immediately. For documentation of the SAE, any actions taken, outcome and follow-up, a SAE Report Forms will be used. All follow-up activities have to be reported, if necessary on one or more consecutive SAE report forms in a timely manner. All fields with additional or changed information must be completed and the report form should be forwarded to the Gambia/MRC Joint Ethics Committee within seven calendar days after receipt of the new information. Hospital case records and autopsy reports, including verbal autopsy, will be obtained where applicable.

6.3 Safety oversight

Safety oversight shall be provided by the Local Safety Monitor who will provide independent advice. There will be no Data Safety Monitoring Board (DSMB) for this low risk trial. The Safety Monitor will regularly review all AEs and SAEs. This review will focus particularly on AE's causality and reasons for losses to follow up, raising any concerns or issues that present immediate safety concern with the named investigators for reporting to the medical expert, while protecting the confidentiality of the trial data and the results of monitoring.

7 Discontinuation criteria

7.1 Participant's premature termination

Participants have the right to withdraw from the study at any time without giving a reason and this will not affect the medical care that would normally be received. The study team may also withdraw a participant from the study if deemed necessary at any time documenting the reason as one of the following:

- Serious Adverse Event

- Adverse Event
- Participant’s consent withdrawal
- Migrated/moved from the study area
- Lost to follow-up

A 'lost to follow-up' is any participant who completed all protocol specific procedures up to the administration of the investigational product or intervention, but was then lost during the follow-up period, with no safety information and no efficacy endpoint data ever became available.

In case the participant decides to withdraw participation or consent during the study, we will not work on participant’s samples without permission, but any information already generated from the samples until the time of withdrawal will be used and samples already collected, for which they have given consent, will also be analysed and data used. The study clinician may also ask for tests for the participant’s safety. The PI/trial coordinator will ask about the reason for any withdrawal and follow-up with the participant regarding any unresolved AEs. For withdrawn participants no specific data will be collected.

7.2 Study discontinuation

A Data Safety Monitoring Board is not required for this low risk trial. A Study Specific Procedure will be written to document how the Local Safety Monitor will oversee safety and outline possible reasons for termination. It will ultimately be the Sponsor’s decision to discontinue the study.

8 Statistical considerations

Using data from the ENID Trial booking samples[20] the dry season (February – April) mean Hcy is 8.31 µmol/L with a standard deviation (SD) of 2.1. This represents what we would expect for the control group. The rainy season (July-October) Hcy conc is 7.8 µmol/L with an SD of 3.5.

We have powered this study to detect a decrease of 1 µmol/L Hcy to bring the dry season concentration of Hcy to below the rainy season one. This is the minimum reduction we would envisage the supplement having, and is a realistic target based on available literature of the effects of betaine and folate supplementation on Hcy[15,17].

The sample size calculation for the primary objective (comparison of endline plasma Hcy novel micronutrient supplement vs. control) has the following parameters:

Control arm:	Mean Hcy concentration 8.31 µmol/L
Intervention arm:	Mean Hcy concentration 7.31 µmol/L
Standard deviation:	2.5 µmol/L*

Power: 80%
Confidence level: 5%
Test: Two-sample t-test

*Given the standard deviation of Hcy in the dry season can vary from 2.0 to 3.0 µmol/L according to the definition of the peak dry season months (see Appendix 3) we performed calculations for a range of SDs, shown in figure 4.

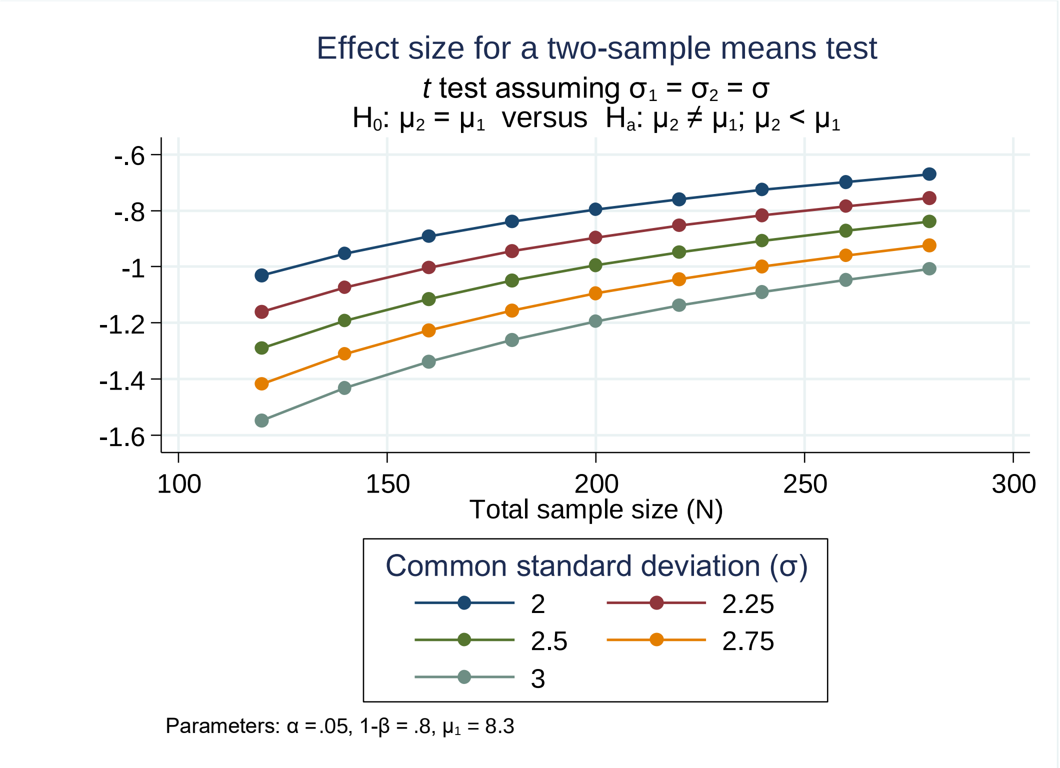

Fig 4: Sample size calculation using a range of SDs from 2.0 – 3.0

Using a SD of 2.5 with the above parameters the sample size is 100 women per arm. Estimating a 20% loss to follow up the final sample size is 125 women per arm. Additional sample size justifications are provided in Appendix 3.

Analysis of primary outcome:

We will use a linear regression model to determine the mean difference between intervention and control Hcy at 12 weeks adjusted for baseline Hcy, week 12 age and week 12 BMI. The general model will be:

Homocysteine concentration at 12 weeks = α + (β_1 x intervention group) + (β_2 x baseline Hcy) + (β_3 x week 12 BMI) + (β_4 x week 12 age)

where α is the mean Hcy in the control group when baseline Hcy, BMI and age take their respective mean values (all the three variables being centred at the respective mean), β_1 is the mean difference between the treatment and control groups, β_2, β_3 and β_4 represent the mean change in Hcy for a unit increase in baseline Hcy, BMI and age respectively.

Analysis plans for secondary outcomes will be developed separately. Intention-to-treat will be considered for primary reporting, although a sensitivity analysis will be performed to assess per-protocol results restricted to >80% compliance.

9 Data handling and record keeping

9.1 Data management and processing

All protocol required data will be captured in Case Report forms (CRF) that will be completed for each included participant. Data will be entered/checked by Data Entry Clerks with the supervision of the Data Manager.

On the CRF, a reference to the source document will be provided. Instructions for completing all forms, including the CRF, used in the study will be developed.

All data will be entered onto a secured, password-protected database that will only be accessed by the data manager, designated members of the trial team, Clinical Trial Monitor and the Sponsor. Paper CRFs will be locked in secure cabinets at the MRCG facilities. All data will be managed in accordance with the MRCG data management SOPs to ensure that the study is GCP compliant. These SOPs cover form submission, entry, verification, short-term and long term storage, retrieval and disposal.

9.2 Source documents and access to source data

The Principal Investigators will maintain appropriate medical and research records for this study in compliance with the principles of good clinical practice and regulatory and institutional requirements for the protection of confidentiality of participants. The study team members will have access to records.

The source documents will be defined for all data to be collected during the trial in a source data designation log. ID cards will be used to determine the age of the participant.

The authorised representatives of the sponsor, the ethics committee(s) or regulatory bodies may inspect all documents and records required to be maintained by the investigator, including but not limited to, medical records (office, clinic, or hospital) for the participants in this study. The clinical study site will permit access to such records.

9.3 Protocol deviations

A protocol deviation (PD) is any noncompliance with the clinical trial protocol, good clinical practice (GCP), or other applicable regulatory requirements. The noncompliance may be either on the part of the participant or the investigator including the study team members, and may result in significant added risk to the study participant. As a result of a deviation, corrective actions will be developed and implemented promptly in accordance with SOOP-CTS-010; Deviation Management in Clinical Trials.

If a deviation from, or a change of, the protocol is implemented to eliminate an immediate hazard(s) to trial participant without prior ethics approval, the PI or designee will submit the implemented deviation or change, the reasons for it, and, if appropriate, the proposed protocol amendment(s) as soon as possible to the sponsor for agreement and the relevant independent ethics committee (IEC) for review and approval.

The PI or designee will document and explain any deviation from the approved protocol on the CRF, where appropriate, and record and explain any deviation in a protocol deviation form that will be maintained as an essential document.

10 QUALITY CONTROL AND QUALITY ASSURANCE

Quality control will be applied to each stage of the study. It will be the responsibility of the PI/trial coordinator or designated trial team member to ensure that all source documents and CRFs are reviewed for accuracy and completeness. Any correction will be accurately accounted for.

Dispensing of the novel micronutrient supplement and UNIMMAP tablets to the village level, together with the anthropometric and blood pressure measurements will be done by the field worker or study nurse. Venous blood collection will be done by the study nurse or a clinician working with the trial team.

All procedures will be recorded on the CRF. All sample tubes will be labelled with ID barcodes. Quality control material will be run on the Cobas Integra equipment before analysing the samples in order to ensure precision and accuracy.

All Field assistants and their supervisors, study nurses and VAs will be trained. Weekly meetings of the entire trial field team will be convened in order to discuss all problems and lessons from the study.

10.1 Study monitoring

Risk-based trial monitoring will be conducted by the designated monitor of the MRCG Clinical Trials Support Office in keeping with the approved monitoring plan. The monitoring of study will follow the Unit's SOP-CTS-005 on Monitoring. An initiation visit will occur and formal study start approval obtained from the Sponsor before the start of recruitment. Interim visits will be conducted during the conduct of the study in line with the monitoring plan. At the end of the study, a close out visit will be conducted after last participant last visit and database cleaning completed and ready for database lock.

11 Ethical considerations

This study is conducted in accordance with the principles set forth in the ICH Harmonised Tripartite Guideline for Good Clinical Practice and the Declaration of Helsinki in its current version (see appendix 5), whichever affords the greater protection to the participants.

11.1 General considerations on human subject protection

Supplement doses for folic acid, B2 and B12 are proposed at twice the Recommended Daily Allowance (RDA) set for the United States of America(21) in order to correct the micronutrient deficiencies in the dry season.

Folic acid (proposed supplement 800 µg/d, 2xRDA):
Our proposed supplement dose has been used before in several large trials(22–24), and is well below the amount expected to cause adverse effects. The Expert Group on Vitamins & Minerals (2003) states that "in the general population a supplemental dose of 1 mg/day (equivalent to 0.017 mg/kg bw/day in a 60 kg adult) would not be expected to cause adverse effects"(25). The European Agency for Food Safety (2006) states that the Lowest observed-adverse-effect level (LOAEL) is 5 mg per day(26), and current US government advice for women who have previously had a baby with a birth defect is to take up to 4000 µg/d(27). Side effects have only been documented with doses in excess of 1mg/day.

Folic acid can decrease the effectiveness of the drugs used to prevent seizures: Fosphenytoin (Cerebyx), Phenobarbital (Luminal), Phenytoin (Dilantin), Primidone (Mysoline). It can also decrease the effectiveness of certain drugs for cancer treatment: Adrucil (Fluorouracil), Xeloda

(capecitabine), Methotrexate (MTX, Rheumatrex). Patients at risk of seizure or undergoing cancer treatment will be excluded from this trial.

Riboflavin (B2) (proposed supplement 2.8 mg/d, 2xRDA):

Riboflavin is Generally Recognised As Safe (GRAS), even when given in doses up to 14 times what we plan to give[28]. GRAS Report 114 on Riboflavin states that "there is no evidence in the available information on riboflavin or riboflavin-5'-phosphate that demonstrates or suggest reasonable grounds to suspect, a hazard to the public when they are used at levels that are now current or that might reasonably be expected in the future"[28]. The Expert Group on Vitamins & Minerals (2003) also concur, stating that "supplemental intakes of 40 mg riboflavin/day (equivalent to 0.67 mg/kg bw for a 60 kg adult) would be unlikely to result in adverse effects. This is in addition to riboflavin provided by the diet"[25]. There are no reported significant adverse effects to taking riboflavin supplements[26], however, some individuals may find their urine is more yellow in colour than normal.

Cyanocobalamin (B12) (proposed supplement 5.2 μg/d, 2xRDA):

The GRAS Report 104 on Cyanocobalamin states that "there is no evidence in the available information on vitamin B12 that demonstrates, or suggests reasonable grounds to suspect, a hazard to the public when it is used at levels that are now current and in the manner now practiced, or that might reasonably be expected in the future"[28] The Expert Group on Vitamins & Minerals (2003) states that "it is generally accepted that ingested vitamin B12 (cobalamin) has a very low toxicity in humans…supplemental 2.0 mg cyanocobalamin/day should not produce any adverse effects and this intake can be used for guidance purposes"[25]. There are no documented significant adverse effects of taking B12 supplement, even at doses several hundred times what we are planning to give[26].

Trimethylglycine (betaine powder) (proposed supplement 4g/d):

Trimethylglycine / betaine anhydrous is Generally Recognised As Safe and does not have a RDA. It has no common side effects nor drug interactions documented. In larger doses of 6g/day three small studies have reported a small rise of 10mg/dL low density lipoprotein cholesterol, however, this effect not been consistently seen in trials and is not seen to be a clinically significant side effect[17,29]. We do not anticipate any side effects with the lower dose of 4g/day. Similar doses have been used in trials to lower homocysteine in healthy adults before[17].

UNIMMAP: This is a widely used daily multiple micronutrient supplement, commonly given to pregnant women. It includes 15 micronutrients (vitamins A, D, E, B1, B2, B6, Bi 2, C, Niacin, Folic Acid, Fe, Zn, Cu, I, Se) at the RDA level. Given that the dosage of each micronutrient does not exceed

the United States RDA there are no concerns of toxicity for any of the components. This supplement has previously been safely used in several large trials[19,30–33], including in The Gambia[34].

11.1.1 Rationale for participant selection

Before testing the effect of the novel micronutrient supplement on the maternal metabolome and infant epigenome it is important to run a proof-of-concept trial. The participants enrolled in this pilot trial will be non-pregnant and non-lactating, selected as healthy volunteers to help test the efficacy of the supplement on lowering homocysteine and on supplementation compliance. If the supplement works well in this group it will provide the justification to scale up to a larger trial run in pregnant women.

11.1.2 Evaluation of risks and benefits

As outlined in section 11.1 none of the micronutrients provided in the novel micronutrient supplement have been reported to be associated with toxicity even in high doses. The possible risk in this study relates to blood collection i.e. pain or discomfort, bruising and infection. The total amount of blood to be collected is 10ml at baseline, 5 weeks and 12 weeks, which does not pose any health risk. Our study nurses are sufficiently trained and standard protocols will be used to avoid infection.

Participants who will receive the novel micronutrient supplement will likely have their homocysteine levels reduced. Elevated homocysteine is a risk factor for cardiovascular disease and cognitive decline, therefore reducing homocysteine may have beneficial effects beyond intergenerational epigenetic considerations. However, it is important to note at this stage it is still not clear whether homocysteine is simply a marker of disease or whether it is indeed causally associated[35]. In the ENID trial upon enrolment 25.7% of women had a low plasma folate concentration (<10nmol/L), almost all had a low plasma riboflavin concentration (geometric mean 13.6 nmol/L), and 24.3% had a low betaine plasma concentration (<16 µmol/L). It is therefore expected that the novel supplement will improve stores of these micronutrients. Those taking UNIMMAP may also improve their status of the 15 micronutrients the supplement includes.

11.2 Informed consent

Individual consent for the study will be sought. Field workers will be trained to explain the project in full detail to the eligible participants, covering all aspects of such study as laid out in the enclosed ‘Information Sheet’. Literate participants will then be given the Information Sheet while illiterate participants will have the full Information Sheet read to them in the language they understand. Illiterate consenting subjects will require an independent literate witness. Any questions that arise will be answered by the fieldworkers, and participants will also be given the possibility to obtain further clarifications and explanations by speaking to one of the study investigators if they wish.

Participation is entirely voluntary and we do not intend to enrol individuals who are not able to give consent. If participants agree to participate, written Informed Consent will be obtained.

11.3 Participant confidentiality

Participant confidentiality, privacy and anonymity will be ensured at all times, and the standards set by the MRCG will be followed. All data will be anonymised and individuals will not be identifiable.

11.4 Future use of stored specimen

An aliquot will be stored for a further 5 years. If further funding is successful tertiary objectives could be looked into under a separate SCC application.

12 Financing and insurance

The research related costs of the proposed trial will be covered by the MRC grant MC_EX_MR/M01424X/1: 'Impact of maternal diet on the epigenome'. LSHTM will sponsor the study and such participants will be protected in accordance with MRCG provisions.

13 Publication policy

All key findings from this study will be submitted for publication in peer-reviewed journals.

14 References

1. Jaenisch R & Bird A (2003) Epigenetic regulation of gene expression: how the genome integrates intrinsic and environmental signals. *Nat. Genet.* **33 Suppl**, 245–254.

2. Desai M, Jellyman JK & Ross MG (2015) Epigenomics, gestational programming and risk of metabolic syndrome. *Int. J. Obes.*, 1–9.

3. Daxinger L & Whitelaw E (2012) Understanding transgenerational epigenetic inheritance via the gametes in mammals. *Nat. Rev. Genet.* **13**, 153–62.

4. Heijmans BT, Tobi EW, Stein AD, et al. (2008) Persistent epigenetic differences associated with prenatal exposure to famine in humans. *Proc. Natl. Acad. Sci. U. S. A.* **105**, 17046–9.

5. Tobi EW, Slieker RC, Stein AD, et al. (2015) Early gestation as the critical time-window for changes in the prenatal environment to affect the adult human blood methylome. *Int. J. Epidemiol.* **44**, 1211–1223.

6. Langley-Evans SC (2015) Nutrition in early life and the programming of adult disease: a review. *J. Hum. Nutr. Diet.* **28**, 1–14.

7. Seisenberger S, Peat JR, Hore TA, et al. (2013) Reprogramming DNA methylation in the mammalian life cycle: building and breaking epigenetic barriers. *Philos. Trans. R. Soc. Lond. B. Biol. Sci.* **368**, 20110330.

8. Steegers-Theunissen RPM, Twigt J, Pestinger V, et al. (2013) The periconceptional period, reproduction and long-term health of offspring: the importance of one-carbon metabolism. *Hum. Reprod. Update* **19**, 640–55.

9. Moore SE, Cole TJ, Poskitt EME, et al. (1997) Season of birth predicts mortality in rural Gambia. *Nature* **388**, 434.

10. Dominguez-Salas P, Moore SE, Baker MS, et al. (2014) Maternal nutrition at conception modulates DNA methylation of human metastable epialleles. *Nat. Commun.* **5**, 3746.

11. Silver MJ, Kessler NJ, Hennig BJ, et al. (2015) Independent genomewide screens identify the tumor suppressor VTRNA2-1 as a human epiallele responsive to periconceptional environment. *Genome Biol.* **16**, 118.

12. Kühnen P, Handke D, Waterland RA, et al. (2016) Interindividual Variation in DNA Methylation at a Putative POMC Metastable Epiallele Is Associated with Obesity. *Cell Metab.* **24**, 502–9.

13. Dominguez-Salas P, Moore SE, Cole D, et al. (2013) DNA methylation potential: dietary intake and blood concentrations of one-carbon metabolites and cofactors in rural African women. *Am. J. Clin. Nutr.* **97**, 1217–27.

14. Mason JB (2003) Biomarkers of Nutrient Exposure and Status in One-Carbon (Methyl) Metabolism. *J. Nutr.* **133**, 941S–947.

15. Homocysteine Lowering Trialists' Collaboration (1998) Lowering blood homocysteine with folic acid based supplements: meta-analysis of randomised trials. *BMJ* **316**, 894–8.

16. Huang T, Zheng J, Chen Y, et al. (2011) High consumption of Ω-3 polyunsaturated fatty acids decrease plasma homocysteine: a meta-analysis of randomized, placebo-controlled trials. *Nutrition* **27**, 863–7.

17. McRae MP (2013) Betaine supplementation decreases plasma homocysteine in healthy adult participants: a meta-analysis. *J. Chiropr. Med.* **12**, 20–25.

18. James P, Silver M & Prentice A (2017) Epigenetics, Nutrition and Infant Health. In *Biol. First 1000 Days* [Karakochuk C, Whitfield K, Green T, et al., editors]. Taylor & Francis Group.

19. Osrin D, Vaidya A, Shrestha Y, et al. (2005) Effects of antenatal multiple micronutrient supplementation on birthweight and gestational duration in Nepal: double-blind, randomised controlled trial. *Lancet* **365**, 955–62.

20. Moore SE, Fulford AJ, Darboe MK, et al. (2012) A randomized trial to investigate the effects of pre-natal and infant nutritional supplementation on infant immune development in rural Gambia: the

ENID trial: Early Nutrition and Immune Development. *BMC Pregnancy Childbirth* **12**, 107.

21. Institute of Medicine (US) Committee to Review Dietary Reference Intakes for Vitamin D and Calcium (2011) *Dietary Reference Intakes for Calcium and Vitamin D*. *Diet. Ref. Intakes Calcium Vitam. D* [Ross A, Taylor C, Yaktine A, editors]. Washington (DC): National Academies Press.

22. Ebbing M, Bønaa KH, Nygård O, et al. (2009) Cancer incidence and mortality after treatment with folic acid and vitamin B12. *JAMA* **302**, 2119–26.

23. Fawzi WW, Msamanga GI, Urassa W, et al. (2007) Vitamins and perinatal outcomes among HIV-negative women in Tanzania. *N. Engl. J. Med.* **356**, 1423–31.

24. Czeizel AE, Dudás I & Métneki J (1994) Pregnancy outcomes in a randomised controlled trial of periconceptional multivitamin supplementation. Final report. *Arch. Gynecol. Obstet.* **255**, 131–9.

25. Expert Group on Vitamins and Minerals (EVM) (2003) *Safe Upper Levels for Vitamins and Minerals*. UK: Food Standards Agency.

26. European Food Safety Authority (2006) *Tolerable upper intake levels for vitamins and minerals*. Scientific Committee on Food Scientific Panel on Dietetic Products, Nutrition and Allergies.

27. Office on Women's Health (2012) Folic Acid Fact Sheet. *womenshealth.gov*. https://www.womenshealth.gov/publications/our-publications/fact-sheet/folic-acid.html (accessed January 2017).

28. US Food and Drug Administration (1972) *GRAS Substances (SCOGS) Database.* Select Committee on GRAS Substances (SCOGS). Center for Food Safety and Applied Nutrition.

29. Zeisel SH (2006) Betaine Supplementation and Blood Lipids: Fact or Artifact? *Nutr. Rev.* **64**, 77–79.

30. Ramakrishnan U, González-Cossío T, Neufeld LM, et al. (2003) Multiple micronutrient supplementation during pregnancy does not lead to greater infant birth size than does iron-only supplementation: a randomized controlled trial in a semirural community in Mexico. *Am. J. Clin. Nutr.* **77**, 720–5.

31. Friis H, Gomo E, Nyazema N, et al. (2004) Effect of multimicronutrient supplementation on gestational length and birth size: a randomized, placebo-controlled, double-blind effectiveness trial in Zimbabwe. *Am. J. Clin. Nutr.* **80**, 178–84.

32. Kaestel P, Michaelsen KF, Aaby P, et al. (2005) Effects of prenatal multimicronutrient supplements on birth weight and perinatal mortality: a randomised, controlled trial in Guinea-Bissau. *Eur. J. Clin. Nutr.* **59**, 1081–9.

33. Christian P, West KP, Khatry SK, et al. (2003) Effects of maternal micronutrient supplementation on fetal loss and infant mortality: a cluster-randomized trial in Nepal. *Am. J. Clin. Nutr.* **78**, 1194–202.

34. Cooper WN, Khulan B, Owens S, et al. (2012) DNA methylation profiling at imprinted loci after periconceptional micronutrient supplementation in humans: results of a pilot randomized controlled trial. *FASEB J.* **26**, 1782–90.

35. Hannibal L & Blom HJ (2017) Homocysteine and disease: Causal associations or epiphenomenons? *Mol. Aspects Med.* **53**, 36–42.

36. Waterland RA, Kellermayer R, Laritsky E, et al. (2010) Season of conception in rural gambia affects DNA methylation at putative human metastable epialleles. *PLoS Genet.* **6**, e1001252.

Supplements, appendices and other documents

Appendix 1: Predictors of plasma homocysteine datasets, methods and results overview

Datasets Used

Dataset	Total N	Dry Season N	Rainy Season N	Biomarkers included in model	A priori confounders forced into all models	Models used	Notes
**Indicator group**[13]	48 women with 288 total observations	34 women with 79 observations	28 women with 63 observations	Hcy, B2, PLP (B6), B12, folate, methionine, choline, betaine, DMG, cysteine	Maternal age	Linear regression with random effects. 2-step approach only	Non-pregnant women, roughly 30 followed for one year, longitudinal study with monthly blood samples. Not able to perform stepwise and LASSO in STATA with panel data.
**MDEG1**[10]	167	83	84	Hcy, B2, PLP (B6), B12, folate, methionine, choline, betaine, DMG, cysteine	Maternal age, BMI, gestational age	Ordinary linear regression. 2-step, backwards stepwise and LASSO	Cross-sectional data, pregnant women selected from peak of rainy and peak of dry season
**MDEG2**<br><br>Using ENID data[20]	350	87	100	Hcy, B2, PLP (B6), B12, folate, methionine, choline, betaine, DMG, cysteine, Asp, Thr, Ser, Glu, Gly, Ala, Val, Ile, Leu, Tyr, Phe, Lys, His, Arg, Pro	Maternal age, BMI, gestational age, inflammation (AGP)	Ordinary linear regression. 2-step, backwards stepwise and LASSO	Cross sectional data, pregnant women selected from each month of year

Methods overview for deciding which micronutrients to include in the novel supplement

**Outcome**	All models use log Hcy as the dependent variable
**Independent variables**	All models use standardised log-transformed variables
**Effect sizes**	All coefficients represent change in log Hcy for a one SD increase in the independent variable
**2-step model**	In univariable linear regression regress log Hcy against an independent variable, adjusting for confounders<br>Any variables with p<0.1 are taken forwards to a multivariable linear regression model, adjusted for confounders<br>Final results reported are independent variables with p<0.05 in the Wald test.
**Stepwise**	Backwards step-wise approach.<br>Criteria for removal from model is p>0.2.<br>Final results reported are retained independent variables with p<0.05 in the Wald test.<br>A priori confounders are forced into final model
**LASSO**	STATA determines the number of variables to include depending on the lowest Cp statistic it finds<br>No individual p values are reported, output given contains the coefficients only<br>Given it's not possible to force confounders into final model, therefore I pre-adjusted log Hcy by regressing it against the confounders and used the residuals in the LASSO model

Results overview: predictors of homocysteine in three datasets

Coefficients represent change in log homocysteine (Hcy) for a one SD increase in the independent variable. Only results p<0.05 in mulitvariable analysis included below. Red boxes represents biomarkers positively associated with Hcy and green boxes for those negatively associated with homocysteine. Shading is proportional to the effect size. Those consistently negatively associated with Hcy were considered for the supplement.

	Combined seasons						
Biomarker	MDEG2 2-step	MDEG2 Stepwise	MDEG2 LASSO	MDEG1 2-step	MDEG1 Stepwise	MDEG1 LASSO	Indicator 2-step
Cysteine	0.2	0.2	0.18	0.14	0.16	0.13	0.14
DMG	0.03	0.04	0.03	0.05		0.03	0.08
Folate	-0.1	-0.1	-0.09	-0.12	-0.14	-0.09	-0.11
B2		-0.05	-0.04	-0.05	-0.04	-0.02	
Choline						0.04	
B12	-0.1	-0.1	-0.08		-0.05		
PLP		0.04	0.04			0.03	
Betaine	-0.07	-0.07	-0.06			-0.05	-0.05
Glycine	0.05	0.06	0.06				
Glutamate			0.02				
Proline		0.04	0.03				
Aspartate		0.02	0.02				
Tyrosine			-0.02				
Alanine							
Serine							
Threonine			-0.02				
Methionine							
Valine							
Leucine							

	Dry season						
Biomarker	MDEG2 2-step	MDEG2 Stepwise	MDEG2 LASSO	MDEG1 2-step	MDEG1 Stepwise	MDEG1 LASSO	Indicator 2-step
Cysteine	0.18	0.18	0.16	0.16	0.16	0.13	0.17
DMG				0.04	0.04	0.05	
Folate		-0.08	-0.06	-0.17	-0.18	-0.11	-0.15
B2		-0.05		-0.07	-0.06	-0.06	
Choline			-0.04			0.06	
B12	-0.08	-0.07	-0.06		-0.05	-0.02	
PLP		0.05	0.07			-0.02	
Betaine	-0.07	-0.08	-0.06			-0.11	
Glycine			0.02				
Glutamate							
Proline			0.04				
Aspartate		0.05	0.02				
Tyrosine							
Alanine							
Serine			0.03				
Threonine		-0.04	-0.03				
Methionine						0.03	
Valine							
Leucine							

Appendix 2: Schematic of Planned Study Flow

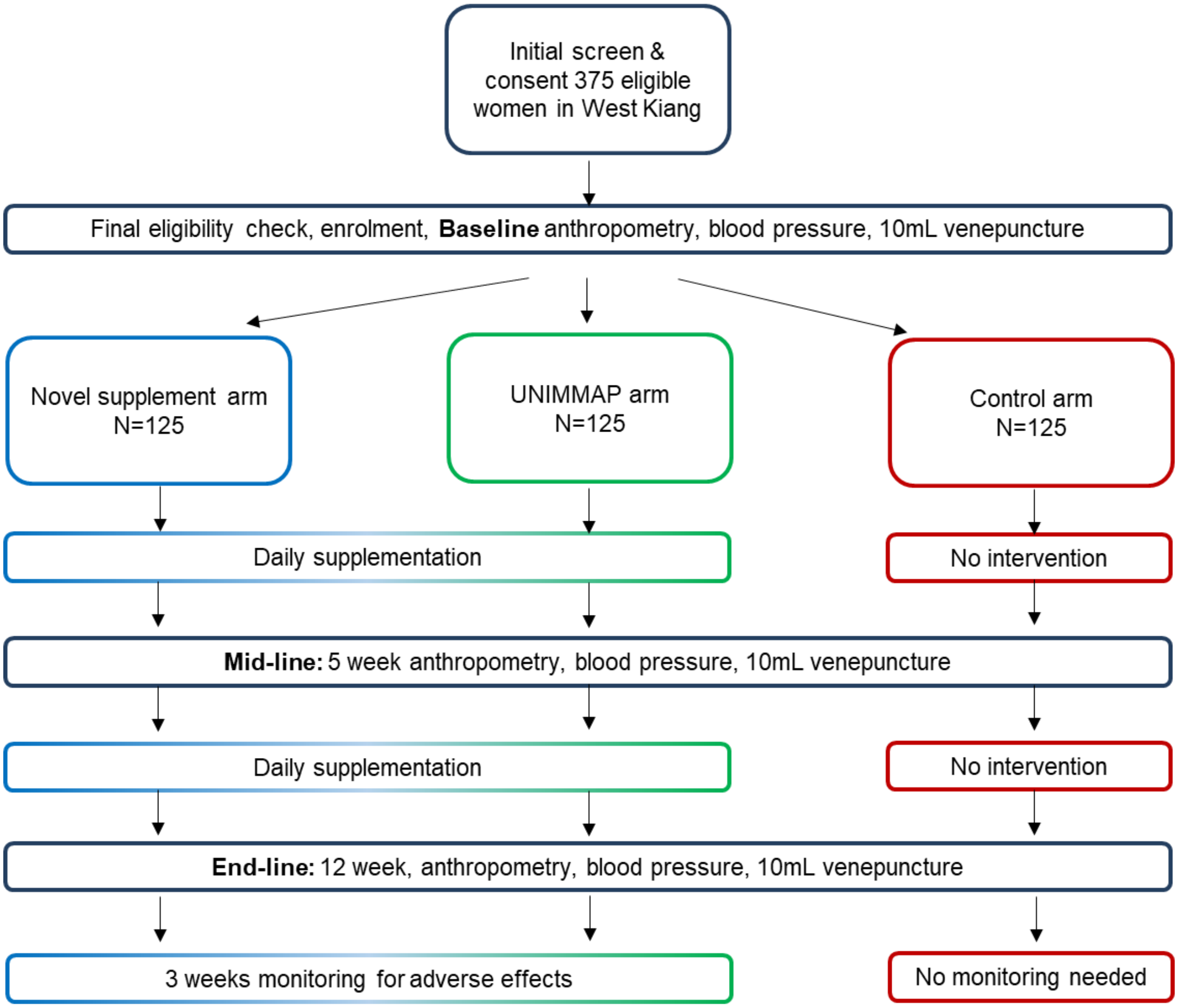

Appendix 3: Additional sample size justification

Raw data from MDEG2 dataset (ENID booking samples):

<table>
<tr><th></th><th></th><th></th><th></th><th colspan="3">Dry season bands (Mean, SD)</th><th colspan="3">Rainy season bands (Mean, SD)</th></tr>
<tr><th>Month of booking</th><th>N</th><th>Mean Hcy (μmol/L)</th><th>SD</th><th>Jan-Mar</th><th>Feb-Apr</th><th>Mar-May</th><th>June-Aug</th><th>July-Sept</th><th>Aug-Oct</th></tr>
<tr><td>Jan</td><td>25</td><td>9.47</td><td>4.22</td><td rowspan="3">8.51 (2.88)</td><td></td><td></td><td></td><td></td><td></td></tr>
<tr><td>Feb</td><td>25</td><td>8.31</td><td>2.07</td><td rowspan="3">8.31 (2.11)</td><td></td><td></td><td></td><td></td></tr>
<tr><td>Mar</td><td>35</td><td>7.99</td><td>1.98</td><td rowspan="3">8.42 (2.35)</td><td></td><td></td><td></td></tr>
<tr><td>Apr</td><td>27</td><td>8.73</td><td>2.30</td><td></td><td></td><td></td><td></td></tr>
<tr><td>May</td><td>21</td><td>8.75</td><td>2.93</td><td></td><td></td><td></td><td></td><td></td></tr>
<tr><td>Jun</td><td>32</td><td>7.20</td><td>3.83</td><td></td><td></td><td></td><td rowspan="3">7.30 (2.74)</td><td></td><td></td></tr>
<tr><td>Jul</td><td>38</td><td>7.24</td><td>2.19</td><td></td><td></td><td></td><td rowspan="3">7.82 (3.48)</td><td></td></tr>
<tr><td>Aug</td><td>36</td><td>7.44</td><td>2.10</td><td></td><td></td><td></td><td rowspan="3">8.31 (3.68)</td></tr>
<tr><td>Sep</td><td>26</td><td>9.20</td><td>5.65</td><td></td><td></td><td></td><td></td></tr>
<tr><td>Oct</td><td>29</td><td>8.58</td><td>2.77</td><td></td><td></td><td></td><td></td><td></td></tr>
<tr><td>Nov</td><td>27</td><td>8.77</td><td>3.27</td><td></td><td></td><td></td><td></td><td></td><td></td></tr>
<tr><td>Dec</td><td>28</td><td>8.00</td><td>2.38</td><td></td><td></td><td></td><td></td><td></td><td></td></tr>
</table>

Take the three different dry season bands to get an idea of some expected means and SDs of the controls. Then calculate the number required per arm to reduce Hcy by 1 μmol/L. This would bring the dry season means towards the rainy season means. We would expect the supplement to do that as a minimum effect size.

Control estimate	alpha	power	Mean Control	Mean Intervention	SD	Intervention N	Control N	Total N
Jan-Mar	.05	.8	8.51	7.51	2.88	132	132	264
Feb-Apr	.05	.8	8.31	7.31	2.11	71	71	142
Mar-May	.05	.8	8.42	7.42	2.35	88	88	176

- Use a control mean of 8.31 μmol/L going forwards. Seems a fair representation of what we could expect, and is a similar dry season mean to other datasets too.
- We don't know the exact SD so we can see the effect of a SD ranging from 2.0-3.0.
- The below graph shows how varying the SD and the sample sizes influences the difference we are able to detect at 80% power and 5% confidence.

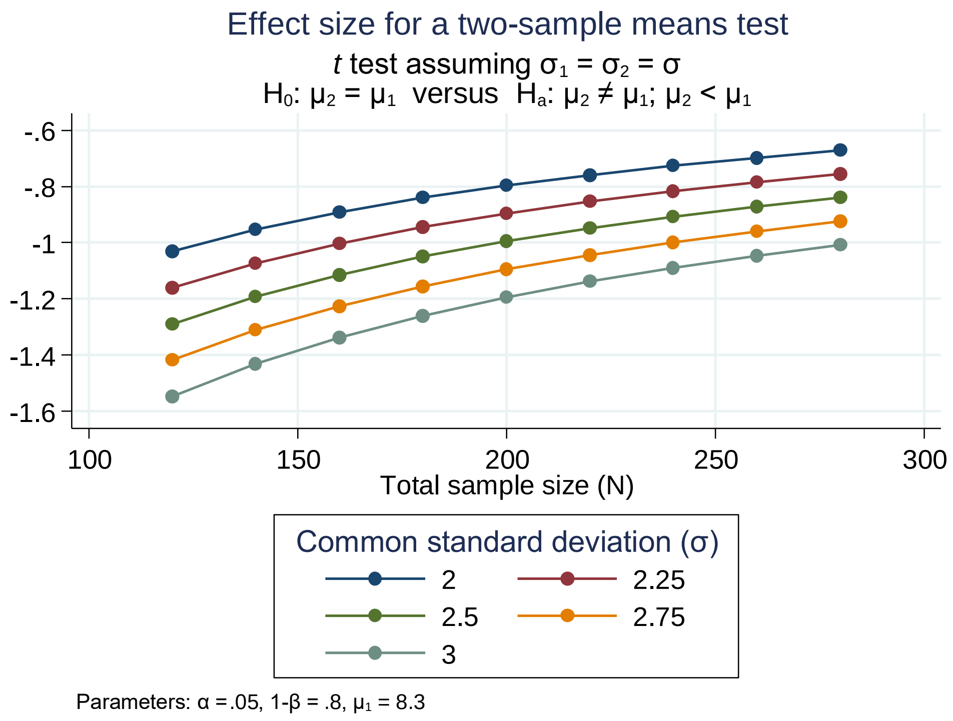

Decision: Happy with looking at a sample size of 100 in each arm (200 total) with an SD of 2.5 to be conservative.

- What power do we have to detect differences with this set-up?

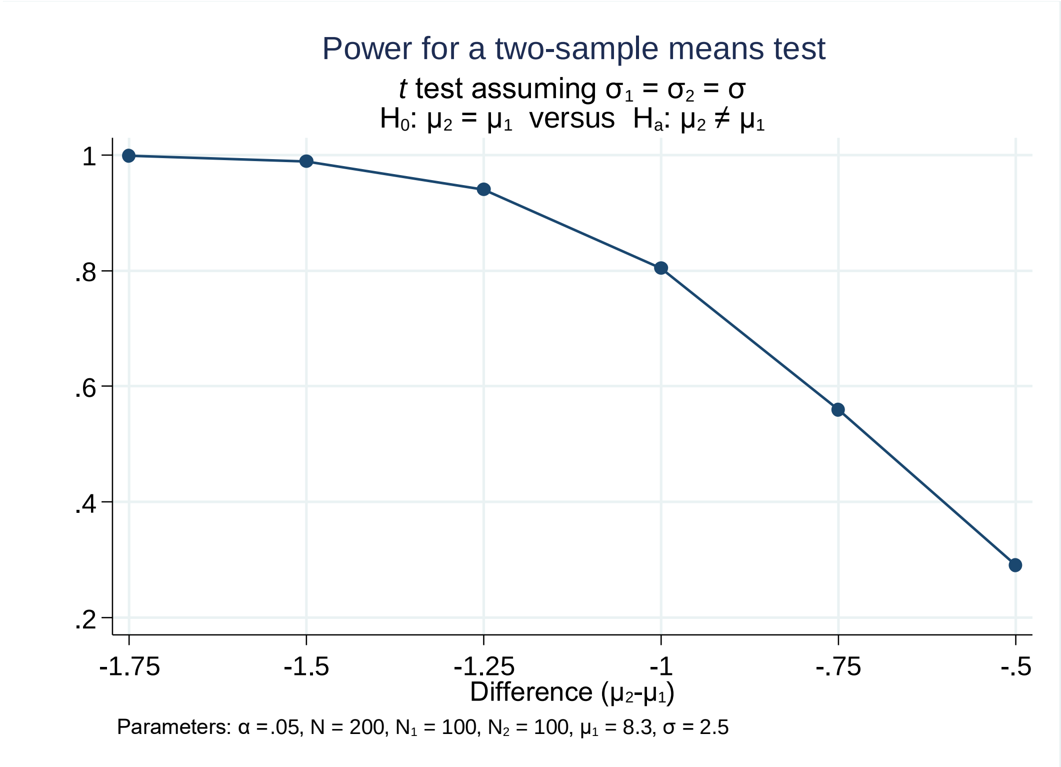

Summary: A sample size of 100 per arm should be able to detect 1µmol/L difference between intervention and control with SD of 2.5 with 80% power.

Account for loss to follow up of 20%: **N=125 per arm**

Secondary objective:

- With these parameters we can check our secondary objective of comparisons by time point (6 week and 12 week intervention vs. control) and also different arms (UNIMAPP vs intervention; intervention vs. control).
- Use Bonferroni correction for these tests *i.e.* alpha now = 0.025
- With alpha of 0.025, SD of 2.5, N of 200 and control mean of 8.31µmol/L what difference can we detect and at what power?

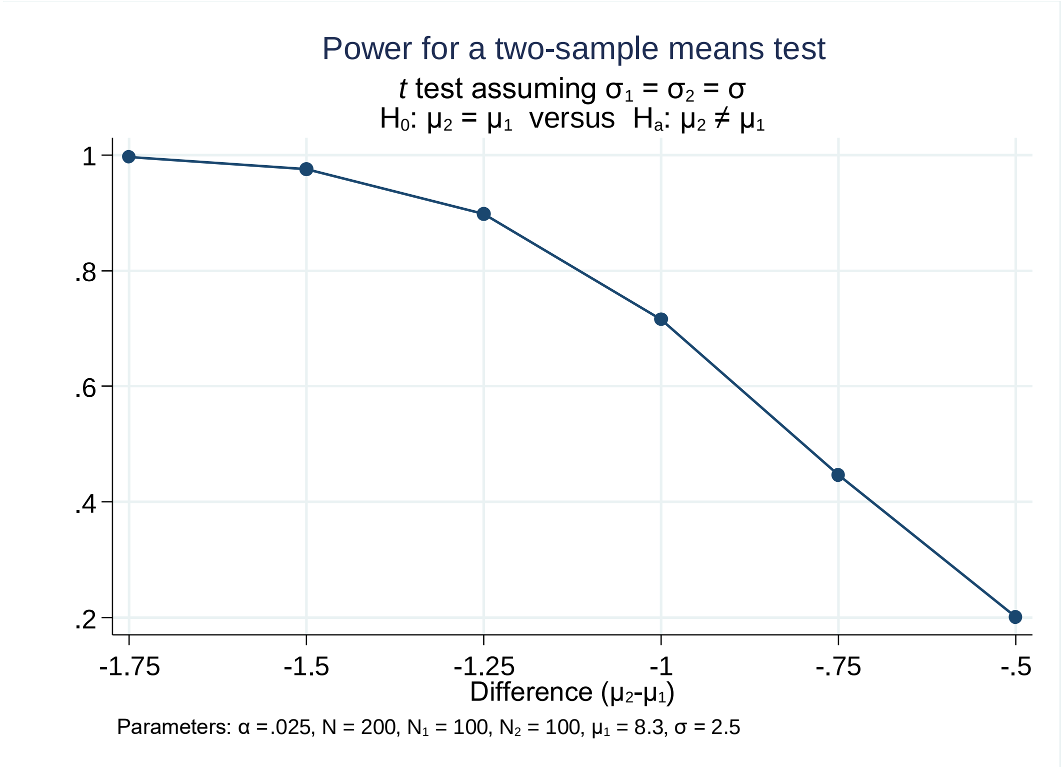

- We would have 80% power to detect a difference of 1.125 µmol/L, and 70% power to detect a 1µmol/L difference.

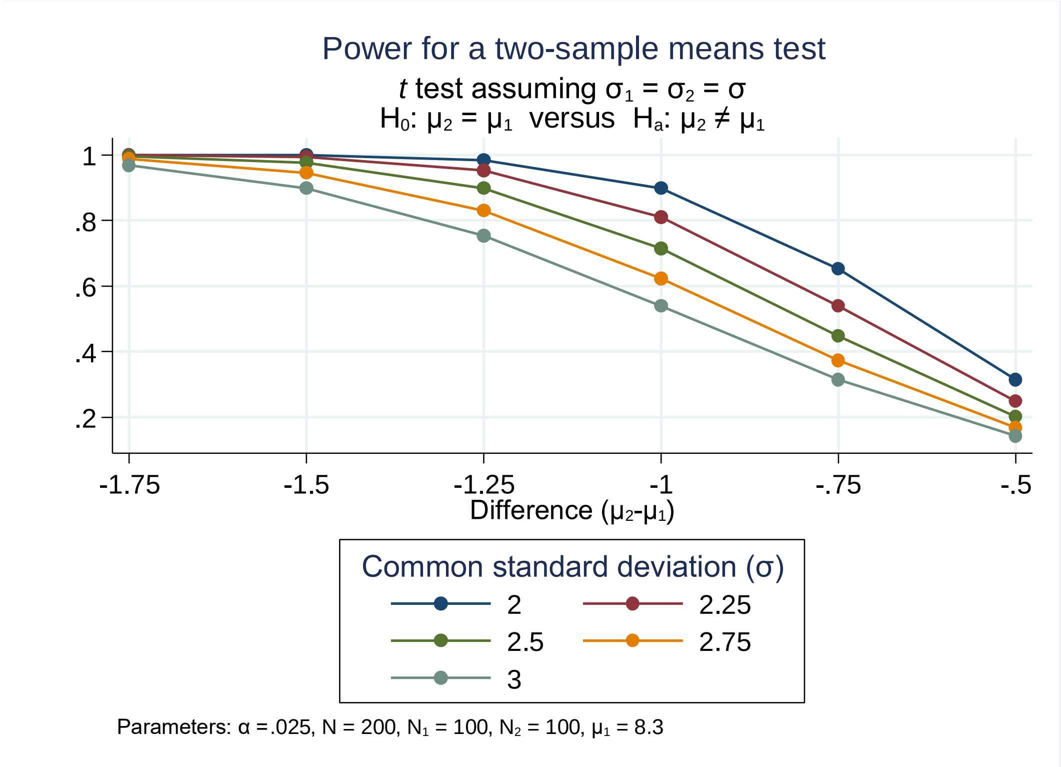

- The above graph shows we would still have 80% power to detect a difference of 1µmol/L if the SD is 2.25µmol/L. So this gives us increased confidence our sample size of 100 per arm will be good enough for our purposes.
- In an ideal scenario we would have alpha (0.025) power of 80% SD of 2.5 and difference to detect of 1µmol/L
- This would require 121 women per arm.
- Total needed with 20% loss to follow up would be: 152 women per arm. This is pushing the boundaries of what is possible to recruit.

Decision: recruit 125 women per arm.

Appendix 4: Schedule of events

**Procedure**	**Baseline**	**Weeks 1-4**	**Week 5**	**Weeks 6-11**	**Week 12**	**Weeks 13-15**
**Consenting**	X					
**Enrolment**	X					
**Randomisation**	X					
**Supply of intervention***	X	X	X	X	X	
**Weight**	X		X		X	
**Height**	X		X		X	
**Blood pressure**	X		X		X	
**Venepuncture**	X		X		X	
**Pregnancy test**	X		X		X	
**Socio-demographic questionniare**	X					
**AE monitoring**		X	X	X	X	X

*The supplements will be provided daily by Village Assistants

Appendix 5:

WORLD MEDICAL ASSOCIATION DECLARATION OF HELSINKI
Ethical Principles for Medical Research Involving Human Subjects

Adopted by the 18th WMA General Assembly, Helsinki, Finland, June 1964
and amended by the:
29th WMA General Assembly, Tokyo, Japan, October 1975
35th WMA General Assembly, Venice, Italy, October 1983
41st WMA General Assembly, Hong Kong, September 1989
48th WMA General Assembly, Somerset West, Republic of South Africa, October 1996
52nd WMA General Assembly, Edinburgh, Scotland, October 2000
53rd WMA General Assembly, Washington DC, USA, October 2002 (Note of Clarification added)
55th WMA General Assembly, Tokyo, Japan, October 2004 (Note of Clarification added)
59th WMA General Assembly, Seoul, Republic of Korea, October 2008
64th WMA General Assembly, Fortaleza, Brazil, October 2013

Preamble

1. The World Medical Association (WMA) has developed the Declaration of Helsinki as a statement of ethical principles for medical research involving human subjects, including research on identifiable human material and data.

The Declaration is intended to be read as a whole and each of its constituent paragraphs should be applied with consideration of all other relevant paragraphs.

2. Consistent with the mandate of the WMA, the Declaration is addressed primarily to physicians. The WMA encourages others who are involved in medical research involving human subjects to adopt these principles.

General Principles

3. The Declaration of Geneva of the WMA binds the physician with the words, “The health of my patient will be my first consideration,” and the International Code of Medical Ethics declares that, “A physician shall act in the patient's best interest when providing medical care.”

4. It is the duty of the physician to promote and safeguard the health, well-being and rights of patients, including those who are involved in medical research. The physician's knowledge and conscience are dedicated to the fulfilment of this duty.

5. Medical progress is based on research that ultimately must include studies involving human subjects.

6. The primary purpose of medical research involving human subjects is to understand the causes, development and effects of diseases and improve preventive, diagnostic and therapeutic interventions (methods, procedures and treatments). Even the best proven interventions must be evaluated continually through research for their safety, effectiveness, efficiency, accessibility and quality.

7. Medical research is subject to ethical standards that promote and ensure respect for all human subjects and protect their health and rights.

8. While the primary purpose of medical research is to generate new knowledge, this goal can never take precedence over the rights and interests of individual research subjects.

9. It is the duty of physicians who are involved in medical research to protect the life, health, dignity, integrity, right to self-determination, privacy, and confidentiality of personal information of research subjects. The responsibility for the protection of research subjects must always rest with the physician or

other health care professionals and never with the research subjects, even though they have given consent.

10. Physicians must consider the ethical, legal and regulatory norms and standards for research involving human subjects in their own countries as well as applicable international norms and standards. No national or international ethical, legal or regulatory requirement should reduce or eliminate any of the protections for research subjects set forth in this Declaration.

11. Medical research should be conducted in a manner that minimises possible harm to the environment.

12. Medical research involving human subjects must be conducted only by individuals with the appropriate ethics and scientific education, training and qualifications. Research on patients or healthy volunteers requires the supervision of a competent and appropriately qualified physician or other health care professional.

13. Groups that are underrepresented in medical research should be provided appropriate access to participation in research.

14. Physicians who combine medical research with medical care should involve their patients in research only to the extent that this is justified by its potential preventive, diagnostic or therapeutic value and if the physician has good reason to believe that participation in the research study will not adversely affect the health of the patients who serve as research subjects.

15. Appropriate compensation and treatment for subjects who are harmed as a result of participating in research must be ensured.

Risks, Burdens and Benefits

16. In medical practice and in medical research, most interventions involve risks and burdens.

Medical research involving human subjects may only be conducted if the importance of the objective outweighs the risks and burdens to the research subjects.

17. All medical research involving human subjects must be preceded by careful assessment of predictable risks and burdens to the individuals and groups involved in the research in comparison with foreseeable benefits to them and to other individuals or groups affected by the condition under investigation.

Measures to minimise the risks must be implemented. The risks must be continuously monitored, assessed and documented by the researcher.

18. Physicians may not be involved in a research study involving human subjects unless they are confident that the risks have been adequately assessed and can be satisfactorily managed.

When the risks are found to outweigh the potential benefits or when there is conclusive proof of definitive outcomes, physicians must assess whether to continue, modify or immediately stop the study.

Vulnerable Groups and Individuals

19. Some groups and individuals are particularly vulnerable and may have an increased likelihood of being wronged or of incurring additional harm.

All vulnerable groups and individuals should receive specifically considered protection.

20. Medical research with a vulnerable group is only justified if the research is responsive to the health needs or priorities of this group and the research cannot be carried out in a non-vulnerable group. In addition, this group should stand to benefit from the knowledge, practices or interventions that result from the research.

Scientific Requirements and Research Protocols

21. Medical research involving human subjects must conform to generally accepted scientific principles, be based on a thorough knowledge of the scientific literature, other relevant sources of information, and adequate laboratory and, as appropriate, animal experimentation. The welfare of animals used for research must be respected.

22. The design and performance of each research study involving human subjects must be clearly described and justified in a research protocol.

The protocol should contain a statement of the ethical considerations involved and should indicate how the principles in this Declaration have been addressed. The protocol should include information regarding funding, sponsors, institutional affiliations, potential conflicts of interest, incentives for subjects and information regarding provisions for treating and/or compensating subjects who are harmed as a consequence of participation in the research study.

In clinical trials, the protocol must also describe appropriate arrangements for post-trial provisions.

Research Ethics Committees

23. The research protocol must be submitted for consideration, comment, guidance and approval to the concerned research ethics committee before the study begins. This committee must be transparent in its functioning, must be independent of the researcher, the sponsor and any other undue influence and must be duly qualified. It must take into consideration the laws and regulations of the country or countries in which the research is to be performed as well as applicable international norms and standards but these must not be allowed to reduce or eliminate any of the protections for research subjects set forth in this Declaration.

The committee must have the right to monitor ongoing studies. The researcher must provide monitoring information to the committee, especially information about any serious adverse events. No amendment to the protocol may be made without consideration and approval by the committee. After the end of the study, the researchers must submit a final report to the committee containing a summary of the study's findings and conclusions.

Privacy and Confidentiality

24. Every precaution must be taken to protect the privacy of research subjects and the confidentiality of their personal information.

Informed Consent

25. Participation by individuals capable of giving informed consent as subjects in medical research must be voluntary. Although it may be appropriate to consult family members or community leaders, no individual capable of giving informed consent may be enrolled in a research study unless he or she freely agrees.

26. In medical research involving human subjects capable of giving informed consent, each potential subject must be adequately informed of the aims, methods, sources of funding, any possible conflicts of interest, institutional affiliations of the researcher, the anticipated benefits and potential risks of the study and the discomfort it may entail, post-study provisions and any other relevant aspects of the study. The potential subject must be informed of the right to refuse to participate in the study or to withdraw consent to participate at any time without reprisal. Special attention should be given to the specific information needs of individual potential subjects as well as to the methods used to deliver the information.

After ensuring that the potential subject has understood the information, the physician or another appropriately qualified individual must then seek the potential subject's freely-given informed consent, preferably in writing. If the consent cannot be expressed in writing, the non-written consent must be formally documented and witnessed.

All medical research subjects should be given the option of being informed about the general outcome and results of the study.

27. When seeking informed consent for participation in a research study the physician must be particularly cautious if the potential subject is in a dependent relationship with the physician or may consent under duress. In such situations the informed consent must be sought by an appropriately qualified individual who is completely independent of this relationship.

28. For a potential research subject who is incapable of giving informed consent, the physician must seek informed consent from the legally authorised representative. These individuals must not be included in a research study that has no likelihood of benefit for them unless it is intended to promote the health of the group represented by the potential subject, the research cannot instead be performed with persons capable of providing informed consent, and the research entails only minimal risk and minimal burden.

29. When a potential research subject who is deemed incapable of giving informed consent is able to give assent to decisions about participation in research, the physician must seek that assent in addition to the consent of the legally authorised representative. The potential subject’s dissent should be respected.

30. Research involving subjects who are physically or mentally incapable of giving consent, for example, unconscious patients, may be done only if the physical or mental condition that prevents giving informed consent is a necessary characteristic of the research group. In such circumstances the physician must seek informed consent from the legally authorised representative. If no such representative is available and if the research cannot be delayed, the study may proceed without informed consent provided that the specific reasons for involving subjects with a condition that renders them unable to give informed consent have been stated in the research protocol and the study has been approved by a research ethics committee. Consent to remain in the research must be obtained as soon as possible from the subject or a legally authorised representative.

31. The physician must fully inform the patient which aspects of their care are related to the research. The refusal of a patient to participate in a study or the patient’s decision to withdraw from the study must never adversely affect the patient-physician relationship.

32. For medical research using identifiable human material or data, such as research on material or data contained in biobanks or similar repositories, physicians must seek informed consent for its collection, storage and/or reuse. There may be exceptional situations where consent would be impossible or impracticable to obtain for such research. In such situations the research may be done only after consideration and approval of a research ethics committee.

Use of Placebo

33. The benefits, risks, burdens and effectiveness of a new intervention must be tested against those of the best proven intervention(s), except in the following circumstances:

Where no proven intervention exists, the use of placebo, or no intervention, is acceptable; or

Where for compelling and scientifically sound methodological reasons the use of any intervention less effective than the best proven one, the use of placebo, or no intervention is necessary to determine the efficacy or safety of an intervention

and the patients who receive any intervention less effective than the best proven one, placebo, or no intervention will not be subject to additional risks of serious or irreversible harm as a result of not receiving the best proven intervention.

Extreme care must be taken to avoid abuse of this option.

Post-Trial Provisions

34. In advance of a clinical trial, sponsors, researchers and host country governments should make provisions for post-trial access for all participants who still need an intervention identified as beneficial in the trial. This information must also be disclosed to participants during the informed consent process.

Research Registration and Publication and Dissemination of Results

35. Every research study involving human subjects must be registered in a publicly accessible database before recruitment of the first subject.

36. Researchers, authors, sponsors, editors and publishers all have ethical obligations with regard to the publication and dissemination of the results of research. Researchers have a duty to make publicly available the results of their research on human subjects and are accountable for the completeness and accuracy of their reports. All parties should adhere to accepted guidelines for ethical reporting. Negative and inconclusive as well as positive results must be published or otherwise made publicly available. Sources of funding, institutional affiliations and conflicts of interest must be declared in the publication. Reports of research not in accordance with the principles of this Declaration should not be accepted for publication.

Unproven Interventions in Clinical Practice

37. In the treatment of an individual patient, where proven interventions do not exist or other known interventions have been ineffective, the physician, after seeking expert advice, with informed consent from the patient or a legally authorised representative, may use an unproven intervention if in the physician's judgement it offers hope of saving life, re-establishing health or alleviating suffering. This intervention should subsequently be made the object of research, designed to evaluate its safety and efficacy. In all cases, new information must be recorded and, where appropriate, made publicly available.
