# Supplementary material for: A novel nutritional supplement to reduce plasma homocysteine in nonpregnant women: A randomised controlled trial in The Gambia
Source: PLoS Med. 2019 Aug 13;16(8):e1002870. doi: 10.1371/journal.pmed.1002870 (PMC6691988; doi:10.1371/journal.pmed.1002870)
Supplement: S2 Appendix — (PDF) [file pmed.1002870.s002.pdf]

S2 Appendix: Determinants of plasma homocysteine as ingredients for the novel drink powder supplement

Introduction

Existing evidence suggests that women conceiving in the dry season in The Gambia have particular micronutrient deficiencies in nutrients related to one-carbon metabolism that result in a lower maternal methylation potential (1). This is in turn associated with a pattern of methylation suggestive of loss of imprinting at the gene *VTRNA2-1* (2). Our group's long-term goal is therefore to design a periconceptional nutritional intervention for women that improves the regulation of the infant epigenome by providing micronutrients in the quantity necessary for optimal one-carbon metabolism all year round. To achieve this we first require a proof-of-concept trial showing that a nutritional supplement can optimise the metabolome in non-pregnant women by correcting nutritional imbalances and increasing the methylation potential by reducing homocysteine. Should the supplement work it could be a promising candidate for future pregnancy trials investigating epigenetic outcomes.

The primary end point in the proof-of-concept trial is to reduce plasma homocysteine (Hcy), since maternal plasma Hcy is strongly inversely associated with the ratio of S-adenosyl methionine (SAM) to S-adensosyl homocysteine (SAH) (1), a measure of methylation potential (3), and inversely associated with infant methylation (4). The hypothesis here is that reducing homocysteine will help improve the methylation potential and enable one-carbon metabolic pathways to function unhindered.

Whilst the ratio of SAM:SAH is the most common and direct measure of methylation potential, there are a number of reasons why investigating plasma Hcy is preferable. Firstly, plasma Hcy is stable in EDTA at room temperature for 8 days and at -25°C for 29 years (5). This in in contrast to SAM, which rapidly converts into SAH and is strongly affected by freeze-thaw cycles. For example, unpublished data from the University of British Columbia suggests that the concentration of SAH after the second freeze-thaw cycle is 16.7% higher than after the first free-thaw cycle. Investigating Hcy is therefore more reliable, particularly when using stored samples. Secondly, Hcy assays are cheaper and more commonly available than those for SAM and SAH. Thirdly, there is already a wealth of existing evidence to show how nutritional interventions have been successful in reducing homocysteine through targeting one-carbon metabolic pathways (6–8).

In our target population, women of reproductive age of West Kiang district, we had three available datasets of plasma metabolites involved in one-carbon metabolism. We compared the nutritional

biomarker predictors of homocysteine in these three datasets with the aim of identifying the micronutrients consistently demonstrating inverse associations with homocysteine as candidates to take forwards into the supplement design stage. These analyses therefore provided an opportunity to tailor the supplement more specifically to the population in rural Gambia rather than rely on a more generic formula.

Methods

Three datasets are referred to in this analysis. Two are from the original Methyl Donors and Epigenetics (MDEG) study, the 'indicator group' and the 'main study group' led by Paula Dominguez-Salas from 2009-2012 (1,4,9). The third, termed 'MDEG-2' utilises stored samples from the Early Nutrition & Immune Development (ENID) Trial (10).

MDEG Indicator group

This was an observational study that took place in West Kiang district between July 2009 and June 2010, and written up in Dominguez-Salas *et al.* (2013) (1). The overall aim was to document the dietary intake and plasma nutrition concentrations of a cohort of non-pregnant women, with a focus on one-carbon related metabolites. This dataset therefore provided a reference for the seasonal variation in these metabolites over a year, hence referred to as the 'indicator group'.

Non-pregnant women between the ages of 18 and 45 years (mean age 31 years) were followed monthly for a year. Each month they provided a fasted 10 mL blood sample and were observed by field workers for collection of a 48-hour weighed dietary intake. This analysis utilises the blood sample data. Blood samples were collected in the field into EDTA monovettes, transported on ice and fully processed within two hours at MRC laboratories in Keneba. Samples were spun for 10 minutes at 2,750g and the plasma was removed, aliquoted and immediately stored at -80°C. A sample of the remaining red blood cells were removed, washed and also stored at -80°C. The original list of maternal 1-carbon biomarkers analysed were folate, B12, holotranscobalamin (active B12), choline, betaine, dimethylglycine (DMG), methionine, s-adenosyl methionine (SAM), s-adenosyl homocysteine (SAH), homocysteine (Hcy), riboflavin (B2), cysteine, 4-pyridoxic acid (PA), pyridoxal (PL) and pyridoxal 5'-phosphate (PLP). Throughout this analysis this collection of biomarkers is termed the **'core one-carbon biomarker'** set.

Plasma samples were shipped to the Department of Pediatrics, University of British Columbia (UBC), Canada for analysis of the following 1-carbon metabolites by liquid chromatography-tandem mass spectrometry: SAM, SAH, free choline, betaine, DMG, Hcy, methionine, cysteine and the B6 vitamers

(PA, PL, PLP). Plasma B12, active B12 and folate were analysed at UBC using an AxSyM analyser (Abbot laboratories, Chicago, IL). Riboflavin (B2) concentrations were analysed in the washed red blood cells by erythrocyte glutathione reductase activation coefficient (EGRAC) assay at MRC Human Nutrition Research laboratories, Cambridge, UK.

A sample size of 30 women per month was planned for, and to account for drop-out and those being excluded after becoming pregnant 62 women were recruited into the study. Overall between 22 and 30 blood draws were obtained each month throughout the year.

MDEG main study group

This dataset contains 167 mother-child pairs enrolled into the main MDEG cohort. Women of reproductive age (18-45 years) in West Kiang district were invited to participate and followed monthly. Those women conceiving in the peak of the rainy season (July-September 2009) and the peak of the dry season (February – April 2010) were enrolled and continued to be followed monthly until delivery. Their offspring were then followed throughout infancy. Detailed methodology and the main study results have been previously published in Dominguez-Salas *et al.* (2014) (4). Women provided a 10ml fasting venous blood sample at the point they reported their first missed menses (mean (SD) 8.6 ± 4 weeks gestation). The same set of core one-carbon biomarkers were analysed as described in the Indicator group, using the same laboratory analyses. The seasonal trends obtained from the Indicator group were then used to back-extrapolate the plasma concentrations to the time of conception. Infant DNA was obtained from a 3ml venepuncture taken between 2-8 months after delivery. In this main group study methylation at 6 MEs in the infant DNA were analysed. More recently two other published papers have also utilised the offspring DNA methylation data using the Illumina Infinium HumanMethytlation450 array (‘450k array’) (2,11).

MDEG-2

This dataset uses banked samples from the Early Nutrition & Immune Development (ENID) Trial (ISRCTN49285450) testing the effect of different nutritional supplements given to pregnant women on the immune development of their children. The trial provides a detailed bank of samples from multiple time-points across pregnancy and in infancy until 24 months of age. From this a subsample of mother-child pairs form the MDEG-2 dataset. The ENID trial protocol has been previously described in detail (10). In brief, women were randomised to four intervention groups after a positive pregnancy test at their ‘booking’ visit (the first clinic visit when pregnancy was confirmed, at approximately 13 weeks gestation): iron-folate (Fe-Fol) tablets, multiple micronutrient (MMN) tablets, a lipid-based nutritional supplement (LNS) fortified with Fe-Fol and LNS fortified with MMN. Supplementation was

taken daily from the booking visit until delivery. At the time of booking mothers provided a fasted sample of 10 mL venous blood; 7.5mL into lithium heparin monovettes and 2.6mL into an EDTA monovette. Samples were immediately placed on ice after the venepuncture and taken to the laboratory for processing within one hour. The monovettes were centrifuged at 1800 RCFs for ten minutes at 4°C and the plasma drawn off into 2mL microtubes. The microtubes were immediately frozen at -70°C. The plasma samples experienced two freeze-thaw cycles (one to sub-aliquot and one at the point of analysis). Since maternal booking blood samples were taken before the interventions were started the trial design does not affect interpretation of the maternal plasma nutritional biomarkers. The first booking visit took place in January 2010 and final child was born in February 2014.

The MDEG-2 dataset is comprised of a sub-sample of 350 mothers for the plasma biomarker analysis. In order to understand how the metabolome changes across the year samples were purposively selected to represent an even distribution by month of booking. Within each month the women with the earliest gestational age at booking (assessed by ultrasound) were selected to capture the metabolome closest to the periconceptional period.

The mother’s data is comprised of nutritional biomarkers analysed from their EDTA-treated plasma. The same biomarkers as MDEG main study and Indicator group were measured, with the addition of α-1-acid glycoprotein (AGP), serine, glycine, alanine, arginine, aspartic acid, glutamic acid, histidine, isoleucine, leucine, lysine, phenylalanine, proline, threonine, tryptophan, tyrosine and valine.

Vitamin B12 and folate were measured using an Abbott AxSYM autoanalyzer. Choline, betaine, DMG, homocysteine, folate, B6 vitamers (PA, PL, PLP) and vitamin B2 were measured using liquid chromatography-tandem mass spectrometry. The amino acids were analysed using a Hitachi L-8900 amino acid analyser. All of these biomarkers were measured at the Child and Family Research Institute at the University of British Columbia. The inflammatory marker AGP was measured using the Cobas Integra 400 plus autoanalyser at MRC The Gambia, Keneba field station.

Selection of 1-carbon biomarkers as potential predictors

We assessed the original list of biomarkers in each dataset for multicollinearity by inspecting the variance inflation factors. Amongst the B6 vitamers we dropped pyridoxic acid and pyridoxal from all datasets since they were highly correlated with pyridoxal-5’-phospahte (PLP), which is the main functional marker of vitamin B6. Since the active B12 assay did not work correctly in the MDEG-2 dataset we used plasma total B12 from all datasets instead. S-adenosyl methionine (SAM) and s-adenosyl homocysteine (SAH) from the MDEG-2 dataset had degraded over storage time and/or

freeze-thaw processing and were unusable. We dropped them from the list of potential predictors for that reason, and also because we wanted to prioritise the biomarkers that could be considered for a supplement rather than the intermediary metabolites. For the MDEG main study and the Indicator group the final list of potential predictors was therefore: B2, PLP, B12, cysteine, methionine, dimethylglycine (DMG), folate, choline and betaine. MDEG-2 used the same list but also included the amino acid panel.

Predictive models: three approaches

Since regression models are not always stable in their variable selection we wanted to compare three different methods of linear regression modelling: a two-step approach, backwards stepwise and least angle regression using Lasso. The aim was to obtain a qualitative overview in order to identify consistent patterns of predictors of homocysteine in different datasets using different variable selection approaches.

We used log Hcy as the dependent variable in all our models. We standardised and log-transformed all potential nutritional predictors to be entered into the models as independent variables. All effect sizes therefore represent the change in log Hcy for a one SD increase in the log-transformed independent variable. We forced *a priori* confounders into all models, and these varied according to the information available for each dataset (age for Indicator group; age, BMI and gestational age for MDEG main study; age, BMI, gestational age and inflammation using α-1-acid glycoprotein (AGP) for MDEG-2). The Indicator group dataset contains repeated measures on the same individual. Both the Breusch-Pagan test (for random effects versus ordinary least squares regression, $p<0.0001$) and the Hausman test (for fixed versus random effects, $p=0.2409$), confirmed the use of random effects. This longitudinal dataset, however, could only be used in the two-step approach, which allowed for the modelling of random effects. The MDEG main study and MDEG-2 datasets, being cross-sectional, could be used in all three modelling approaches.

In the two-step approach we regressed log Hcy against the nutritional predictors in univariable linear regression. Any variables with $p<0.1$ were then taken forwards to a multivariable linear regression model. In the final results we report any retained independent variables with $p<0.05$. We used the Wald test for the statistical tests in this approach. In the backwards stepwise approach we used automatic selection with $p>0.2$ as the criteria for removal from the model. Again, in the final results we only report the retained independent variables with $p<0.05$ using the Wald test. In the Lasso model the number of variables retained in the final model corresponds to the lowest Cp statistic found. No individual p values are reported and the output contains the coefficients only. Given it was not possible

to force confounders into the Lasso models we pre-adjusted log Hcy by regressing it against the confounders prior to model entry.

We ran the three models using all the data available ('combined season' model), but then also stratified the models by peak of the dry season (February – April) and the peak of the rainy season (July – September). This was because we particularly wanted to run the supplementation trial in the dry season. Table 1 summarises the different datasets we used, detailing the list of potential nutritional predictors, the confounders, the type of statistical models used and the samples sizes.

Table 1: Summary of datasets and models used

Dataset	Total N	Dry Season N	Rainy Season N	Biomarkers included in model	A priori confounders forced into all models	Models used	Notes
**Indicator group**	48 non-pregnant women with 288 total observations	34 women with 79 observations	28 women with 63 observations	Hcy, B2, PLP, B12, folate, methionine, choline, betaine, DMG, cysteine	Age	Linear regression with random effects. 2-step approach only	Non-pregnant women, roughly 30 followed for one year, longitudinal study with monthly blood samples (1).
**MDEG main study**	167 pregnant women	83	84	Hcy, B2, PLP, B12, folate, methionine, choline, betaine, DMG, cysteine	Maternal age, BMI, gestational age	Ordinary linear regression. 2-step, backwards stepwise and Lasso	Cross-sectional data, pregnant women selected from peak of rainy and peak of dry season (4).
**MDEG-2**	350 pregnant women	87	100	Hcy, B2, PLP, B12, folate, methionine, choline, betaine, DMG, cysteine, Asp, Thr, Ser, Glu, Gly, Ala, Val, Ile, Leu, Tyr, Phe, Lys, His, Arg, Pro	Maternal age, BMI, gestational age, inflammation (AGP)	Ordinary linear regression. 2-step, backwards stepwise and Lasso	Cross sectional data, pregnant women selected from each month of year.

Abbreviations: Hcy, homocysteine; PLP, pyridoxal 5'-phosphate; DMG, dimethylglycine; BMI, body mass index; Asp, aspartic acid; Thr, threonine; Ser, serine; Glu, glutamic acid; Gly, glycine; Ala, alanine; Val, valine; Ile, isoleucine; Leu, leucine; Tyr, tyrosine; Phe, phenylalanine; Lys, lysine; His, histidine; Arg, arginine; Pro, proline.

Results

Tables 2, 3 and 4 detail the predictors of Hcy retained in final multivariable models in the MDEG main study, MDEG-2 and Indicator group datasets respectively. A summary of the retained coefficients in all models is provided in Figure 1.

There were four predictors that were consistently inversely associated with Hcy in most model approaches and seasons: folate, B2, B12 and betaine. Of these folate had the strongest effect sizes, particularly in the dry season. Cysteine was positively associated with Hcy in all models. DMG was also positively associated with Hcy in the combined and dry season models.

The pattern for choline and PLP was inconsistent. In several models they were not retained as predictors, and where they did feature the direction of the association varied. Choline was inversely associated with Hcy in the MDEG-2 Lasso dry and rainy season models, yet was positively associated in the MDEG main study Lasso models in all seasons. PLP was positively associated with Hcy in the MDEG-2 dataset but showed different directions of association by season in the MDEG main study dataset.

The amino acids were only measured in the MDEG-2 dataset. Glycine was a positive predictor of Hcy in the combined models but was not retained when models were stratified by season. None of the other amino acids showed a consistent pattern, and when they were retained in the final models they had small effect sizes.

Table 2: Nutritional biomarker independent predictors of homocysteine in MDEG main study dataset

Seasonal Model	Statistical approach*	N	R^2	Positive predictors‡ (β, 95%CI, p value)		Negative predictors‡ (β, 95%CI, p value)	
Combined Seasons	2-step	150	0.582	Cysteine	0.14 (0.10, 0.18), p<0.001	Folate	-0.12 (-0.16, -0.09), p<0.001
				DMG	0.05 (0.01, 0.09), p=0.015	B2	-0.05 (-0.08, -0.01), p=0.009
	Backwards Stepwise	149	0.610	Cysteine	0.16 (0.12, 0.20), p<0.001	Folate	-0.14 (-0.17, -0.10), p<0.001
						B2	-0.04 (-0.07, 0.00), p=0.029
						B12	-0.05 (-0.09, -0.02), p=0.005
	Lasso	152	0.422	Cysteine	0.14	Folate	-0.11
				DMG	0.04	Betaine	-0.08
				Choline	0.04	B2	-0.04
				Methionine	0.02	B12	-0.03
						PLP	-0.01
Dry season (Feb-Apr)	2-step	79	0.575	Cysteine	0.16 (0.10, 0.22), p<0.001	Folate	-0.17 (-0.23, -0.11), p<0.001
						B2	-0.07 (-0.13, -0.01), p=0.018
	Backwards Stepwise	79	0.542	Cysteine	0.16 (0.10, 0.23), p<0.001	Folate	-0.18 (-0.24, -0.12), p<0.001
	Lasso	80	0.423	Cysteine	0.13	Folate	-0.11
				DMG	0.05	B2	-0.06
				Choline	0.06	B12	-0.02
				Methionine	0.03	Betaine	-0.11
						PLP	-0.02
Rainy season (Jul-Oct)	2-step	77	0.622	Cysteine	0.13 (0.08, 0.17), p<0.001	None	
	Backwards stepwise	70	0.732	Cysteine	0.17 (0.12, 0.21), p<0.001	Folate	-0.10 (-0.14, -0.06), p<0.001
						B12	-0.05 (-0.09, -0.01), p=0.010
	Lasso	72	0.504	Cysteine	0.14	Folate	-0.09
				Choline	0.04	Betaine	-0.05
				DMG	0.03	B2	-0.02
				PLP	0.03	B12	-0.04

*Lasso model fitted to variables demonstrating the smallest Cp value. Note no confidence intervals are reported for Lasso models.

ǂPredictors are adjusted for maternal age, gestational and BMI. Only variables with a final $p<0.05$ reported.

‡β Coefficients represent the change in log homocysteine for a one SD increase in the log-transformed independent variable.

Abbreviations: CI, confidence interval; DMG, dimethylglycine; PLP, pyridoxal 5’-phosphate

Table 3: Nutritional biomarker independent predictors of homocysteine in MDEG-2 dataset

Seasonal Model	Statistical approach*	N	R^2	Positive predictors[‡] (β, 95%CI, p value)		Negative predictors[‡] (β, 95%CI, p value)	
Year-round	2-step	339	0.588	Cysteine	0.21 (0.18, 0.24), p<0.001	Folate	-0.10 (-0.13, -0.08), p<0.001
				Glycine	0.05 (0.02, 0.08), p<0.001	B12	-0.10, (-0.13, -0.08), p<0.001
				DMG	0.03 (0.01, 0.06), p=0.019	Betaine	-0.07 (-0.10, -0.04), p<0.001
	Backwards Stepwise	338	0.610	Cysteine	0.2 (0.18, 0.23), p<0.001	Folate	-0.10 (-0.13, -0.08), p<0.001
				Glycine	0.06 (0.04, 0.09), p<0.001	B12	-0.10 (-0.12, -0.07), p<0.001
				DMG	0.04 (0.01, 0.06), p=0.004	Betaine	-0.07 (-0.10, -0.04), p<0.001
				Proline	0.04 (0.01, 0.07), p=0.009	B2	-0.05 (-0.07, -0.02), p<0.001
				PLP	0.04 (0.00, 0.07), p=0.027		
				Aspartate	0.03 (0.00, 0.06), p=0.046		
	Lasso	342	0.540	Cysteine	0.18	Folate	-0.09
				Glycine	0.06	B12	-0.08
				PLP	0.04	Betaine	-0.06
				DMG	0.03	B2	-0.04
				Proline	0.03	Tyrosine	-0.02
				Glutamate	0.02	Threonine	-0.02
				Aspartate	0.02	Alanine	-0.01
				Isoleucine	0.01	Methionine	-0.01
				Serine	0.01		
Dry season (Feb-Apr)	2-step	82	0.470	Cysteine	0.18 (0.13, 0.23), p<0.001	B12	-0.08 (-0.13, -0.03), p=0.001
						Betaine	-0.07 (-0.12, -0.03), p=0.003
	Backwards Stepwise	81	0.624	Cysteine	0.18 (0.13, 0.23), p<0.001	Betaine	-0.08 (-0.13, -0.04), p<0.001
				PLP	0.05 (0.01, 0.10), p=0.021	Folate	-0.08 (-0.13, -0.02), p=0.008
				Aspartate	0.05 (0.00, 0.10), p=0.042	B12	-0.07 (-0.12, -0.03), p=0.003
						B2	-0.05 (-0.10, -0.01), p=0.013
						Threonine	-0.04 (-0.08, 0.00), p=0.045
	Lasso	85	0.572	Cysteine	0.16	Betaine	-0.06
				PLP	0.07	Folate	-0.06
				Proline	0.04	B12	-0.06
				Serine Glycine	0.03	Choline	-0.04

Seasonal Model	Statistical approach*	N	R^2	Positive predictors‡ (β, 95%CI, p value)		Negative predictors‡ (β, 95%CI, p value)	
				Aspartate	0.02	B2	-0.03
				DMG	0.02	Threonine	-0.03
					0.01		
Rainy season (Jul-Oct)	2-step	98	0.636	Cysteine	0.23 (0.17, 0.29), p<0.001	B12	-0.09 (-0.14, -0.04), p<0.001
						Folate	-0.08 (-0.12, -0.03), p=0.001
						B2	-0.06 (-0.11, -0.01), p=0.022
	Backwards stepwise	98	0.700	Cysteine	0.24 (0.18, 0.29), p<0.001	Leucine	-0.10 (-0.19, -0.01), p=0.035
				Valine	0.11 (0.03, 0.19), p=0.005	B12	-0.08, (-0.12, -0.04), p<0.001
				Proline	0.07 (0.01, 0.13), p=0.028	Folate	-0.07 (-0.11, -0.03), p=0.002
				Aspartate	0.04, p=0.022	Alanine	-0.07 (-0.12, -0.01), p=0.028
						B2	-0.05 (-0.10, 0.00), p=0.036
	Lasso	98	0.529	Cysteine	0.18	B12	-0.06
				Glutamate	0.03	Folate	-0.06
				Proline	0.02	B2	-0.04
				Aspartate	0.01	Betaine	-0.02
				Valine	0.01	Tyrosine	-0.02
						Histidine	-0.01
						Choline	-0.01

*Lasso model fitted to variables demonstrating the smallest Cp value. Note no confidence intervals are reported for Lasso models.

ᵻPredictors are adjusted for maternal age, BMI, gestational age and inflammation (AGP). Only variables with a final p<0.05 reported.

‡β Coefficients represent the change in log homocysteine for a one SD increase in the log-transformed independent variable.

Abbreviations: AGP, Alpha-1-acid glycoprotein; CI, confidence interval; DMG, dimethylglycine; PLP, pyridoxal 5’-phosphate

Table 4: Nutritional biomarker independent predictors of homocysteine in the Indicator group dataset

Seasonal Model	No. observations	No. women	Overall R2*	Positive predictors[ǂ] (β[‡], 95% CI, p value)		Negative predictors[ǂ] (β[‡], 95% CI, p value)	
**Combined seasons**	288	48	0.306	Cysteine	0.14 (0.11, 0.17), p<0.001	Folate	-0.11 (-0.13, -0.08), p<0.001
				DMG	0.08 (0.04, 0.12), p<0.001	Betaine	-0.05 (-0.08, -0.02), p=0.003
**Dry (Feb-Apr)**	79	34	0.295	Cysteine	0.17 (0.12, 0.21), p<0.001	Folate	-0.15 (-0.22, -0.08), p<0.001
**Rainy (Jul-Oct)**	63	28	0.549	Cysteine	0.16 (0.11, 0.22), p<0.001	Folate	-0.13 (-0.18, -0.07), p<0.001
						B12	-0.09 (-0.16, -0.02), p=0.009
						B2	-0.09 (-0.17, -0.01), p=0.026

*Two-step linear regression using random effects

[ǂ]Predictors are those variables retained in the multivariable model with p<0.05, adjusted for age.

[‡]β Coefficients represent the change in log homocysteine for a one SD increase in the log-transformed independent variable.

Abbreviations: CI, confidence interval; DMG, dimethylglycine

Figure 1: Summary of retained coefficients of multivariable linear regression models in three datasets, by season*.

	Combined seasons						
Biomarker	MDEG2 2-step	MDEG2 Stepwise	MDEG2 LASSO	MDEG1 2-step	MDEG1 Stepwise	MDEG1 LASSO	Indicator 2-step
Folate	-0.1	-0.1	-0.09	-0.12	-0.14	-0.11	-0.11
B2		-0.05	-0.04	-0.05	-0.04	-0.04	
PLP		0.04	0.04			-0.01	
B12	-0.1	-0.1	-0.08		-0.05	-0.03	
Betaine	-0.07	-0.07	-0.06			-0.08	-0.05
Choline						0.04	
Methionine			-0.01			0.02	
Cysteine	0.21	0.2	0.18	0.14	0.16	0.14	0.14
DMG	0.03	0.04	0.03	0.05		0.04	0.08
Glycine	0.05	0.06	0.06				
Glutamate			0.02				
Proline		0.04	0.03				
Aspartate		0.03	0.02				
Tyrosine			-0.02				
Alanine			-0.01				
Isoleucine			0.01				
Serine			0.01				
Threonine			-0.02				

	Dry season						
Biomarker	MDEG2 2-step	MDEG2 Stepwise	MDEG2 LASSO	MDEG1 2-step	MDEG1 Stepwise	MDEG1 LASSO	Indicator 2-step
Folate		-0.08	-0.06	-0.17	-0.18	-0.11	-0.15
B2		-0.05	-0.03	-0.07		-0.06	
PLP		0.05	0.07			-0.02	
B12	-0.08	-0.07	-0.06			-0.02	
Betaine	-0.07	-0.08	-0.06			-0.11	
Choline			-0.04			0.06	
Methionine						0.03	
Cysteine	0.18	0.18	0.16	0.16	0.16	0.13	0.17
DMG			0.01			0.05	
Glycine			0.02				
Glutamate							
Proline			0.04				
Aspartate		0.05	0.02				
Tyrosine							
Alanine							
Isoleucine							
Serine			0.03				
Threonine		-0.04	-0.03				

	Rainy season						
Biomarker	MDEG2 2-step	MDEG2 Stepwise	MDEG2 LASSO	MDEG1 2-step	MDEG1 Stepwise	MDEG1 LASSO	Indicator 2-step
Folate	-0.08	-0.07	-0.06		-0.1	-0.09	-0.13
B2	-0.06	-0.05	-0.04			-0.02	-0.09
PLP						0.03	
B12	-0.09	-0.08	-0.06		-0.05	-0.04	-0.09
Betaine			-0.02			-0.05	
Choline			-0.01			0.04	
Methionine						0.02	
Cysteine	0.23	0.24	0.18	0.13	0.17	0.14	0.16
DMG						0.03	
Glycine							
Glutamate			0.03				
Proline		0.07	0.02				
Aspartate		0.04	0.01				
Tyrosine			-0.02				
Alanine		-0.07					
Histidine			-0.01				
Valine		0.11	0.01				
Leucine		-0.1					

***Note:** Coefficients represent change in log homocysteine for each SD increase in the log-transformed independent variable. Only variables with $p<0.05$ shown. Those inversely associated with homocysteine are shaded in green, and those positively associated are shaded in red.

Abbreviations: DMG, dimethylglycine; MDEG1, MDEG main study

Discussion

In order to assess which nutritional components we could consider for a supplement to reduce plasma homocysteine we analysed three different datasets using different linear regression approaches to see which nutritional biomarkers were consistently inversely associated with Hcy. This approach built up an overview of the most reliable predictors of homocysteine. We found that folate, B12, B2 and betaine were the most consistent negative predictors of Hcy.

Negative predictors of homocysteine

The findings that plasma folate, B12, B2 and betaine were inversely associated with Hcy are consistent with the literature (12–15). Supplementation with these components have been successful in previous trials with the aim of reducing homocysteine (6,16–19).

Hcy can be metabolised via a process involving remethylation or through the transsulfuration pathway. In the former, Hcy accepts a methyl group to form methionine (20), which can then in turn be condensed with ATP to form S-adenosyl methionine, a methyl donor involved in numerous transmethylation reactions. The remethylation of Hcy to methionine uses two distinct pathways. The major one is the vitamin B12- dependent reaction involving folate metabolic pathways (21), chiefly the donation of a methyl group from N^5-methyl tetrahydrofolate ('methyl-THF'). The alternative pathway for the methylation of Hcy, predominantly used in the liver and kidneys, uses the methyl group from betaine, a product formed through the oxidation of choline (22,23). In the transsulfuration pathway Hcy is metabolised through its irreversible degradation to cystathionine and cysteine, requiring PLP (vitamin B6) (21).

Dietary folates and folic acid therefore contribute to the removal of homocysteine via methyl-THF. They are first reduced to form tetrahydrofolate (THF), which is in turn is reduced to methylene-THF, then to methyl-THF. This is the form that donates its methyl group to Hcy using vitamin B12. In human plasma and the cytosol the predominant form of vitamin B12 is methylcobalamin (24). This is the form that is used as a coenzyme for methionine synthase, the enzyme responsible for adding the methyl group from 5-methyl-THF to Hcy to form methionine.

The above metabolic descriptions explain why folate, betaine and B12 are required to metabolise Hcy and remove it from the system. In order to understand where B2 fits in we need to re-visit the step where dietary folates are converted to methylene-THF. B2 is required as a precursor to flavin mononucleotide (FMN), a reaction catalysed by riboflavin kinase. FMN is converted to flavin adenine

dinucleotide (FAD) by FAD synthase (25,26). FAD is a cofactor required by methylenetetrahydrofolate reductase (MTHFR) to reduce methylene-THF to methyl-THF (3).

Threonine was retained as a negative predictor of Hcy in some models, as was alanine in the analyses excluding cysteine and DMG. The relationship between plasma Hcy and amino acids is not well documented in the literature. However, in a study investigating plasma metabolites in healthy controls no association between Hcy and amino acids was found (27). Without further justification from the literature it is not possible to say whether these are genuine or chance findings of associations, especially as amino acids were only measured in one of our datasets.

Positive predictors of homocysteine

Cysteine was the strongest positive predictor of Hcy, which was to be expected given that Hcy can be converted into cystathionine by Cystathionine β-synthase (CBS) and then onto cysteine by Cystathionine γ lyase (21). The positive association of DMG with homocysteine can be explained by the reaction catalysed by betaine-homocysteine methyltransferase (BHMT), which methylates Hcy using a methyl group from betaine and in the process forms DMG (28). Thus describing cysteine and DMG as 'positive predictors' can be misleading since they are by-products of Hcy catabolism.

There were no strong associations between methionine and Hcy, most likely explained by its position on a circular metabolic pathway and therefore it be viewed as both a substrate for Hcy production and as a by-product of Hcy catabolism. It can be seen as a substrate for Hcy production since it is condensed with ATP to form SAM, which is then demethylated to SAH, which in turn is hydrolysed to form Hcy and adenosine (21,29). We can also view methionine as the product resulting from Hcy catabolism through Hcy remethylation using the methyl group from 5-methyl-THF or betaine. Linear regression models are not designed to model such circular pathways and therefore it is perhaps not surprising that no clear associations between methionine and Hcy can be identified. We might only expect homocysteine to significantly rise in response to a methionine load (e.g. straight after a protein-rich meal), whereas the samples in these datasets are fasted blood samples.

Given that choline is oxidised to betaine (30), and that in our datasets betaine is strongly inversely associated with Hcy, we had also expected to see an inverse association between choline and homocysteine. Whilst this was the case in the MDEG-2 dataset, in the MDEG main study choline had a positive association with Hcy. Despite seeming counter intuitive, this positive association has been found previously in pregnant women (12). Molloy et al. (2005) hypothesise that increased fetal requirements for choline during pregnancy upregulates choline production via phosphatidylcholine

(PC) synthesis (12). To produce one molecule of PC using the PEMT pathway three methyl groups from SAM are required. Once SAM donates its methyl group it forms SAH, which is hydrolysed to Hcy (31), thereby increasing Hcy concentrations. It is therefore unlikely that choline is associated with increased Hcy in a causal relationship *per se,* but that the association is due to the physiological adaptation in pregnancy to increased endogenous production of choline. This remains speculative since we cannot distinguish between dietary and endogenous sources of choline in our plasma samples, nor was the direction of the association between choline and Hcy consistent within our datasets.

Vitamin B6 (in the active form of PLP) is required to reduce THF to methylene-THF, and is also a cofactor in the transsulfuration pathway converting homocysteine to cysteine (21). If PLP was to be retained in the models at all we would have expected it to be as a negative predictor, considering its role in pathways responsible for Hcy's remethylation and transsulfuration. Indeed, B6 has been inversely associated with Hcy in previous studies (14,32,33). However, there are also several studies that show that PLP has no effect on fasting Hcy levels (13,34,35). It is more likely that the effect of PLP will be seen in reducing Hcy after a methionine load (21). It is biologically very unlikely that PLP would cause an increase in plasma Hcy, and its retention in some of the models as a positive predictor is not easily explained.

The positive association between glycine and Hcy could be explained by the reaction catalysed by glycine N-methyltransferase (GNMT). In this reaction glycine accepts a methyl group from SAM to form SAH and sarcosine. This reaction occurs especially when SAM concentrations are high (19). The production of SAH from this reaction would therefore contribute to higher Hcy through the hydrolysis of SAH to Hcy. Surprisingly, serine was not retained in many models at all, and where it was retained the effect size was very small. Given that serine is used in the transsulfuration pathway to catabolise Hcy, and also used as a 1-carbon donor at the stage of converting THF to methylene-THF through the action of Serine Hydroxymethyltransferase and PLP (36), we might have expected it to have an inverse association with Hcy.

The unexplained variability in plasma Hcy could perhaps be partially explained by the effect of SNPs in enzymes involved in one-carbon metabolism (e.g. MTHFR, CBS) on homocysteine levels. The notion that relevant SNPs influence plasma Hcy levels has been well documented in other populations (37–43). However, the effect of these genetic variants on total plasma Hcy variability is likely to be less significant than the nutritional biomarker predictors, with the former often explaining less than 6% of total Hcy variability (37,42). There are also several other potential predictors of Hcy which we did not measure (e.g. fat-free mass, renal function, estradiol and creatinine concentrations, caffeine consumption, smoking, alcohol intake etc.(14,35)). The overall aim of these analyses was to identify

nutritional inputs that could be modified through a supplement, rather than an exercise in identifying all potential predictors of Hcy. The high proportion of Hcy variability explained by the profile of nutritional predictors we considered gave us confidence that a nutritional approach to modifying Hcy has promising potential.

Limitations

All linear regression approaches have their own set of limitations in the way they select and report the effect of a sub-set of explanatory variables (44–47). The potential differences that the type of linear regression approach can make to variable selection is part of the reason we chose to compare three different approaches. In a traditional research study on predictors of homocysteine this would clearly be open to criticisms of multiple testing and an incoherent statistical approach. However, for a supplement design aim it was useful to assess to what extent similar trends emerged across multiple datasets and linear regression approaches that could help identify consistent negative predictors of Hcy.

The datasets we investigated carry the limitations of utilising cross-sectional plasma information. Whilst useful for identifying predictors of Hcy they cannot go much further in guiding the doses of ingredients to include in a supplement, nor what the expected effect of a supplement might be on plasma Hcy. To achieve this would require the integration of dietary intake data and kinetic data that could be used in more formal pharmacodynamics approaches, which go beyond the scope of this study. A plasma sample taken at one point in time can provide clues as to what some of the underlying determinants of plasma Hcy are, but without further clinical tests it can be difficult to disentangle the components. For example, a high Hcy concentration could be due to increased transmethylation, decreased transsulfuration, decreased remethylation, decreased uptake of Hcy by the kidney, genetic defects in key enzymes, or a combination of these factors (21,29,48). Thus the utility of these analyses is constrained to selection of potential ingredients to take forwards to the supplement design stage. The outputs of this analysis may therefore be seen as more qualitative rather than quantitative, but still help to design a supplement that is better tailored to the West Kiang population rather than rely solely on a generic, commercially available supplement.

Conclusion

In the context of West Kiang, the four nutritional biomarkers consistently inversely associated with plasma Hcy were betaine, B12, B2 and folate. These were consistent with the literature. We had expected vitamin B6 and choline to also be inversely associated with Hcy, but since this was not supported by our data we dropped them from the list of potential supplement ingredients. We therefore took betaine, B12, B2 and folate forwards into the supplement design stage.

References

1. Dominguez-Salas P, Moore SE, Cole D, da Costa K-A, Cox SE, Dyer RA, Fulford AJC, Innis SM, Waterland RA, Zeisel SH, et al. DNA methylation potential: dietary intake and blood concentrations of one-carbon metabolites and cofactors in rural African women. Am J Clin Nutr. 2013;97:1217–27.

2. Silver MJ, Kessler NJ, Hennig BJ, Dominguez-Salas P, Laritsky E, Baker MS, Coarfa C, Hernandez-Vargas H, Castelino JM, Routledge MN, et al. Independent genomewide screens identify the tumor suppressor VTRNA2-1 as a human epiallele responsive to periconceptional environment. Genome Biol. 2015;16:118.

3. Mason JB. Biomarkers of Nutrient Exposure and Status in One-Carbon (Methyl) Metabolism. J Nutr. 2003;133:941S–947.

4. Dominguez-Salas P, Moore SE, Baker MS, Bergen AW, Cox SE, Dyer R a., Fulford AJ, Guan Y, Laritsky E, Silver MJ, et al. Maternal nutrition at conception modulates DNA methylation of human metastable epialleles. Nat Commun. 2014;5:3746.

5. Hustad S, Eussen S, Midttun Ø, Ulvik A, van de Kant PM, Mørkrid L, Gislefoss R, Ueland PM. Kinetic modeling of storage effects on biomarkers related to B vitamin status and one-carbon metabolism. Clin Chem. 2012;58:402–10.

6. Homocysteine Lowering Trialists' Collaboration. Lowering blood homocysteine with folic acid based supplements: meta-analysis of randomised trials. BMJ. 1998;316:894–8.

7. Huang T, Zheng J, Chen Y, Yang B, Wahlqvist ML, Li D. High consumption of Ω-3 polyunsaturated fatty acids decrease plasma homocysteine: a meta-analysis of randomized, placebo-controlled trials. Nutrition. 2011;27:863–7.

8. McRae MP. Betaine supplementation decreases plasma homocysteine in healthy adult participants: a meta-analysis. J Chiropr Med. 2013;12:20–5.

9. Dominguez-Salas P. Epigenesis in humans: Can maternal methyl-donor-deficient diets induce epigenetic alterations in their offspring? PhD Thesis. London School of Hygiene and Tropical Medicine; 2012.

10. Moore SE, Fulford AJ, Darboe MK, Jobarteh ML, Jarjou LM, Prentice AM. A randomized trial to investigate the effects of pre-natal and infant nutritional supplementation on infant immune development in rural Gambia: the ENID trial: Early Nutrition and Immune Development. BMC Pregnancy Childbirth. 2012;12:107.

11. Hernandez-Vargas H, Castelino J, Silver MJ, Dominguez-Salas P, Cros M-P, Durand G, Le Calvez-Kelm F, Prentice AM, Wild CP, Moore SE, et al. Exposure to aflatoxin B1 in utero is associated with DNA methylation in white blood cells of infants in The Gambia. Int J Epidemiol. 2015;44:1238–48.

12. Molloy AM, Mills JL, Cox C, Daly SF, Conley M, Brody LC, Kirke PN, Scott JM, Ueland PM. Choline and homocysteine interrelations in umbilical cord and maternal plasma at delivery. Am J Clin Nutr. 2005;82:836–42.

13. Holm PI, Ueland PM, Vollset SE, Midttun Ø, Blom HJ, Keijzer MBAJ, den Heijer M. Betaine and folate status as cooperative determinants of plasma homocysteine in humans. Arterioscler Thromb Vasc Biol. 2005;25:379–85.

14. Jacques PF, Bostom AG, Wilson PW, Rich S, Rosenberg IH, Selhub J. Determinants of plasma total homocysteine concentration in the Framingham Offspring cohort. Am J Clin Nutr.

2001;73:613–21.

15. van Beynum IM, den Heijer M, Thomas CM, Afman L, Oppenraay-van Emmerzaal D, Blom HJ. Total homocysteine and its predictors in Dutch children. Am J Clin Nutr. 2005;81:1110–6.

16. Steenge GR, Verhoef P, Katan MB. Betaine supplementation lowers plasma homocysteine in healthy men and women. J Nutr. 2003;133:1291–5.

17. Olthof MR, van Vliet T, Verhoef P, Zock PL, Katan MB. Effect of homocysteine-lowering nutrients on blood lipids: results from four randomised, placebo-controlled studies in healthy humans. PLoS Med. 2005;2:e135.

18. Dawson SL, Bowe SJ, Crowe TC. A combination of omega-3 fatty acids, folic acid and B-group vitamins is superior at lowering homocysteine than omega-3 alone: A meta-analysis. Nutr Res. 2016;36:499–508.

19. Olthof MR, Verhoef P. Effects of betaine intake on plasma homocysteine concentrations and consequences for health. Curr Drug Metab. 2005;6:15–22.

20. Robinson JL, Bertolo RF. The Pediatric Methionine Requirement Should Incorporate Remethylation Potential and Transmethylation Demands. Adv Nutr. 2016;7:523–34.

21. Selhub J. Homocysteine metabolism. Annu Rev Nutr. 1999;19:217–46.

22. Velzing-Aarts F V, Holm PI, Fokkema MR, van der Dijs FP, Ueland PM, Muskiet FA. Plasma choline and betaine and their relation to plasma homocysteine in normal pregnancy. Am J Clin Nutr. 2005;81:1383–9.

23. McKeever MP, Weir DG, Molloy A, Scott JM. Betaine-homocysteine methyltransferase: organ distribution in man, pig and rat and subcellular distribution in the rat. Clin Sci. 1991;81:551–6.

24. Green R, Miller JW. Vitamin B12. In: ZempleniJ, Rucker R, McCormick D, Suttie J, editors. Handbook of Vitamins. 4th ed. Boca Ralton, FL: Taylor & Francis Group; 2007.

25. Hustad S, McKinley MC, McNulty H, Schneede J, Strain JJ, Scott JM, Ueland PM. Riboflavin, Flavin Mononucleotide, and Flavin Adenine Dinucleotide in Human Plasma and Erythrocytes at Baseline and after Low-Dose Riboflavin Supplementation. Clin Chem. 2002;48:1571–7.

26. Hustad S, Schneede J, Ueland PM. Madame Curie Bioscience Database [Internet]. Austin (TX): Landes Bioscience; 2013.

27. Hong SY, Yang DH, Chang SK. The relationship between plasma homocysteine and amino acid concentrations in patients with end-stage renal disease. J Ren Nutr. 1998;8:34–9.

28. Ueland PM, Holm PI, Hustad S. Betaine: a key modulator of one-carbon metabolism and homocysteine status. Clin Chem Lab Med. 2005;43:1069–75.

29. Stipanuk MH. Sulfur amino acid metabolism: pathways for production and removal of homocysteine and cysteine. Annu Rev Nutr. 2004;24:539–77.

30. Ueland PM. Choline and betaine in health and disease. J Inherit Metab Dis. 2011;34:3–15.

31. Pajares MA, Pérez-Sala D. Betaine homocysteine S-methyltransferase: just a regulator of homocysteine metabolism? Cell Mol Life Sci. 2006;63:2792–803.

32. Selhub J, Jacques PF, Wilson PW, Rush D, Rosenberg IH. Vitamin status and intake as primary determinants of homocysteinemia in an elderly population. JAMA. 1993;270:2693–8.

33. Siri PW, Verhoef P, Kok FJ. Vitamins B6, B12, and folate: association with plasma total

homocysteine and risk of coronary atherosclerosis. J Am Coll Nutr. 1998;17:435–41.

34. Andersson A, Brattström L, Israelsson B, Isaksson A, Hamfelt A, Hultberg B. Plasma homocysteine before and after methionine loading with regard to age, gender, and menopausal status. Eur J Clin Invest. 1992;22:79–87.

35. Dierkes J, Jeckel A, Ambrosch A, Westphal S, Luley C, Boeing H. Factors explaining the difference of total homocysteine between men and women in the European Investigation Into Cancer and Nutrition Potsdam study. Metabolism. 2001;50:640–5.

36. Davis SR, Stacpoole PW, Williamson J, Kick LS, Quinlivan EP, Coats BS, Shane B, Bailey LB, Gregory JF. Tracer-derived total and folate-dependent homocysteine remethylation and synthesis rates in humans indicate that serine is the main one-carbon donor. Am J Physiol Endocrinol Metab. 2004;286:E272-9.

37. van Meurs JB, Pare G, Schwartz SM, Hazra A, Tanaka T, Vermeulen SH, Cotlarciuc I, Yuan X, Mälarstig A, Bandinelli S, et al. Common genetic loci influencing plasma homocysteine concentrations and their effect on risk of coronary artery disease. Am J Clin Nutr. 2013;98:668–76.

38. Cotlarciuc I, Malik R, Holliday EG, Ahmadi KR, Paré G, Psaty BM, Fornage M, Hasan N, Rinne PE, Ikram MA, et al. Effect of genetic variants associated with plasma homocysteine levels on stroke risk. Stroke. 2014;45:1920–4.

39. Zhu Y, Zhu R-X, He Z-Y, Liu X, Liu H-N. Association of MTHFR C677T with total homocysteine plasma levels and susceptibility to Parkinson’s disease: a meta-analysis. Neurol Sci. 2015;36:945–51.

40. Nienaber-Rousseau C, Ellis SM, Moss SJ, Melse-Boonstra A, Towers GW. Gene–environment and gene–gene interactions of specific MTHFR, MTR and CBS gene variants in relation to homocysteine in black South Africans. Gene. 2013;530:113–8.

41. Stefano V, Dekou V, Nicaud V, Chasse JF, London J, Stansbie D, Humphries SE, Gudnason V. Linkage disequilibrium at the cystathionine beta synthase (CBS) locus and the association between genetic variation at the CBS locus and plasma levels of homocysteine. Ann Hum Genet. 1998;62:481–90.

42. Dekou V, Gudnason V, Hawe E, Miller GJ, Stansbie D, Humphries SE. Gene-environment and gene-gene interaction in the determination of plasma homocysteine levels in healthy middle-aged men. Thromb Haemost. 2001;85:67–74.

43. Williams SR, Yang Q, Chen F, Liu X, Keene KL, Jacques P, Chen W-M, Weinstein G, Hsu F-C, Beiser A, et al. Genome-Wide Meta-Analysis of Homocysteine and Methionine Metabolism Identifies Five One Carbon Metabolism Loci and a Novel Association of ALDH1L1 with Ischemic Stroke. PLoS Genet. 2014;10:e1004214.

44. George EI. The Variable Selection Problem. J Am Stat Assoc. 2000;95:1304–8.

45. Greenland S. Modeling and variable selection in epidemiologic analysis. Am J Public Health. 1989;79:340–9.

46. Walter S, Tiemeier H. Variable selection: current practice in epidemiological studies. Eur J Epidemiol. 2009;24:733–6.

47. Morozova O, Levina O, Uusküla A, Heimer R. Comparison of subset selection methods in linear regression in the context of health-related quality of life and substance abuse in Russia. BMC Med Res Methodol. 2015;15:71.

48. Hoffer LJ. Homocysteine remethylation and trans-sulfuration. Metabolism. 2004;53:1480–3.
