# Supplementary material for: A novel nutritional supplement to reduce plasma homocysteine in nonpregnant women: A randomised controlled trial in The Gambia
Source: PLoS Med. 2019 Aug 13;16(8):e1002870. doi: 10.1371/journal.pmed.1002870 (PMC6691988; doi:10.1371/journal.pmed.1002870)
Supplement: S3 Appendix — (PDF) [file pmed.1002870.s003.pdf]

S3 Appendix: Safety and dosage considerations for the drink powder supplement

1.1 Introduction

Information presented in supplementary file S2 provided the analyses leading to the design of the novel nutritional supplement, called the 'MDEG-2 Methyl Donor Supplement'. We analysed three separate datasets from West Kiang to determine the specific consistent nutritional predictors of decreased plasma homocysteine. These were folate, B2, B12 and betaine, and provided the final list of components for supplement consideration. Whilst the ingredients comprising this supplement have been widely used before (see review below), this is the first time to our knowledge this specific combination and dosage of micronutrients have been used.

In the following sections we provide a summary of the overall product characteristics of the MDEG-2 methyl donor supplement. We then specifically focus on characteristics of betaine, since this is the component that has been less commonly used in nutritional supplements in The Gambia. We finish with a brief review of the use of Vitamins B12, B2 and folic acid.

1.2 Overall characteristics of the MDEG-2 Methyl Donor Supplement

The novel supplement is a white, crystalline powdered, water-soluble nutritional supplement provided in daily dose sachets of net weight 4011mg. It is designed to be taken orally after being fully dissolved in 200ml of water. The dosage for adults 16 years and older is one sachet (4011mg) per day for 12 weeks. The supplement has been designed to reduce plasma homocysteine concentrations. Table 1.1 details the product formulation.

Table 1.1: MDEG-2 Methyl Donor Supplement active ingredients

Active Ingredients	Generic Name	Anatomical Therapeutic Chemical code*	Quantity of pure active substance	Total quantity of chemical compound (mg)
Vitamin B2	Riboflavin 5-phosphate	A11HA04	2.80mg	4.40
Vitamin B9	Folic Acid	B03BB01	800µg	0.90
Vitamin B12	Cyanocobalamin	B03BA01	5.2 µg	5.70
Betaine	Betaine	A16AA06	4g	4000.00
**TOTAL WEIGHT**				**4011.0 mg**

* The Anatomical Therapeutic Chemical classification system is designated by the WHO Collaborating Centre for Drug Statistics Methodology: https://www.whocc.no/atc/structure_and_principles/

Potential drug interactions

Folic acid can decrease the effectiveness of the drugs used to prevent seizures: Fosphenytoin (Cerebyx), Phenobarbital (Luminal), Phenytoin (Dilantin), Primidone (Mysoline). It can also decrease the effectiveness of certain drugs for cancer treatment: Adrucil (Fluorouracil), Xeloda (capecitabine), Methotrexate (MTX, Rheumatrex). Patients at risk of seizure or undergoing cancer treatment should not receive this supplement. Folic acid, in high doses, can reduce the effectiveness of antifolate antimalarials (such as Fansidar) [1]. The risk of this is low in the study population given folate status is low to start with and the supplement contains a low dose of folic acid, however, women taking antifolate antimalarials should be closely monitored for any signs of malaria.

Fertility

There is not yet epidemiological data on use of this supplement in pregnancy and lactation given it is the first time of usage. However, folic acid, B12 and B2 are commonly administered in pregnancy at the doses contained in this supplement. There have been no documented adverse effects of betaine given in pregnancy, although data is limited. Animal reproduction studies have not been conducted. Administering this supplement during pregnancy is compatible with good maternal and fetal outcomes based on available literature [2,3]. There is currently lack of data on whether betaine anhydrous is excreted in human milk. Supplementation of lactating women should be done with careful monitoring until more data is available. No data is available on effects of the supplement on fertility.

Undesirable effects

Riboflavin may cause urine to be more yellow in colour. Betaine may increase plasma levels of methionine. There are no reported cases of overdose for any of the supplement ingredients.

Pharmacological properties

The supplement reduces levels of homocysteine by tackling the two pathways of methylating homocysteine to methionine. Chapter 6 describes the mechanisms by which homocysteine can be reduced in detail. In brief, one way in which this can happen is by accepting a methyl group to form methionine [4], using two distinct pathways. The major one is the vitamin B12-dependent reaction involving folate metabolic pathways [5], chiefly the donation of a methyl group from N^5-methyl tetrahydrofolate. An alternative pathway, predominantly used in the liver and kidneys, uses the methyl group from betaine, a product formed through the oxidation of choline [6,7]. Homocysteine can also be removed through its irreversible degradation to cystathionine and cysteine in the transsulfuration pathway requiring vitamin B6 [5].

Storage

The supplement should be stored in a dry place below 25˚C. Expiry of the unopened supplement is two years after the date of manufacture.

1.3 Characteristics of betaine

Table 1.2 provides some of the physical, chemical and pharmaceutical properties of the betaine dose used in the supplement.

Table 1.2: Overview of key properties of betaine

**Daily dose:**	4g
**PubChem CID:**	247
**Chemical formula:**	$C_5H_{11}NO_2$
**Physical properties:**	Solid, molecular weight 117.148 g/mol, sweet taste, water-soluble [8]
**Synonyms:**	Glycine betaine, trimethylglycine, betaine anhydrous, Cystadane®
**Pharmaceutical properties:**	Methylation agent used to lowers homocysteine, to increase low plasma methionine and S-adenosyl methionine (SAM) in patients with MTHFR deficiency, to treat homocystinuria [9].

In a toxicology study of male and female Sprague-Dawley rats (n=50), 0, 1, 2, and 5% betaine (corresponding to 0-7143mg/kg) was added to a maintenance chow and the rats were followed for up to 90 days. No toxicity occurred [10]. It is reported that doses equal to or greater than 10,000 mg/kg in rats frequently caused death [11]. For human use, the manufacturers of Cystadane® (oral betaine anhydrous powder) state that whilst long-term carcinogenicity and reproductive toxicity studies have not been conducted, a ‘standard battery of genotoxicity test reveals no specific hazard for humans’ [9]. Betaine is categorised for hepatotoxicity as likelihood score E by the US National Library of Medicine, which corresponds to an ‘unlikely cause of clinically apparent liver injury’ and that ‘despite extensive use, there is no evidence that the drug has caused liver injury’ [12].

Effects in humans

Betaine is a natural food product, but has been used therapeutically in humans to reduce homocysteine. Table 1.3 summarises betaine supplementation intervention trials in humans.

Table 1.3: Summary of trials assessing effect of oral betaine on homocysteine

Reference	Betaine daily dose	Duration of trial	Participants	Results	Serious adverse events?
Schwab *et al.* (2002) [13]	6g	12 weeks	N=42, mean age = 44 years, healthy adults	Reduction of Hcy by 1µmol/l	No
Steenge *et al.* (2003) [14]	6g	6 weeks	N=24, mean age = 44.5 years, healthy adults	Reduction of Hcy by 1.8µmol/l	No
Olthof *et al.* (2003) [15]	6g	6 weeks	N=38, healthy adults	Reduction of Hcy by 2.2µmol/l	No
Olthof *et al.* (2006) [16]	6g	6 weeks	N=39, mean age = 59 years, healthy adults	Reduction of Hcy by 1.2µmol/l	No
Schwab *et al.* (2011) [17]	4g	24 weeks	N=63, mean age = 27 years, healthy adults	No significant reduction of Hcy.	No

In a meta-analysis of the studies included the Table 1.3 the authors concluded that 'supplementation of betaine at 4 to 6 g/d significantly lowers plasma homocysteine concentration in healthy adults by 1.23 µmol/L or 11.8% of baseline values' [18].

There were no serious adverse effects in any of the trials listed in Table 1.3. In larger doses of 6g/day three small studies have reported a small rise of 10mg/dL low-density lipoprotein cholesterol, however, this effect has not been consistently seen in trials and is not considered to be a clinically significant side effect [18].

The Summary of Product Characteristics documentation for Cystadane® contains information on side effects. Cystadane® is used to treat homocystinuria and is recommended in a dose of 100mg/kg/day. For a 60kg adult this would be 6g total per day. Documented side effects for Cystadane® are provided in Table 1.4. Note that the side-effects of brain oedema and blood methionine increases in Table 1.4 are restricted to patients with CBS deficiency. In all cases symptoms were reversed and complete recovery attained after stopping treatment [9]. The UK National Institute for Health and Care Excellence (NICE) report all side-effects related to oral betaine as uncommon [19]. No cases of overdose have been reported and no drug interaction studies have been performed.

Table 1.4: Side-effects for Cystadane®

Side-effect category	Frequency*
Metabolism and nutrition disorders	Uncommon: anorexia
Psychiatric disorders	Uncommon: agitation, irritability
Nervous system disorders	Uncommon: brain oedema
Gastrointestinal disorders	Uncommon: diarrhoea, glossitis, nausea, stomach discomfort, vomiting
Skin and subcutaneous tissue disorders	Uncommon: hair loss, hives, skin odour abnormal
Renal and urinary disorders	Uncommon: urinary incontinence
Investigations	Very common: blood methionine increased

***Frequency**: 'uncommon' (≥ 1/1,000 to < 1/100); very common (≥1/10)
Source: Cystadane® Summary of Product Characteristics [9]

Overall summary of safety of betaine in humans

Betaine anhydrous is Generally Recognised As Safe (GRAS) and so is exempted from the usual Federal Food, Drug, and Cosmetic Act (FFDCA) food additive tolerance requirements. This means there are no official upper limits to its consumption. However, the UK National Institute for Health and Care Excellence advise a maximum of 20g per day [19]. With the proposed dosage of 4g/day there are no foreseen adverse effects related to the consumption of this supplement ingredient.

1.4 Characteristics of Vitamins B2, B12 and Folic Acid

We present some of the physical, chemical and pharmaceutical properties of the B vitamins used in the supplement in Table 1.5. All three vitamins can be found naturally in certain foods. Folate is found in green leafy vegetables, liver and wholegrain cereals. Vitamin B2 is found in milk, eggs, yeast and liver. Vitamin B12 is found in liver, meat and dairy foods.

Table 1.5: Overview of properties of folic acid, B12 and B2

	**Vitamin B2** [20]	**Vitamin B12** [21]	**Folic Acid** [22]
**Daily dose:**	2.80mg	5.2 µg	800µg
**PubChem CID:**	493570	5311498	6037
**Chemical formula:**	$C_{17}H_{20}N_4O_6$	$C_{63}H_{88}CoN_{14}O_{14}P$	$C_{19}H_{19}N_7O_6$
**Molecular Weight:**	376.369 g/mol	1355.388 g/mol	441.404 g/mol
**Physical properties:**	Water-soluble, yellow crystalline powder, solid, bitter	Water-soluble, red crystalline powder, solid, tasteless	Low water solubility, yellow crystalline powder, tasteless
**Synonyms:**	Riboflavin 5-phosphate, lactoflavin, riboflavin, Vitamin G, lactoflavine	Cyanocobalamin, cobalamin, 68-19-9, crystamine, anacobin	59-30-3; folate, pteroylglutamic acid, Vitamin M, Folacin
**Pharmaceutical properties:**	Co-enzyme role in energy production and 1-carbon metabolism	Hematopoiesis, neural metabolism, DNA and RNA production, macronutrient metabolism and 1-carbon metabolism	Hematopoiesis, one-carbon donor, purine & pyrimidine synthesis

Effects in humans

Folic acid, B12 and B2 are extremely common supplements provided in research studies and public health campaigns. In humans there is no reported toxicity for riboflavin, B12 and folic acid given in oral doses. Table 1.6 summarises the previous doses given in different countries in supplementation trials for a variety of health outcomes. It shows how the doses provided in the MDEG-2 methyl donor supplement have been already been commonly utilised. Further information of each of the studies included in this table is provided in **Annex Table 1** below, which details the trial population, location, doses, primary outcomes and whether any side effects were reported.

Table 1.6: Overview of previous doses given of folic acid, B2 and B12

Ingredient	Recommended daily intakes and biomarker levels	Amount in MDEG2 Methyl Donor Supplement	Amounts given in previous trials
Folate given as folic acid	UK RDA: 200 µg/d Pregnancy + 100 µg/d [23]<br><br>USA RDA: 400 µg/d in pregnancy [24]	800 µg/d<br><br>(2xRecommended Daily Allowance [RDA])	200 µg/d [25]<br>215 µg/d [26]<br>300 µg/d [27]<br>350 µg/d [28]<br>400 µg/d [29–43]<br>500 µg/d [44]<br>600 µg/d [45]<br>800 µg/d [14,29,46–48]<br>Increments between 50–800µg/d [49]<br>150-2500 µg/d [50]<br>2500 µg/d [51]<br>4000 µg/d [52]<br>2800 µg/wk [53]
Vitamin B12 given as cyanocobalamin	UK RDA : 1.5µg/d [23]<br><br>USA RDA: 2.6 µg/d in pregnancy [24]	5.2 µg/d<br><br>(2xRDA)	2.0 µg/d [26]<br>2.2 µg/d [34]<br>2.6 µg /d [30–32,37–43,45]<br>3 µg/d [28]<br>4 µg/d [48,54]<br>5.2 µg/d [35]<br>6 µg/d [36]<br>20 µg/d [55]<br>50 µg/d [47]<br>400 µg/d [46,51]
Riboflavin (vitamin B2) given as Riboflavin-5'-phosphate	UK RDA for adult women:<br>1.1mg/d, +0.3mg in pregnancy [23]<br><br>USA RDA: 1.4 mg/d in pregnancy [24]	2.8 mg/d<br><br>(2xRDA)	1.4 mg/d [30–32,38–41,43,45]<br>1.5mg/d [52]<br>1.6 mg/d [25,54]<br>1.8 mg/d [37,48]<br>1.9 mg/d [26,42]<br>2 mg/d [36]<br>2.8 mg/d [35]<br>5 mg/d [27]<br>15 mg/d [28]<br>20 mg/d [47]

There are no reported serious adverse effects to taking riboflavin supplements [56], however, some individuals may find their urine is more yellow in colour than normal. There are no documented significant adverse effects of taking B12 supplement, even at doses several hundred times what we are planning to give [56]. There are no documented significant adverse effects of taking folic acid supplements at this dose.

Overall summary of safety in humans

Supplement doses for folic acid, B2 and B12 are proposed at twice the Recommended Daily Allowance (RDA) set for the United States of America [24] in order to correct the micronutrient deficiencies commonly found in the Gambian dry season.

Folic acid (proposed supplement 800 µg/d, 2xRDA):

The proposed supplement dose has been used before in several large trials [46–48], and is well below the amount expected to cause adverse effects. The Expert Group on Vitamins & Minerals (2003) states that "in the general population a supplemental dose of 1 mg/day (equivalent to 0.017 mg/kg bw/day in a 60 kg adult) would not be expected to cause adverse effects" [57]. The European Agency for Food Safety (2006) states that the Lowest observed-adverse-effect level (LOAEL) is 5 mg per day [56], and current US government advice for women who have previously had a baby with a birth defect is to take up to 4000 µg/d [58]. Side effects have only been documented with doses in excess of 1mg/day.

Riboflavin (B2) (proposed supplement 2.8 mg/d, 2xRDA):

Riboflavin is Generally Recognised As Safe (GRAS), even when given in doses up to 14 times what we plan to give [59]. GRAS Report 114 on Riboflavin states that "there is no evidence in the available information on riboflavin or riboflavin-5'-phosphate that demonstrates or suggest reasonable grounds to suspect, a hazard to the public when they are used at levels that are now current or that might reasonably be expected in the future" [59]. The Expert Group on Vitamins & Minerals (2003) also concur, stating that "supplemental intakes of 40 mg riboflavin/day (equivalent to 0.67 mg/kg bw for a 60 kg adult) would be unlikely to result in adverse effects. This is in addition to riboflavin provided by the diet" [57].

Cyanocobalamin (B12) (proposed supplement 5.2 µg/d, 2xRDA):

The GRAS Report 104 on Cyanocobalamin states that "there is no evidence in the available information on vitamin B12 that demonstrates, or suggests reasonable grounds to suspect, a hazard to the public when it is used at levels that are now current and in the manner now practiced, or that might reasonably be expected in the future" [59]. The Expert Group on Vitamins & Minerals (2003) states that "it is generally accepted that ingested vitamin B12 (cobalamin) has a very low toxicity in humans…supplemental 2.0 mg cyanocobalamin/day should not produce any adverse effects and this intake can be used for guidance purposes" [57].

1.5 Overall conclusion on usage of MDEG-2 Methyl Donor Supplement

The ingredients included in this supplement are extremely unlikely to result in any adverse events when taken at the correct dosage of one sachet per day. There are many existing commercial formulations that contain doses several hundred to several thousand times more than the MDEG-2 methyl donor supplement (e.g. Bluebonnet Nutrition Homocysteine Formula, Thorne Methyl Guard Plus, Biocare Methyl Multinutrient). Considering the research

project will be delivered under observed supplementation, and provided against the backdrop of micronutrient deficiencies associated with the Gambian dry season, it is considered a safe nutritional supplement tailored to the requirements of the target population.

Annex Table 1: Selection of B vitamin and folic acid trials detailing dosage and side effect information

Study	Design	Population	Micronutrients Provided	Outcome investigated	Results	Side effects
Bhutta Z, et al. (2009) [30]	RCT, daily supplementation in pregnancy. Fe-Fol vs. MMN	2,378 pregnant women, Pakistan	Fe, **folic acid (400μg),** vitamins A, D, E, C, B1, niacin, **B2 (1.4mg),** B6, **B12 (2.6μg),** Zn, Cu, Se, I (UNIMMAP)	Birthweight	Increase of 70g birthweight in MMN arm.	Comparable rates of self-reported morbidities between arms. Minimal reported gastrointestinal side effects.
Brough L, et al. (2010) [36]	RCT, daily supplementation in pregnancy. MMN vs. placebo	402 pregnant women, UK	β-carotene, B1, **B2 (2mg),** B6, **B12 (6μg), folic acid (400μg)**, C, D, E, K, Fe, Zn, Mg, I, Cu	Maternal nutrient status, birth size	↑ markers of Fe, folate, B1 and vitamin D in MMN arm in third trimester. In compliant subset MMN arm had ↓SGA infants (borderline significance)	No difference in reported side effects.
Christian P, et al. (2003) [37]	Cluster RCT, daily supplementation in pregnancy. Vit A (control) vs. Vit A + FA vs. Vit A + FA + Fe vs. Vit A + FA + Fe + Zn vs. Vit A + Fe + MMN	4926 pregnant women, Nepal	Vitamin A, **folic acid (400μg)**, Fe, Zn, D, E, B1, **B2 (1.8mg),** B6, **B12 (2.6μg),** C, K, niacin, Cu, and Mg	Foetal loss and infant mortality	No effect of any arm on reduction of overall foetal loss. In stratified analyses the Vit A+FA arm and Vit A + FA + Fe arms ↓ 3-month mortality amongst pre-terms compared to control. The MMN arm showed ↑ risk of mortality in term infants (borderline significance).	No maternal side effects reported here.
Dawson S et al. (2016) [50]	Meta-analysis of 21 studies supplementing with either Omega-3 or Omega-3 + FA + B6 + B12 in daily supplementation	Range of populations	**Folic acid (150 – 2500μg),** omega-3, B6, **B12 (1.9 μg – 12mg)**	Plasma homocysteine	Trials combining omega-3 with FA and B vitamins reduced plasma Hcy the most.	Not reported.
Ebbing M, et al. (2009) [46]	RCT, daily supplementation for median 39 months.	6,837 participants with ischaemic	**Folic acid (800μg), B12 (400μg),** B6	Cancer incidence, mortality	Increased lung cancer risk and cancer mortality in FA+B12 arm vs. control. No increased risks in B6 arms.	No minor side effects reported but see results section for

Study	Design	Population	Micronutrients Provided	Outcome investigated	Results	Side effects
	FA+B12+B6 vs. FA+B12 vs. B6 vs. placebo	heart disease, Norway				possible serious adverse events.
Fawzi W, et al. (2007) [47]	RCT, daily supplementation in pregnancy to 6 weeks post-partum. MMN + Fe-Fol vs. placebo + Fe-Fol	8,468 HIV-negative pregnant women, Tanzania	B1, **B2 (20mg),** B6, niacin, **B12 (50 µg),** C, E**, folic acid (800µg)**	Low birth weight, prematurity, foetal death	15% lower risk of LBW and 67g higher birthweight in MMN arm. No effect on preterm birth or foetal death.	No difference in maternal mortality or miscarriages by arm.
Friis H, et al. (2004) [54]	RCT, daily supplementation in pregnancy until deliver. Fe-Fol + MMN vs. Fe-Fol	1,106 pregnant women, Zimbabwe.	Folic acid, Fe, vitamins A, D, E, C, B1, niacin, **B2 (1.6mg),** B6, **B12 (4.0µg),** Zn, Cu, Se	Birth outcomes	46g increase in the MMN + Fe-Fol arm but borderline significance.	Maternal side effects not reported.
Galan P et al. (2010) [55]	RCT, daily supplementation for median 4.7 years. Omega 3 vs. omega 3 + B vits vs. B vits vs. placebo	2,501 patients with a history of myocardial infarction, unstable angina, or ischaemic stroke, France	5-methyltetrahydrofolate, B6, **B12 (20 µg)**, omega-3 fatty acids	Prevention of cardiovascular events	B vitamins arm ↓ plasma homocysteine concentrations by 19% compared with placebo, but had no effect on major vascular events. No effect of omega-3 on vascular events compared to placebo.	2.1% of people experienced side effects (mainly gastrointestinal and nausea) in the B vitamins group, compared with 2.6% in the omega-3 fatty acids group, and 1.6% in the placebo group.
Hall M et al. (2016) [29]	RCT, daily supplementation for 12 weeks. FA (800µg) vs. FA (400µg) vs. creatine vs. creatine + FA 400µg vs. placebo	622 arsenic-exposed adults, Bangladesh	**Folic acid (800µg, 400µg),** creatine	Plasma choline, betaine and dimethylglycine (DMG) concentrations	FA groups ↑plasma betaine, ↓DMG and prevented a ↓ in choline. No difference when adding creatine.	Not reported.

Study	Design	Population	Micronutrients Provided	Outcome investigated	Results	Side effects
Hininger I, et al. (2004) [25]	RCT, daily supplementation for two months, MMN vs. placebo	100 healthy pregnant women, France	**Folic acid (200µg),** vitamin C, E, B1, **B2 (1.6mg),** B6, **B12 (1µg),** pantothenic acid, β-carotene, Zn, Mg, Ca	Plasma micronutrient levels, oxidative stress parameters	↑Folic acid, vitamin C, E, B2, B6 and β-carotene concentration in MMN arm. No difference in oxidative stress parameters.	Not reported.
Liu J et al. (2013) [39]	RCT, daily supplementation in pregnancy, FA (control) vs. FA + Fe vs. FA + Fe + MMNs	18,775 pregnant women, China	Fe, **folic acid (400µg),** vitamins A, D, E, C, B1, niacin, **B2 (1.4mg),** B6, **B12 (2.6µg),** Zn, Cu, Se, I (UNIMMAP)	Perinatal mortality, neonatal mortality, birth size, maternal nutrient status	No effect of supplements on perinatal mortality. Supplement arms ↓ maternal anaemia in third trimester (~28% risk reduction).	No serious adverse effects reported. 6% reported gastrointestinal discomfort in MMN group vs. only 2.2% in FA controls.
Kaestel P, et al. (2005) [38]	RCT, daily supplementation in pregnancy, Fe-Fol (control), vs. 15 micronutrients at 1xRDA (MMN1) vs. 15 micronutrients at 2xRDA (MMN2)	2,100 pregnant women, Guinea Bissau	Fe, **folic acid (400µg, 800µg),** vitamins A, D, E, C, B1, niacin, **B2 (1.4mg, 2.8mg),** B6, **B12 (2.6µg, 5.2µg),** Zn, Cu, Se, I (UNIMMAP)	Birthweight and birth outcomes	MMN1 and MMN2 ↑ birthweight compared to control, 53g and 95g respectively. No difference in proportion of LBW. Larger effect with MMN2 in anaemic women (↑ birthweight by 218g and ↓LBW risk by 31%).	Lower risk of miscarriage in MMN2 group (but not MMN1 group) compared to control. No difference in maternal mortality between arms.
Kirke P, et al. (1992) [27]	RCT, daily supplementation pre-conception for at least two months. FA vs. MMN vs. FA+MMN	354 women, Ireland	FA: **Folic acid (0.36mg),** MMN: Vitamins A, D, B1, **B2 (1.5mg)**, B6, niacin, C, Ca, Fe	Neural tube defects	No statistical difference in prevention of NTDs per arm.	No statistically significant differences in pregnancy outcome information by trial arm.
Kumwenda N, et al. (2002) [33]	RCT, daily supplementation in pregnancy. Fe-Fol vs. Fe-Fol + Vit A	697 HIV-positive pregnant women, Malawi	**Folic acid (400µg),** Fe, Vit A	Birthweight	Fe-Fol plus Vit A arm ↑birthweight (90g).	Not reported.

Study	Design	Population	Micronutrients Provided	Outcome investigated	Results	Side effects
Melse-Boonstra A, et al. (2005) [49]	RCT, daily supplementation. 6 incremental doses of FA vs. placebo	308 adults (50-75y), The Netherlands	**Folic acid (50-800μg),**	Plasma betaine and homocysteine (Hcy)	Plasma concentration of Hcy ↓ as ↑folate and ↑ betaine. Plasma betaine ↑ as FA dose ↑.	Not reported.
Moore SE, et al. (2012) [35]	RCT, daily supplementation in pregnancy. Protein-energy (PE) + MMN, PE, MMN, Fe-Fol	620 pregnant women, The Gambia	Fe, **folic acid (400μg),** vitamins A, D, E, C, B1, niacin, **B2 (2.8mg),** B6, **B12 (5.2μg),** Zn, Cu, Se, I, protein, lipids	Thymic index	Not yet reported for primary outcome. Secondary outcome analysis suggests no influence of supplementation on foetal growth [60].	Not yet reported
Wald N et al. (1991) [52]	RCT, daily supplementation pre-conception until week 12 of pregnancy. FA vs. FA + MMN vs. MMN vs. placebo	1,817 women in 7 countries	**Folic acid (4mg),** MMN: Vit A, D, B_1, **B2 (1.5mg)**, B6, C, nicotinamide	Neural tube defects	FA groups had ↓ risk of NTDs. No protection from MMNs without FA.	No difference in self-reported side effects between arms.
Osrin D, et al. (2005) [40]	RCT, daily supplementation in pregnancy. Fe-Fol (control) vs. MMN	1,200 pregnant women, Nepal	Fe, **folic acid (400μg),** vitamins A, D, E, C, B1, niacin, **B2 (1.4mg),** B6, **B12 (2.6μg),** Zn, Cu, Se, I (UNIMMAP)	Birthweight, gestational length	MMN arm ↑ birthweight by 77g and ↓ LBW by 25%.	Fewer miscarriages in the MMN arm (5 vs. 2). No differences in gastrointestinal complaints between the arms.
Ramakrishnan U, et al. (2003) [26]	Double-blind RCT, 6 days/week for at least two months, MMN vs. Fe-only	873 pregnant women, Mexico	Vitamin A, B1, D, E, **B2 (1.87mg),** niacin, **folic acid (215μg),** B6, **B12 (2.04μg),** C, Zn, Fe, Mg	Birth size	No effect of multiple micronutrients versus iron alone	None reported.
Ramakrishnan U, et al. (2016) [53]	RCT, daily supplementation pre-conception to conception (mean 33 weeks). FA vs.	1,813 women, Vietnam	**Folic acid (2.8mg),** Fe, A, D, E, C, B1, niacin, **B2 (1.4mg),** B6, **B12 (2.6μg),** Zn, Cu, Se, I	Birth outcomes	No difference in birthweight, gestational age, preterm delivery between arms.	No difference in reported side effects by arm.

Study	Design	Population	Micronutrients Provided	Outcome investigated	Results	Side effects
	FA + Fe vs. FA + FE + MMNs					
Roberfroid D, et al. (2008) [41]	RCT, daily supplementation in pregnancy. Fe-Fol (control) vs. MMN	1,426 pregnant women, Burkina Faso	Fe, **folic acid (400µg),** vitamins A, D, E, C, B1, niacin, **B2 (1.4mg),** B6, **B12 (2.6µg),** Zn, Cu, Se, I (UNIMMAP)	Fetal growth	MMN arm ↑ birthweight by 52g, no difference in risk of LBW	No difference in miscarriage between arms. No other maternal side effects reported.
Rumiris D, et al. (2006) [34]	RCT, daily supplementation in pregnancy. MMN vs. Fe-Fol (control)	Pregnant women with low antioxidant status	Vitamins A, B6, **B12 (2.2 µg)**, C, E, **folic acid (400 µg**), N-acetylcysteine, Cu, Zn, Mn, Fe, Ca, Se	Perinatal outcomes	↓Preeclampsia in the MMN group compared to control	Not reported.
Rush et al. (1980) [28]	RCT, daily supplementation in pregnancy, protein-energy (PE) + MMN vs. MMN vs. standard care.	1,051 pregnant women, USA	Ca, Mg, Fe, Zn, Cu, I, Vitamins A, D, E, C, B1, **B2 (15mg),** niacin, B6, pantothenic acid, biotin, **folic acid (350µg), B12 (8µg),** protein, carbohydrate, fat	Birthweight, post-natal development	PE+MMN prevented low birthweight in mothers who smoked heavily.	Higher neonatal deaths in supplement group although not statistically significant (and note this was in the arm containing additional protein-energy).
Shankar AH et al. (2008) [42]	RCT, daily supplementation in pregnancy, Fe-Fol vs. MMN	31,290 pregnant women, Indonesia	Fe, **folic acid (400µg),** vitamins A, D, E, C, B1, niacin, **B2 (1.4mg),** B6, **B12 (2.6µg),** Zn, Cu, Se, I (UNIMMAP)	Infant mortality, foetal loss, birthweight	↓Early infant mortality (18% reduction) in the MMN group compared to control. Combined foetal loss and neonatal mortality ↓ by 11% in the MMN arm. LBW ↓ by 14% in MMN arm.	Not reported.
Tofail F, et al. (2008) [43]	RCT, daily supplementation week 14 gestation to delivery. MMN (and early food) vs. Fe (30mg)-fol (and early food) vs. Fe (60mg)-fol	2,853 pregnant mothers, Bangladesh	Fe, **folic acid (400µg),** vitamins A, D, E, C, B1, niacin, **B2 (1.4mg),** B6, **B12 (2.6µg),** Zn, Cu, Se, I (UNIMMAP)	Infant development	No intervention effects on whole group, but varying effects of the 6 different groups for low BMI mothers.	Not reported.

**Study**	**Design**	**Population**	**Micronutrients Provided**	**Outcome investigated**	**Results**	**Side effects**
	(and early food) vs. MMN (normal food) vs. Fe (30mg)-fol (normal food) vs. Fe (60mg)-fol (normal food)					
Toole J et al. (2004) [51]	RCT, daily supplementation for mean 20 months, high dose vs. low dose B6, B12, FA.	3,680 adults with non-disabling cerebral infarction, USA, Canada Scotland	High dose: B6, **B12 (0.4mg), folic acid (2.5mg)**<br><br>Low dose: B6, **B12 (6μg), folic acid (20 μg)**	Plasma Homocysteine, stroke	Plasma Hcy ↓ by 2μmol/L in high dose group compared to low dose group. No effect on stroke or death.	No significant differences between groups for itching, skin rash, or gastrointestinal complaints.
West K et al. (2014) [45]	RCT, daily supplementation in pregnancy to 12 weeks postpartum, Fe-Fol vs. MMN.	44,567 pregnant women, Bangladesh	Fe, **folic acid (400μg),** vitamins A, D, E, C, B1, niacin, **B2 (1.4mg),** B6, **B12 (2.6μg),** Zn, Cu, Se, I (UNIMMAP)	Birth outcomes, 6-month infant mortality	No effect on 6-month infant mortality. MMN arm ↓ pre-term birth by 15% and ↓LBW by 12%.	No differences in morbidities between arms.
Zagré N et al. (2007) [31]	RCT, daily supplementation in pregnancy, Fe-Fol vs. MMN.	3,670 pregnant women, Niger	Fe, **folic acid (400μg),** vitamins A, D, E, C, B1, niacin, **B2 (1.4mg),** B6, **B12 (2.6μg),** Zn, Cu, Se, I (UNIMMAP)	Birthweight	Birthweight 67g higher in MMN arm compared to Fe-Fol arm.	Miscarriages and maternal deaths were comparable in both groups
Zeng, L et al. (2008) [32]	Cluster RCT, daily supplementation in pregnancy. FA (control) vs. Fe-Fol vs. MMN	5,828 pregnant women in China	Fe, folic acid **(400 μg)**, Zn, Cu, Se, I, Vitamins A, B1, **B2 (1.4 mg), B12 (2.6μg)**, D, C, E, niacin.	Birth size	Birthweight 42g higher in MMN group compared to FA group. Fe-Fol reduced risk of pre-term delivery.	No differences in perinatal mortality between groups.

Abbreviations: BMI, body mass index; FA, folic acid; Fe-Fol, iron-folic acid supplement; Hcy, homocysteine; LBW, low birthweight; MMN, multiple micronutrient supplement; NTD, neural tube defect; RCT, randomised controlled trial; RDA, recommended daily allowance; UNIMMAP, United Nations International Multiple Micronutrient Preparation

References

1. Verhoef H, Veenemans J, Mwangi MN, Prentice AM. Safety and benefits of interventions to increase folate status in malaria-endemic areas. Br J Haematol. 2017;177: 905–918.
2. Ueland PM. Choline and betaine in health and disease. J Inherit Metab Dis. 2011;34: 3–15.
3. Shaw GM, Carmichael SL, Yang W, Selvin S, Schaffer DM. Periconceptional dietary intake of choline and betaine and neural tube defects in offspring. Am J Epidemiol. 2004;160: 102–9.
4. Robinson JL, Bertolo RF. The Pediatric Methionine Requirement Should Incorporate Remethylation Potential and Transmethylation Demands. Adv Nutr. 2016;7: 523–34.
5. Selhub J. Homocysteine metabolism. Annu Rev Nutr.1999;19: 217–46. doi:10.1146/annurev.nutr.19.1.217
6. Velzing-Aarts F V, Holm PI, Fokkema MR, van der Dijs FP, Ueland PM, Muskiet FA. Plasma choline and betaine and their relation to plasma homocysteine in normal pregnancy. Am J Clin Nutr. 2005;81: 1383–9.
7. McKeever MP, Weir DG, Molloy A, Scott JM. Betaine-homocysteine methyltransferase: organ distribution in man, pig and rat and subcellular distribution in the rat. Clin Sci. 1991;81: 551–6.
8. National Center for Biotechnology Information (2017) Compound Summary for CID 247 (Betaine). PubChem Compd. Database. https://pubchem.ncbi.nlm.nih.gov/compound/betaine.
9. Orphan Europe S.A.R.L. Cystadane Summary of Product Characteristics. Puteaux, France; 2007.
10. Hayes KC, Pronczuk A, Cook MW, Robbins MC. Betaine in sub-acute and sub-chronic rat studies. Food Chem Toxicol. 2003;41: 1685–700.
11. DrugBank. Glycine Betaine. Available: http://www.drugbank.ca/drugs/DB06756
12. Liver Disease Research Branch of the National Institute of Diabetes and Digestive and Kidney Diseases (NIDDK) and the Division of Specialized Information Services of the National Library of Medicine (NLM). LiverTox: Betaine. Available: https://livertox.nlm.nih.gov//Betaine.htm
13. Schwab U, Törrönen A, Toppinen L, Alfthan G, Saarinen M, Aro A, et al. Betaine supplementation decreases plasma homocysteine concentrations but does not affect body weight, body composition, or resting energy expenditure in human subjects. Am J Clin Nutr. 2002;76: 961–7.
14. Steenge GR, Verhoef P, Katan MB. Betaine supplementation lowers plasma

homocysteine in healthy men and women. J Nutr. 2003;133: 1291–5.
15. Olthof MR, van Vliet T, Boelsma E, Verhoef P. Low dose betaine supplementation leads to immediate and long term lowering of plasma homocysteine in healthy men and women. J Nutr. 2003;133: 4135–8.
16. Olthof MR, Bots ML, Katan MB, Verhoef P. Effect of folic acid and betaine supplementation on flow-mediated dilation: a randomized, controlled study in healthy volunteers. PLoS Clin Trials. 2006;1: e10.
17. Schwab U, Alfthan G, Aro A, Uusitupa M. Long-term effect of betaine on risk factors associated with the metabolic syndrome in healthy subjects. Eur J Clin Nutr. 2011;65: 70–76.
18. McRae MP. Betaine supplementation decreases plasma homocysteine in healthy adult participants: a meta-analysis. J Chiropr Med. 2013;12: 20–25.
19. National Institute for Health and Care Excellence. NICE Guidance: Betaine. Available: https://bnf.nice.org.uk/drug/betaine.html
20. National Center for Biotechnology Information. Compound Summary for CID 493570 (Riboflavin). In: PubChem Compound Database.
21. National Center for Biotechnology Information. Compound Summary for CID 5311498 (Vitamin B12). In: PubChem Compound Database.
22. National Center for Biotechnology Information. Compound Summary for CID 6037 (Folic Acid). In: PubChem Compound Database.
23. British Nutrition Foundation. UK Dietary Reference Values. 2015. Available: https://www.nutrition.org.uk/attachments/article/261/Nutrition Requirements_Revised Oct 2016.pdf
24. Institute of Medicine (US) Committee to Review Dietary Reference Intakes for Vitamin D and Calcium. Ross A, Taylor C, Yaktine A, editors. Dietary Reference Intakes for Calcium and Vitamin D. Washington (DC): National Academies Press; 2011.
25. Hininger I, Favier M, Arnaud J, Faure H, Thoulon JM, Hariveau E, et al. Effects of a combined micronutrient supplementation on maternal biological status and newborn anthropometrics measurements: a randomized double-blind, placebo-controlled trial in apparently healthy pregnant women. Eur J Clin Nutr. 2004;58: 52–9.
26. Ramakrishnan U, González-Cossío T, Neufeld LM, Rivera J, Martorell R. Multiple micronutrient supplementation during pregnancy does not lead to greater infant birth size than does iron-only supplementation: a randomized controlled trial in a semirural community in Mexico. Am J Clin Nutr. 2003;77: 720–5.
27. Kirke PN, Daly LE, Elwood JH. A randomised trial of low dose folic acid to prevent neural tube defects. Arch Dis Child. 1992;67: 1442–6.
28. Rush D, Stein Z, Susser M. A randomized controlled trial of prenatal nutritional

supplementation in New York City. Pediatrics. 1980;65: 683–97.
29. Hall MN, Howe CG, Liu X, Caudill MA, Malysheva O, Ilievski V, et al. Supplementation with Folic Acid, but Not Creatine, Increases Plasma Betaine, Decreases Plasma Dimethylglycine, and Prevents a Decrease in Plasma Choline in Arsenic-Exposed Bangladeshi Adults. J Nutr. 2016;146: 1062–7.
30. Bhutta ZA, Rizvi A, Raza F, Hotwani S, Zaidi S, Moazzam Hossain S, et al. A comparative evaluation of multiple micronutrient and iron-folic acid supplementation during pregnancy in Pakistan: impact on pregnancy outcomes. Food Nutr Bull. 2009;30: S496-505.
31. Zagré NM, Desplats G, Adou P, Mamadoultaibou A, Aguayo VM. Prenatal multiple micronutrient supplementation has greater impact on birthweight than supplementation with iron and folic acid: a cluster-randomized, double-blind, controlled programmatic study in rural Niger. Food Nutr Bull. 2007;28: 317–27.
32. Zeng L, Dibley MJ, Cheng Y, Dang S, Chang S, Kong L, et al. Impact of micronutrient supplementation during pregnancy on birth weight, duration of gestation, and perinatal mortality in rural western China: double blind cluster randomised controlled trial. BMJ. 2008;337: a2001.
33. Kumwenda N, Miotti PG, Taha TE, Broadhead R, Biggar RJ, Jackson JB, et al. Antenatal vitamin A supplementation increases birth weight and decreases anemia among infants born to human immunodeficiency virus-infected women in Malawi. Clin Infect Dis. 2002;35: 618–24.
34. Rumiris D, Purwosunu Y, Wibowo N, Farina A, Sekizawa A. Lower rate of preeclampsia after antioxidant supplementation in pregnant women with low antioxidant status. Hypertens pregnancy. 2006;25: 241–53.
35. Moore SE, Fulford AJ, Darboe MK, Jobarteh ML, Jarjou LM, Prentice AM. A randomized trial to investigate the effects of pre-natal and infant nutritional supplementation on infant immune development in rural Gambia: the ENID trial: Early Nutrition and Immune Development. BMC Pregnancy Childbirth. 2012;12: 107.
36. Brough L, Rees GA, Crawford MA, Morton RH, Dorman EK. Effect of multiple-micronutrient supplementation on maternal nutrient status, infant birth weight and gestational age at birth in a low-income, multi-ethnic population. Br J Nutr. 2010;104: 437–45.
37. Christian P, West KP, Khatry SK, Leclerq SC, Pradhan EK, Katz J, et al. Effects of maternal micronutrient supplementation on fetal loss and infant mortality: a cluster-randomized trial in Nepal. Am J Clin Nutr. 2003;78: 1194–202.
38. Kaestel P, Michaelsen KF, Aaby P, Friis H. Effects of prenatal multimicronutrient supplements on birth weight and perinatal mortality: a randomised, controlled trial in

Guinea-Bissau. Eur J Clin Nutr. 2005;59: 1081–9.
39. Liu J, Mei Z, Ye R, Serdula MK, Ren A, Cogswell ME. Micronutrient Supplementation and Pregnancy Outcomes. JAMA Intern Med. 2013;173: 276.
40. Osrin D, Vaidya A, Shrestha Y, Baniya RB, Manandhar DS, Adhikari RK, et al. Effects of antenatal multiple micronutrient supplementation on birthweight and gestational duration in Nepal: double-blind, randomised controlled trial. Lancet. 2005;365: 955–62.
41. Roberfroid D, Huybregts L, Lanou H, Henry M-C, Meda N, Menten J, et al. Effects of maternal multiple micronutrient supplementation on fetal growth: a double-blind randomized controlled trial in rural Burkina Faso. Am J Clin Nutr. 2008;88: 1330–40.
42. Supplementation with Multiple Micronutrients Intervention Trial (SUMMIT) Study Group, Shankar AH, Jahari AB, Sebayang SK, Aditiawarman, Apriatni M, et al. Effect of maternal multiple micronutrient supplementation on fetal loss and infant death in Indonesia: a double-blind cluster-randomised trial. Lancet. 2008;371: 215–27.
43. Tofail F, Persson LA, El Arifeen S, Hamadani JD, Mehrin F, Ridout D, et al. Effects of prenatal food and micronutrient supplementation on infant development: a randomized trial from the Maternal and Infant Nutrition Interventions, Matlab (MINIMat) study. Am J Clin Nutr. 2008;87: 704–11.
44. Schmidt MK, Muslimatun S, West CE, Schultink W, Hautvast JG. Vitamin A and iron supplementation of Indonesian pregnant women benefits vitamin A status of their infants. Br J Nutr. 2001;86: 607–15.
45. West KP, Shamim AA, Mehra S, Labrique AB, Ali H, Shaikh S, et al. Effect of maternal multiple micronutrient vs iron-folic acid supplementation on infant mortality and adverse birth outcomes in rural Bangladesh: the JiVitA-3 randomized trial. JAMA. 2014;312: 2649–58.
46. Ebbing M, Bønaa KH, Nygård O, Arnesen E, Ueland PM, Nordrehaug JE, et al. Cancer incidence and mortality after treatment with folic acid and vitamin B12. JAMA. 2009;302: 2119–26.
47. Fawzi WW, Msamanga GI, Urassa W, Hertzmark E, Petraro P, Willett WC, et al. Vitamins and perinatal outcomes among HIV-negative women in Tanzania. N Engl J Med. 2007;356: 1423–31.
48. Czeizel AE, Dudás I, Métneki J. Pregnancy outcomes in a randomised controlled trial of periconceptional multivitamin supplementation. Final report. Arch Gynecol Obstet. 1994;255: 131–9.
49. Melse-Boonstra A, Holm PI, Ueland PM, Olthof M, Clarke R, Verhoef P. Betaine concentration as a determinant of fasting total homocysteine concentrations and the effect of folic acid supplementation on betaine concentrations. Am J Clin Nutr.

2005;81: 1378–82.

50. Dawson SL, Bowe SJ, Crowe TC. A combination of omega-3 fatty acids, folic acid and B-group vitamins is superior at lowering homocysteine than omega-3 alone: A meta-analysis. Nutr Res. 2016;36: 499–508.
51. Toole JF, Malinow MR, Chambless LE, Spence JD, Pettigrew LC, Howard VJ, et al. Lowering Homocysteine in Patients With Ischemic Stroke to Prevent Recurrent Stroke, Myocardial Infarction, and Death. JAMA. 2004;291: 565.
52. Group. MVSR. Prevention of neural tube defects: results of the Medical Research Council Vitamin Study. MRC Vitamin Study Research Group. Lancet. 1991;338: 131–7.
53. Ramakrishnan U, Nguyen PH, Gonzalez-Casanova I, Pham H, Hao W, Nguyen H, et al. Neither Preconceptional Weekly Multiple Micronutrient nor Iron-Folic Acid Supplements Affect Birth Size and Gestational Age Compared with a Folic Acid Supplement Alone in Rural Vietnamese Women: A Randomized Controlled Trial. J Nutr. 2016; jn223420.
54. Friis H, Gomo E, Nyazema N, Ndhlovu P, Krarup H, Kaestel P, et al. Effect of multimicronutrient supplementation on gestational length and birth size: a randomized, placebo-controlled, double-blind effectiveness trial in Zimbabwe. Am J Clin Nutr. 2004;80: 178–84.
55. Galan P, Kesse-Guyot E, Czernichow S, Briancon S, Blacher J, Hercberg S, et al. Effects of B vitamins and omega 3 fatty acids on cardiovascular diseases: a randomised placebo controlled trial. BMJ. 2010;341: c6273.
56. European Food Safety Authority (2006) Tolerable upper intake levels for vitamins and minerals. EFSA: Parma, Italy.
57. Expert Group on Vitamins and Minerals (EVM) (2003) Safe Upper Levels for Vitamins and Minerals. UK: Food Standards Agency.
58. Office on Women's Health (2012) Folic Acid Fact Sheet. US Department of Healht and Human Services. https://www.womenshealth.gov/publications/our-publications/fact-sheet/folic-acid.html
59. U.S. Food and Drug Administration (1972) GRAS Substances (SCOGS) Database. Select Committee on GRAS Substances, Center for Food Safety and Applied Nutrition.
60. Johnson W, Darboe MK, Sosseh F, Nshe P, Prentice AM, Moore SE. Association of prenatal lipid-based nutritional supplementation with fetal growth in rural Gambia. Matern Child Nutr. 2016; 13: e 12367
