# Supplementary material for: A novel nutritional supplement to reduce plasma homocysteine in nonpregnant women: A randomised controlled trial in The Gambia
Source: PLoS Med. 2019 Aug 13;16(8):e1002870. doi: 10.1371/journal.pmed.1002870 (PMC6691988; doi:10.1371/journal.pmed.1002870)
Supplement: S1 Table — (PDF) [file pmed.1002870.s004.pdf]

S1 Table: Multivariable regression results showing percentage difference in geometric mean plasma homocysteine between trial arms, restricted to fasted, non-pregnant, compliant participants

**Trial arms**	**Timepoint**	**N***	**β (95% CI)****	**% difference in geometric means ƚ**	**P value**
Drink powder vs. control	12 weeks	141	-0.28 (-0.36, -0.19)	-24.1 (-30.1, -17.4)	<0.001
UNIMMAP vs. control	12 weeks	143	-0.18 (-0.25, -0.1)	-16.4 (-22.5, -9.9)	<0.001
Drink powder vs. UNIMMAP	12 weeks	130	-0.10 (-0.18, -0.01)	-9.2 (-16.7, -1.1)	0.028
Drink powder vs. control	5 weeks	176	-0.44 (-0.52, -0.37)	-35.8 (-40.4, -30.8)	<0.001
UNIMMAP vs. control	5 weeks	168	-0.26 (-0.33, -0.18)	-22.5 (-27.9, -16.8)	<0.001
Drink powder vs. UNIMMAP	5 weeks	162	-0.17 (-0.25, -0.1)	-15.9 (-21.9, -9.4)	<0.001

* **β** represents difference in log homocysteine at time point between trial arms, adjusted for baseline homocysteine, age and body mass index at time point.

**Fasted and non-pregnant at time point, and above 80% compliance at time point

ƚ Calculated from $(e^{\beta}-1)*100$
