# Supplementary material for: A novel nutritional supplement to reduce plasma homocysteine in nonpregnant women: A randomised controlled trial in The Gambia
Source: PLoS Med. 2019 Aug 13;16(8):e1002870. doi: 10.1371/journal.pmed.1002870 (PMC6691988; doi:10.1371/journal.pmed.1002870)
Supplement: S2 Table — (PDF) [file pmed.1002870.s005.pdf]

S2 Table: Unadjusted blood pressure and pulse results by timepoint and trial arm

	Baseline		Midline		Endline	
	N	Geometric mean (95% CI)	N	Geometric mean (95% CI)	N	Geometric mean (95% CI)
**Systolic Blood Pressure** (mmHg)						
Control	100	114.4 (111.6, 117.2)	93	107.1 (104.7, 109.5)	82	106.5 (104.0, 109.1)
UNIMMAP	93	115.0 (112.3, 117.8)	88	107.9 (105.6, 110.2)	77	105.2 (103.0, 107.4)
Drink powder	104	113.6 (111.1, 116.1)	92	108.7 (106.5, 111.0)	84	106.9 (104.7, 109.2)
**Diastolic Blood Pressure** (mmHg)						
Control	100	70.1 (68.3, 72.0)	93	68.4 (66.9, 69.9)	82	68.3 (66.6, 70.0)
UNIMMAP	93	71.0 (68.9, 73.1)	88	68.1 (66.5, 69.7)	77	67.9 (66.3, 69.6)
Drink powder	104	68.7 (66.8, 70.5)	92	67.5 (65.8, 69.3)	84	67.6 (66.0, 69.3)
**Pulse (beats per minute)**						
Control	100	72.3 (70.2, 74.5)	93	75.4 (73.1, 77.7)	82	77.3 (75.0, 79.8)
UNIMMAP	93	72.2 (69.5, 74.9)	88	74.5 (71.9, 77.1)	77	74.9 (72.6, 77.4)
Drink powder	104	72.0 (69.9, 74.1)	92	73.9 (71.9, 75.8)	84	76.7 (74.2, 79.3)

Abbreviations: CI, confidence interval; UNIMMAP, Daily United Nations Multiple Micronutrient Preparation supplement.
