# Supplementary material for: A novel nutritional supplement to reduce plasma homocysteine in nonpregnant women: A randomised controlled trial in The Gambia
Source: PLoS Med. 2019 Aug 13;16(8):e1002870. doi: 10.1371/journal.pmed.1002870 (PMC6691988; doi:10.1371/journal.pmed.1002870)
Supplement: S3 Table — (PDF) [file pmed.1002870.s006.pdf]

S3 Table: Additional details on morbidity

	UNIMMAP					Drink Powder				
**Adverse Event**	**N**	**Mean (days)**	**Median (days)**	**Min (days)**	**Max (days)**	**N**	**Mean (days)**	**Median (days)**	**Min (days)**	**Max (days)**
Nausea	104	0.26	0	0	5	93	0.21	0	0	7
Dizziness	104	0.37	0	0	10	93	0.30	0	0	6
Urine discolouration	104	0.05	0	0	2	93	0.37	0	0	8
Abdominal pain	104	0.58	0	0	19	93	0.46	0	0	14
Fever	104	0.07	0	0	2	93	0.18	0	0	7
